# Atom-scale contributions to nanofriction response in transition metal dichalcogenides

**DOI:** 10.3762/bjnano.17.92

**Published:** 2026-10-02

**Authors:** Suresh Ravisankar, Ravikant Kumar, Antonio Cammarata, Thilo Glatzel, Tomas Polcar

**Affiliations:** 1 Department of Control Engineering, Faculty of Electrical Engineering, Czech Technical University in Prague, Technicka 2, 16627 Prague 6, Czech Republic; 2 Department of Physics, University of Basel, Klingelbergstrasse 82, 4056 Basel, Switzerland

**Keywords:** deep neural network, density functional theory, Fourier transform, machine learning force field, molecular dynamics, nanofriction, nonmonotonic, transition metal dichalcogenides

## Abstract

We investigate the nanoscale friction behaviour of MX_2_ monolayers (M = Mo, W; X = S, Se) on Au(111) and Ag(111) substrates with a silicon tip using classical molecular dynamics simulations with machine-learning-based force fields. This approach enables an accurate description of tip–surface interactions and friction mechanisms at the atomic scale. We observe a pronounced nonmonotonic dependence of the friction force on the applied normal load, with the corresponding coefficient of friction being inversely proportional to the load. Analysis of lateral force signals and their spatial Fourier transforms reveals the coexistence of multiple sliding modes, including longitudinal sliding, lateral slip, and zig-zag motions. We show that the overall friction response is governed by the relative contributions of these motions. While the qualitative features of friction are largely substrate-independent, both the magnitude of friction and the balance between sliding modes depend sensitively on the substrate–monolayer combination. In particular, the Au/MoSe_2_/Si system exhibits significantly reduced friction due to suppression of lateral slip motion. The analysis method employed in this study is also applicable to other material systems with different chemical compositions or sliding interface geometries. However, the peak positions and intensities are expected to vary with the chemical composition of the material; consequently, their interpretation may differ across different systems.

## Introduction

Friction arises whenever two contacting surfaces move relative to each other, leading to energy dissipation and material wear in mechanical systems. Unfortunately, liquid lubricants are ineffective in reducing friction under extreme conditions, such as high normal load, high temperature, and ultrahigh vacuum [1]. In recent years, two-dimensional layered materials such as graphene, boron nitride, and transition metal dichalcogenides (TMDs) have emerged as promising solid lubricants in achieving superlubricity on rough and worn surfaces in engineering applications [2–7]. Among them, TMDs stand out for their layered structure and weak van der Waals bonding between the layers [8–9], which facilitates interlayer shear, resulting in exceptionally low frictional properties in vacuum. In general, the friction force exhibits a load-dependent behaviour, which at the nanoscale may tentatively be described by a linear force–load relationship arising from the contact mechanics of single-asperity interactions. However, some experiments already demonstrated that a linear dependence does not always hold at the nanoscale [10], and other studies attributed the origin of such behaviour to the occurrence of different kinds of contributions [11–12]. For this reason, the dynamic behaviour of the TMD layers with atomic detail during sliding is subject of continuous study, due to the complex phenomena occurring concurrently under tribological conditions. The most extensively used and simple theoretical model of nanoscale friction is the Prandtl–Tomlinson model [13–14]. However, atomistic observation of lateral slips, dynamic deformation, and thermal fluctuations is not well captured by this model; indeed, advanced analysis based on the Fourier transform of the friction force helps to quantify these lateral movements properties [15–16]. The applicability of Fourier-transform-based analysis is not limited to a specific material system; indeed, it has been successfully employed across chemically and structurally distinct interfaces, such as graphene/graphene systems [17], demonstrating the importance of this analytical framework. The interaction between TMD layers and metallic substrates, together with the influence of a scanning probe tip, determines the overall nanoscale friction response. The atomically flat nature of metallic substrates helps to suppress out-of-plane fluctuations of the TMD layer, resulting in a more stable sliding interface and more reliable investigation of frictional mechanisms [18]. First-principles-based calculations have provided valuable insights into the electronic and structural properties of these interfaces [19–20]; however, their application to large-scale friction simulations is limited due to high computational cost [21–23]. Compared with first-principles calculations, classical molecular dynamics (MD) is a technique capable of simulating dynamic and kinetic problems such as sliding processes and frictional behaviour at larger scale and reduced computational load [24–26]. MD simulations are often employed using empirical interatomic potentials, such as ReaxFF [27–28] and reactive empirical bond-order (REBO) potentials [26,29–30]; nevertheless, the parametrisation of such potentials is challenging and time-demanding.

The advancement of machine learning (ML) techniques in materials science has significantly alleviated this challenge [31]. A variety of machine-learning-based force fields (MLFFs) have been proposed, such as the TensorMol [32], the neuroevolution potential (NEP) [33], Gaussian approximation potential (GAP) [34], and neural network potentials (NNPs) [35]. Among these approaches, neural networks are particularly attractive because of their theoretical capability to approximate complex multidimensional real-valued functions with arbitrary accuracy when properly designed and trained. Recent studies have shown that NNPs can achieve accuracies comparable to first-principles quantum mechanical calculations for both molecular and extended condensed-matter systems, while also exhibiting excellent size extensivity [36–37]. Consequently, MLFFs have been successfully implemented for a wide range of materials, including Ga_2_O_3_ [38], TiO_2_ [39], and transition metal dichalcogenides (TMDs) [40–41,36]. Among the available MLFF approaches, NNPs have emerged as one of the most widely adopted and promising frameworks for current materials research.

In this work, we study the friction response of MX_2_ monolayers (M = Mo, W and X = S, Se) deposited on Au(111) and Ag(111) substrates by means of classical MD simulations at different loads and velocities of a scanning tip, employing MLFFs developed for this purpose. Our Fourier analysis of the friction signal allows us to quantify and characterise the nanoscale contributions to the friction response. In particular, we examine the atomistic phenomena contributing to the nanofriction response; this results in a nonmonotonic relationship between friction force and applied load, and contributes to significant uncertainty in the extracted coefficient of friction (COF) from a linear fit. Significant contribution originates from the tip’s lateral motion, which also reduces the average friction force, except in the Au/MoSe_2_/Si system, as the lateral motion is missing from the force profile.

## Computational Details

Density functional theory (DFT) based calculations for the structural optimisation of TM/MX_2_/Si systems (TM = Au, Ag; M = Mo, W; and X = S, Se) are performed using the Vienna ab initio simulation package (VASP) [42]. The interactions between valence electrons and ionic cores are described using the projector augmented-wave (PAW) method [43]. The generalized gradient approximation (GGA) in the Perdew–Burke–Ernzerhof (PBE) form is employed to treat the exchange-correlation energy [44]. The electronic self-consistent field calculations are converged to within 10^−8^ eV, and the atomic positions are relaxed until the residual forces in each atom are less than 10^−2^ eV/Å. A 7 × 7 × 1 Monkhorst–Pack **k**-point mesh [45] is used for Brillouin zone sampling, and the plane-wave basis set is truncated at a kinetic energy cutoff of 500 eV. To accurately capture long-range dispersion interactions, the Grimme DFT-D3 correction [46] with zero damping is incorporated to account for van der Waals forces between the layers. The ML potential is trained using the DeepMD-kit package with the Deep Potential-Smooth Edition (DeepPot–SE) model [47–48]. The model includes an embedding network and a fitting network. The sizes of these networks are set to (25, 50, 100) and (240, 240, 240), respectively. The cutoff radius is set to 8.0 Å, and the descriptors decay smoothly from 0.5 to 8.0 Å. The initial learning rate is set to 0.001 at the beginning of the training process to achieve a final value of 10^−8^. The total number of training batches are 10^5^ for the training in the initial iterations and the active learning process. To automate the active learning process, the DP-GEN workflow is used [49]. All molecular dynamics simulations are performed using the Large-scale Atomic/Molecular Massively Parallel Simulator (LAMMPS) [50] package through the trained ML potentials. The optimised structure of each system is equilibrated at 300 K for at least 4 ns with a time step of 1 fs by means of a Nosé–Hoover chain with three thermostats. To construct TM/MX_2_/Si interfaces, the TM substrate is cleaved from bulk Au and Ag as reported in [51] and subsequently optimised within the DFT framework.

**Figure 1:**
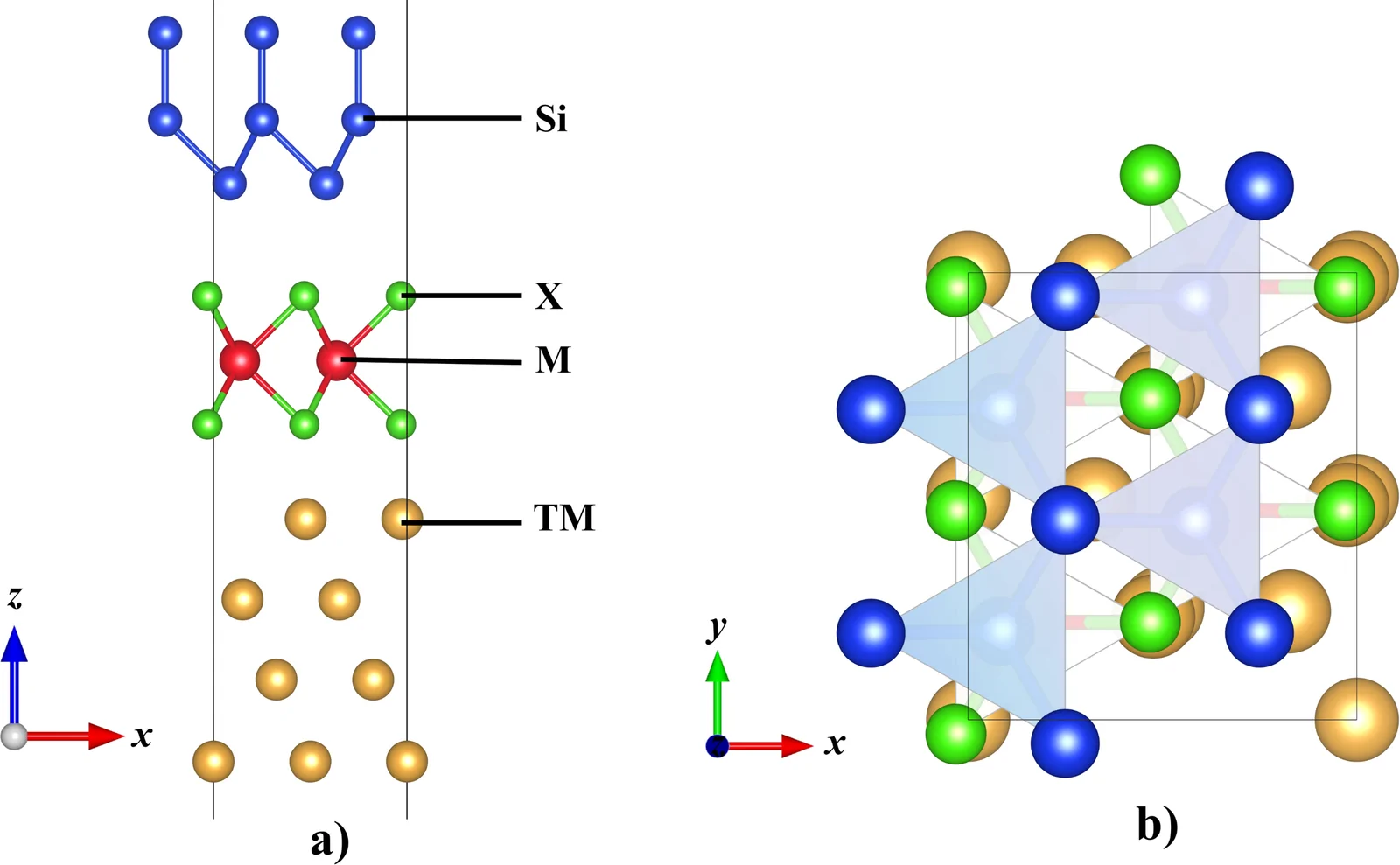
Initial setup to generate the structure model of TM/MX_2_/Si. (a) Front view and (b) top view. The thin black lines indicate the unit cell.

Next, a TMD monolayer is placed on top of the TM substrate in such a way to minimise the lattice mismatch of the resulting heterostructure. The lattice mismatch of the heterostructure is calculated to be 4.68% along the *x* direction and 5.82% along the *y* direction. A 30 Å vacuum slab orthogonal to the *c* direction is added on top of the geometry to prevent any interaction of the periodic replicas along the same direction; the geometry of the heterostructure is then relaxed. During the optimisation, the bottom layer of the TM substrate is fixed to better mimic the experimental conditions; finally, a Si (111) tip is placed above the heterostructure and the whole system is relaxed. In this last geometric optimisation, the bottom TM layer is kept fixed, while the Si tip is constrained to relax only along the *c* direction. The final optimised cell parameters are 5.189 and 5.976 Å. The Si tip extends laterally in the *x*–*y* plane across the whole size of the unit cell. With this choice, we want to analyse the frictional behaviour of the system in the inner region of the tip far from the edges. The expanded simulation cell used in the friction calculations is described in Supporting Information File 1, Section I, Structural Information. An example of model geometry of the full system is reported in Figure 1.

### Training of the force field

**Figure 2:**
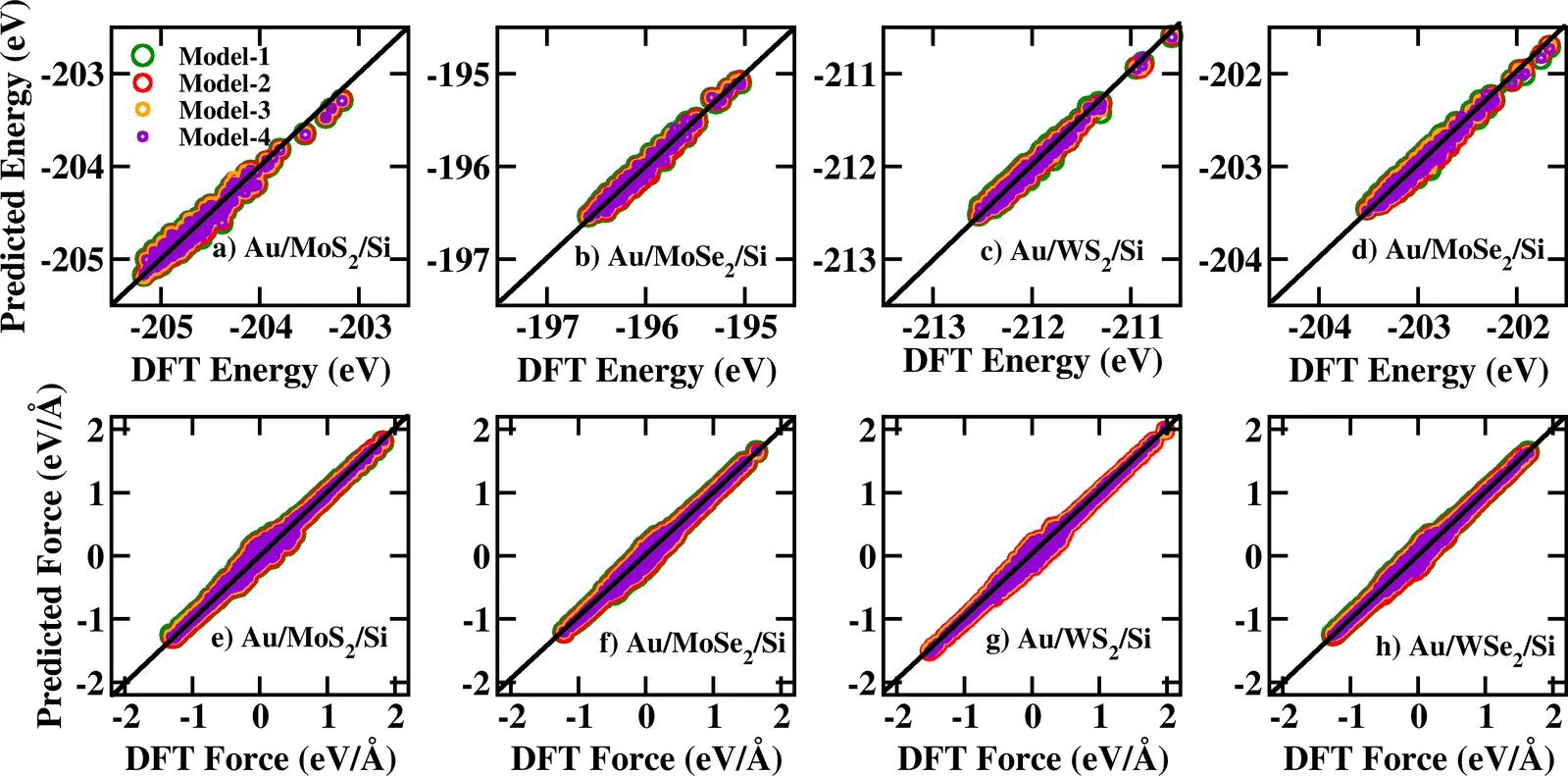
Energy (a–d) and force (e–h) comparisons between predicted and DFT results for Au/MX_2_/Si systems.

**Figure 3:**
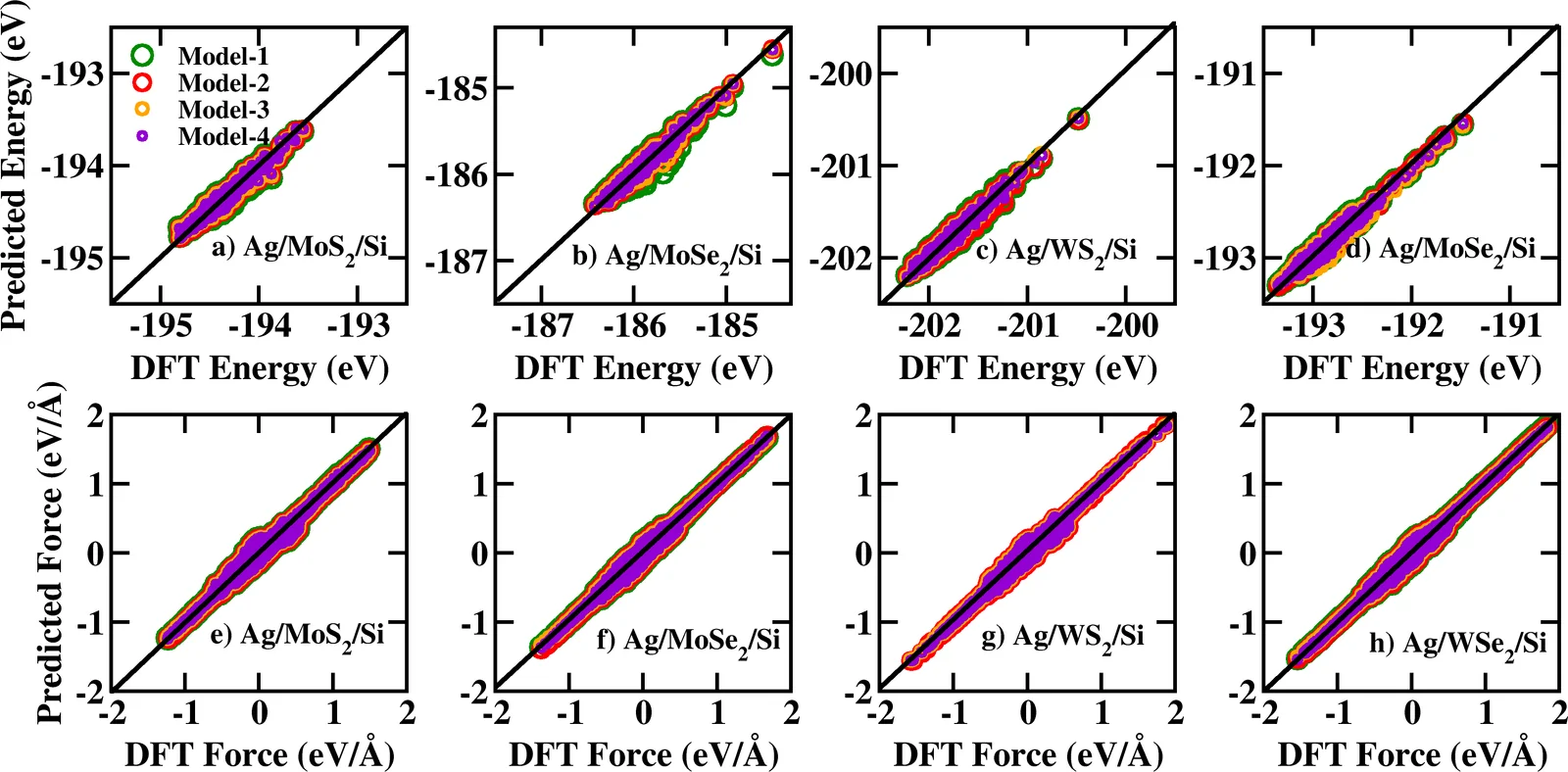
Energy (a–d) and force (e–h) comparisons between predicted and DFT results for Ag/MX_2_/Si systems.

After optimising the full Au/MX_2_/Si configuration, we proceed to construct the MLFF for the system. Once we obtain the optimised structure, the training data for the MLFF are generated by perturbing the atom positions and cell size by at least 0.01 Å and 0.03 Å, respectively. A total of ≈400 configurations are generated to train the MLFF of all the systems and 80 configurations are taken for the validation during the active learning process. For all configurations, energy and forces are calculated with the selected DFT setup and a single point calculation, that is, at fixed atom geometry and lattice parameters. To effectively improve the MLFF, additional data are generated using an active learning approach as proposed by Zhang and coworkers [49]. The training is performed using an active learning procedure, which iteratively identifies and incorporates the most informative configurations into the training set based on the predictive uncertainty of the model. This approach selectively extracts configurations that exceed a predefined uncertainty threshold, ensuring that only those configurations that meaningfully contribute to improving the energy fit tolerance are selected for DFT calculation and included in the training set [52–53]. At each iteration, four models are trained using the same input setup but starting with different random seeds. To explore new configurations to enrich the dataset, deep potential molecular dynamics (DPMD) simulations are performed with the initial models as reference, collecting the system’s atomic configurations every 10 fs along the trajectories. DPMD simulations are performed for a duration of 500 ps. During the DPMD simulations, the force acting on each atoms in all configurations are evaluated by all four models. The maximum standard deviation of the atomic forces σ_max_ is evaluated as a criterion for the convergence of neural network training,


[1] $$\sigma_{\max}=\max_{i}\sqrt{\langle {\left\|f_{i}-\langle f_{i}\rangle \right\|}^{2}\rangle },$$

where **f***_i_* is the force acting on *i*-th atom and ⟨**f***_i_*⟩ is the average value taken from four models. A structure is considered a candidate if the maximum standard deviation falls within the range of 0.05 *<* σ_max_ *<* 0.15 eV/Å; while structures with σ_max_ *<* 0.05 eV/Å are considered correct, those with σ_max_ *>* 0.15 eV/Å can be highly distorted configurations and are discarded. DFT calculations are carried out for the selected structures to obtain the corresponding energies and forces, which are included in the dataset. The training with four new models is repeated until the root mean square error (RMSE) value is reduced. The RMSE values of energy and forces for all the systems are presented in Table 1. All the final models have RMSE values of energy and forces ranging from 0.919 to 1.579 meV/atom and from 0.019 to 0.021 eV/Å, respectively. This range of RMSE values indicates that the MLFFs model is properly trained with values for the errors typical of surface and interface systems [40–41]. To validate our MLFFs, we optimise the geometry of the heterostructures by using the trained MLFFs; we then compute the RDF scalar products with the DFT-optimised structure using the MAISE package [54], in order to check if the two methods yield the same optimised geometry. This analysis shows that the structures optimised with the two methods are almost identical, then confirming the reliability of the MLFFs parametrisation. The same procedure is applied to the case of the Ag substrate. The predicted energies and forces obtained from the machine-learning force field show very good agreement with the reference DFT results (Figure 2 and Figure 3). This agreement further validates the accuracy of the trained models.

**Table 1:** The RMSE value of all the trained models.

Monolayer	Substrate	Energy RMSE (meV/atom)	Force RMSE (eV/Å)	RDF Scalar product

MoS_2_	Au	1.579	0.021	0.904
MoSe_2_		1.028	0.020	0.892
WS_2_		0.919	0.019	0.946
WSe_2_		1.178	0.019	0.941

MoS_2_	Ag	1.330	0.020	0.879
MoSe_2_		0.941	0.019	0.878
WS_2_		0.989	0.020	0.947
WSe_2_		1.298	0.020	0.920

### MD setup for friction analysis

**Figure 4:**
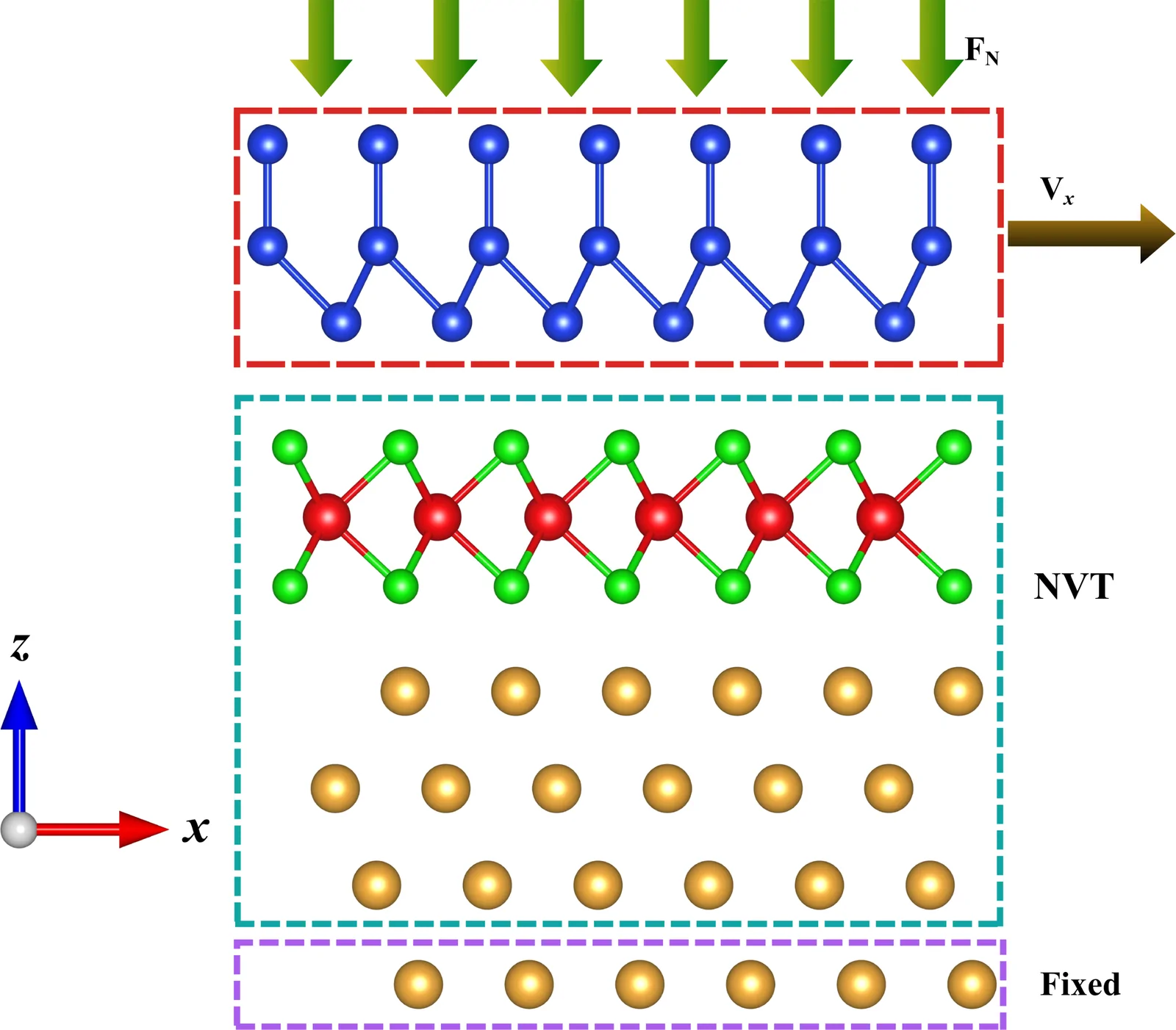
MD simulations setup.

For the MD simulations, a 3 × 3 × 1 supercell of the optimised heterostructure was constructed in order to minimise the effects of the system’s finite size and to provide a sufficiently large contact area for the sliding process (see Figure 4); based on preliminary benchmarks, this model choice represents a good compromise between reliability of the results and computational feasibility for the scope of this study. The resulting simulation cell contains a total of 360 atoms (See the POSCAR data in Supporting Information File 1, Section I). The bottom layer of the TM substrate is kept fixed throughout the simulation. This constraint prevents artificial translation of the entire system and represents the bulk support typically present in experimental setups; the remaining atoms are allowed to evolve dynamically during the simulation in the NVT ensemble at a temperature equal to 300 K. The Si layers are treated as a rigid body, which represents a limitation of the present model as it does not account for the flexibility and compliance of the tip, to simulate a rigid scanning tip. The tip is moved along the *x* direction at constant velocities of 2, 3, 4 and 5 m/s, while there is no constraint on the motion along the *y* and *z* directions. MD simulations are inherently limited by accessible timescales due to the femtosecond integration time step required to accurately resolve atomic vibrations, which implies the computation of a large amount of dynamics steps to obtain meaningful simulated time windows of the order of nanoseconds. In contrast, experimentally relevant sliding processes often occur over much longer timescales, ranging from microseconds to seconds. Consequently, performing simulations at experimental sliding velocities would require prohibitively long runtimes and substantial computational resources to achieve adequate statistical sampling. To address this challenge, atomistic simulations commonly employ sliding velocities that are several orders of magnitude higher than those used experimentally. This approach enables the efficient observation of key atom-scale phenomena, including structural rearrangements, energy dissipation mechanisms, and frictional responses within computationally tractable timescales. Although the applied velocities exceed experimental values, previous studies have shown that the fundamental atomistic mechanisms governing the friction response can still be captured reliably, provided that the results are interpreted with consideration of the rate-dependent nature of these processes. Therefore, the use of high sliding velocities represents a necessary and widely accepted compromise between computational efficiency and physical realism, enabling meaningful investigation of the underlying mechanisms while maintaining feasible simulation costs [24,27,26,55–61]. During sliding, normal loads of 0.0, 0.1, 0.2, 0.3, 0.4, 0.5, 0.6, 0.7 and 0.8 nN are applied to the tip along the −*z* direction. Throughout the simulation, the instantaneous forces acting on the tip and the atomic trajectories are recorded at every 1 fs time step.

## Results and Discussion

### Friction force analysis

**Figure 5:**
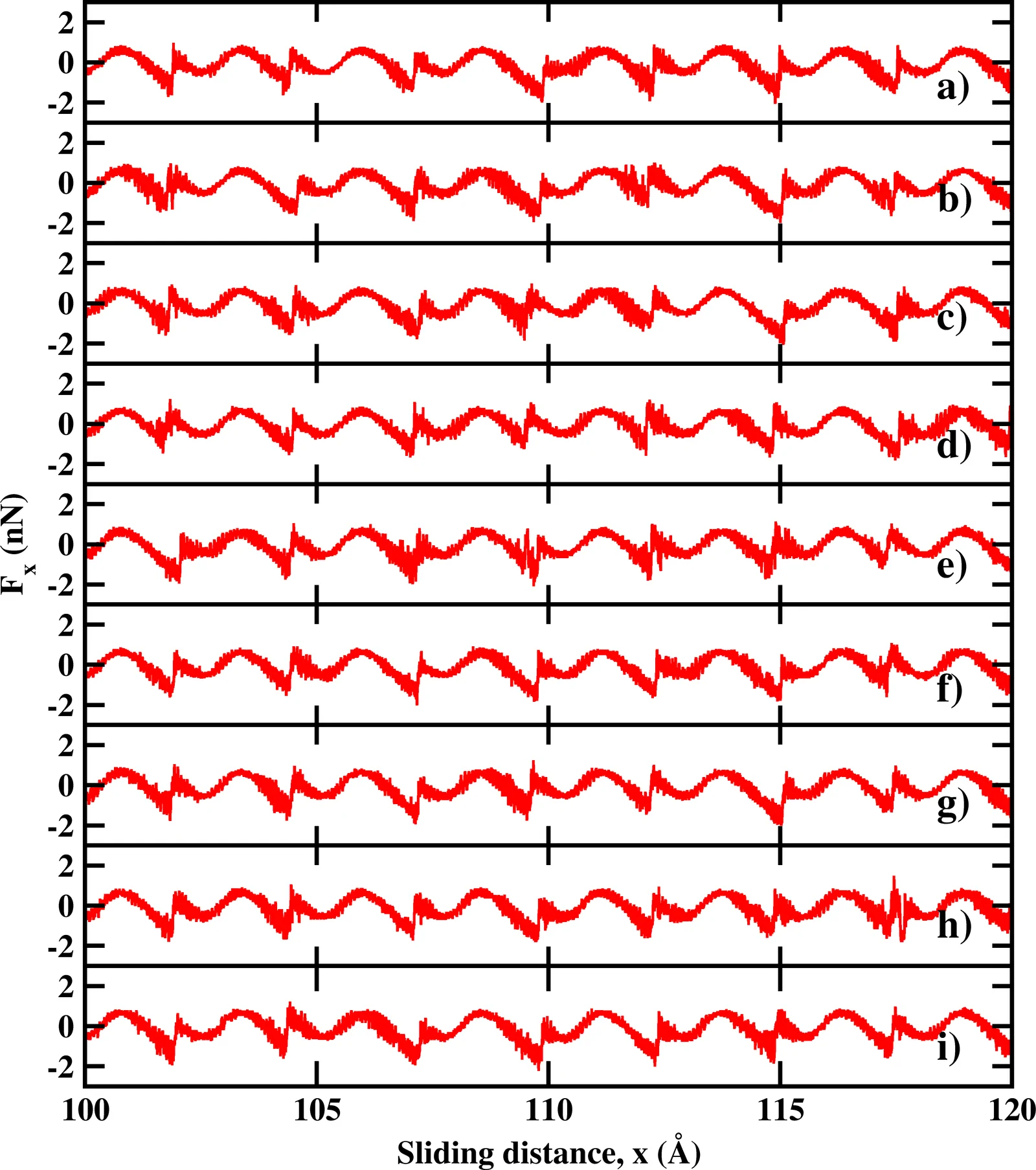
Friction force profile of Au/MoS_2_/Si with tip moving at 2 m/s for under loads of (a) 0 nN, (b) 0.1 nN, (c) 0.2 nN, (d) 0.3 nN, (e) 0.4 nN, (f) 0.5 nN, (g) 0.6 nN, (h) 0.7 nN, and (i) 0.8 nN.

**Figure 6:**
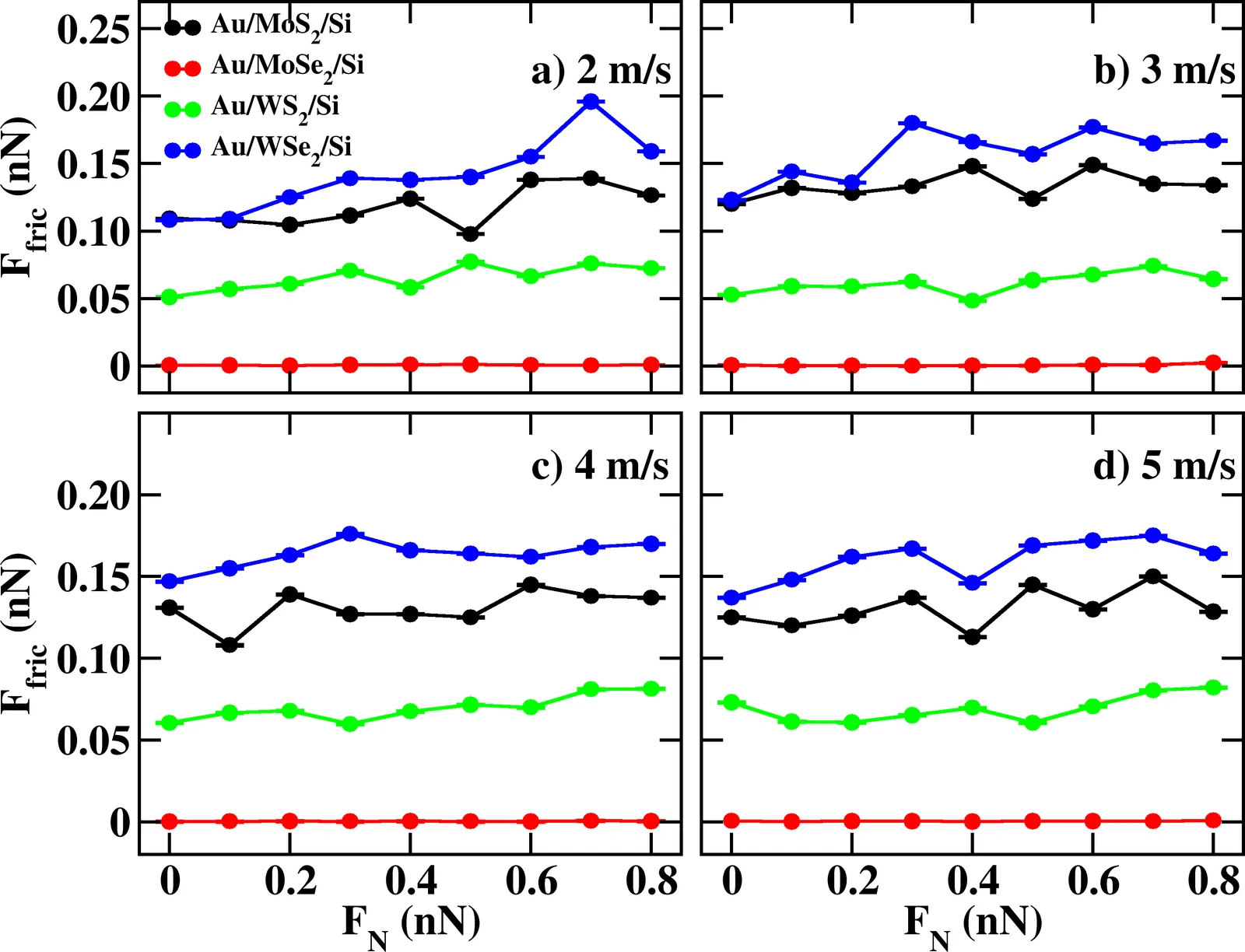
Average friction force as a function of applied load for all systems at tip velocities of 2, 3, 4, and 5 m/s. Lines are a guide to the eye. The error bars, which are very small, denote the uncertainty in the calculated friction force.

We begin our study of the friction response by considering the friction force profile using 1 ns trajectories under different applied normal loads, as shown in Figure 5. Interestingly, the overall shape of the friction force profile remains nearly identical across all applied loads, indicating negligible load dependence of the profile shape. We then calculate the average friction force acting on the Si tip along the *x* direction *F*_fric_, by averaging over all the snapshots of the trajectory:


[2] $$F_{\text{fric}}=-\frac{1}{N_{\text{s}}}\sum_{i=1}^{N_{\text{s}}}\sum_{j=1}^{N_{\text{tip}}}F_{xj}\left(t_{i}\right),$$

where *N*_s_ is the number of time steps, *N*_tip_ is the number of atoms in the tip, *F_xj_* is the force acting on the *j*-th atom of the tip at the *t_i_* time step along the sliding *x* direction, and the negative sign accounts for the fact that friction opposes the direction of motion. The coefficient of friction μ is then calculated according to the force–load relationship with the following linearly fitting function:


[3] $$F_{\text{fric}}=\mu F_{\text{N}}+F_{\text{fric}}^{0},$$

where *F*_N_ is the applied normal load and 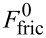 is the friction in the absence of load. The calculated average friction force at varying loads with different velocities for all the systems are reported in Figure 6. Among all systems, Au/WSe_2_/Si system exhibits the highest friction force across all velocities, followed by Au/MoS_2_/Si and Au/WS_2_/Si. Au/MoSe_2_/Si displays nearly negligible friction force across all the applied normal loads. This behaviour is consistent with the corresponding friction force profile, which will be discussed in detail below. In most cases, the friction–load relationship deviates from simple linear behaviour, exhibiting nonmonotonic trends regardless of the velocities.

**Figure 7:**
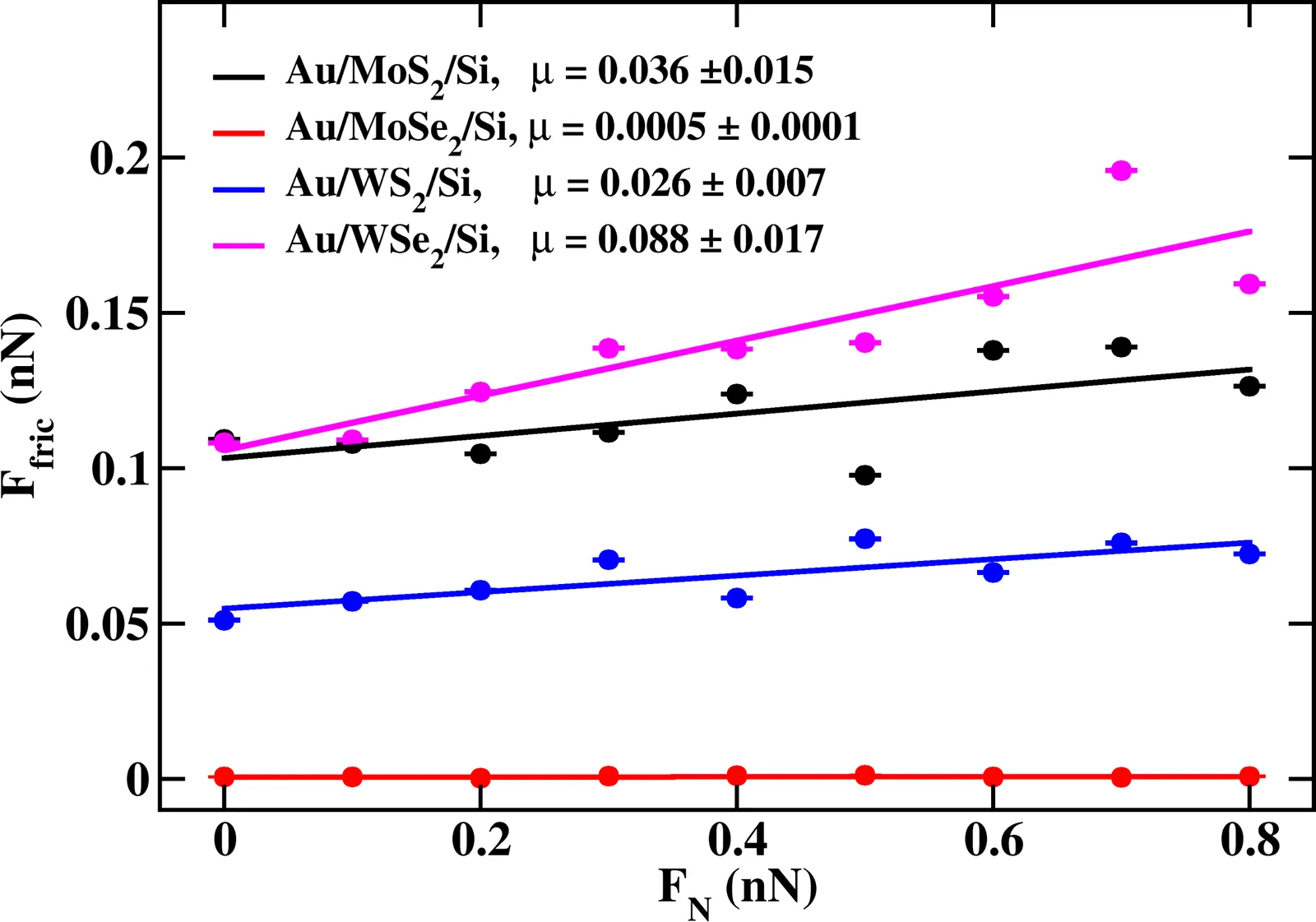
COF obtained from a linear fit of the average friction force as a function of the applied load for all systems at a tip velocity equal to 2 m/s. The error bars denote the uncertainty in the calculated friction force.

Looking at Figure 7, which represents the fitted curve corresponding to Figure 6a, the fit analysis gives μ values equal to 0.036, 0.026, 0.088, and 0.0005, with corresponding errors of 42%, 26%, 19%, and 20% for Au/MoS_2_/Si, Au/WS_2_/Si, Au/WSe_2_/Si, and Au/MoSe_2_/Si, respectively. The extracted μ values show relatively large errors, indicating that the assumption of a linear friction–load relationship is not strictly valid. A similar trend is observed at other velocities (see Supporting Information File 1, Section II, Figures S1–S3). This behaviour suggests that, in our systems, a simple proportional relationship between *F*_fric_ and *F*_N_ cannot fully describe the friction response. This has already been experimentally observed for a MoS_2_ monolayer grown on a Au surface [62]. In this work, friction force microscopy measurements on MoS_2_/Au(111) demonstrated a nonmonotonic velocity dependence associated with a transition between single-slip and multiple-slip atomic regimes, experimentally observed within the velocity range of 10–1000 nm/s. This regime is fundamentally different from the MD simulations presented in the current work, which were performed at significantly higher sliding velocities (2–5 m/s); consequently, a direct quantitative comparison is not straightforward. Nevertheless, this experimental study and the present one identify multiple slip events as relevant contributions to the frictional response.

It is known that the COF is not a material constant in general; it rather exhibits a dependence on the applied normal load [63], reflecting the interplay between adhesive and repulsive interfacial interactions. In particular, the presence of adhesion at the nanoscale contact introduces a non-trivial load dependence that deviates markedly from the classical picture, where the COF is treated as load-independent. We then extend our analysis by considering the COF μ′ (where μ′ = *F*_fric_/*F*_N_) as a function of the applied normal load:


[4] $$\mu^{\prime }\left(F_{\text{N}}\right)=\mu_{\infty }+\frac{A}{F_{\text{N}}},$$

where μ_∞_ is the asymptotic COF at very high load and *A* is a fitting parameter in the unit of force. To understand the physical meaning of *A*, we multiply both sides by *F*_N_, thus obtaining


[5] $$\mu^{\prime }\left(F_{\text{N}}\right)F_{\text{N}}=\mu_{\infty }F_{\text{N}}+A,$$

where the quantity μ′(*F*_N_)*F*_N_ is the friction force. The friction force comprises two distinct contributions: The first term, μ_∞_*F*_N_, represents the proportional contribution of the load to the friction force via the asymptotic friction coefficient μ_∞_; the second term, *A*, is a load-independent constant that can be determined by extrapolating the friction force to the limit of zero normal load. Physically, the parameter *A* represents the adhesive component of friction, arising from interfacial interactions such as van der Waals forces, electrostatic interactions, and chemical bonding at the contact interface. These interactions collectively give rise to a finite real contact area and consequently a finite friction force even in the absence of any externally applied load. The existence of this adhesive term is fully consistent with the Bowden–Tabor adhesion theory of friction, which explicitly recognizes that the real contact area is not strictly proportional to the applied normal load, but rather reflects the intrinsic adhesive nature of the contacting surfaces [64]. It is important to note that the load-independent fitting parameter *A* cannot be attributed to finite-size or edge effects. In our simulations, periodic boundary conditions are applied in the lateral directions, effectively representing an infinite interface and eliminating border interactions during sliding. As a result, the AFM tip never encounters a finite boundary, and any load-independent contribution arising from finite-size artifacts is absent. Therefore, the parameter *A* can be interpreted as an intrinsic contribution associated with the adhesive interactions at the sliding interface. By fitting the computed COF data at each load with the proposed empirical expression (Equation 4), we are able to disentangle the adhesive and load-dependent contributions to the frictional response, and to quantify how the relative weight of each contribution evolves as a function of the applied normal load. This provides a direct and physically meaningful connection between the atomistic friction mechanisms identified through our MD simulations and the continuum level tribological framework. Notably, the data point corresponding to *F*_N_ = 0 nN is excluded from the fitting procedure because μ′ = *F*_fric_/*F*_N_ is undefined at zero normal load. The fitting is therefore performed using only the non-zero load data points.

**Figure 8:**
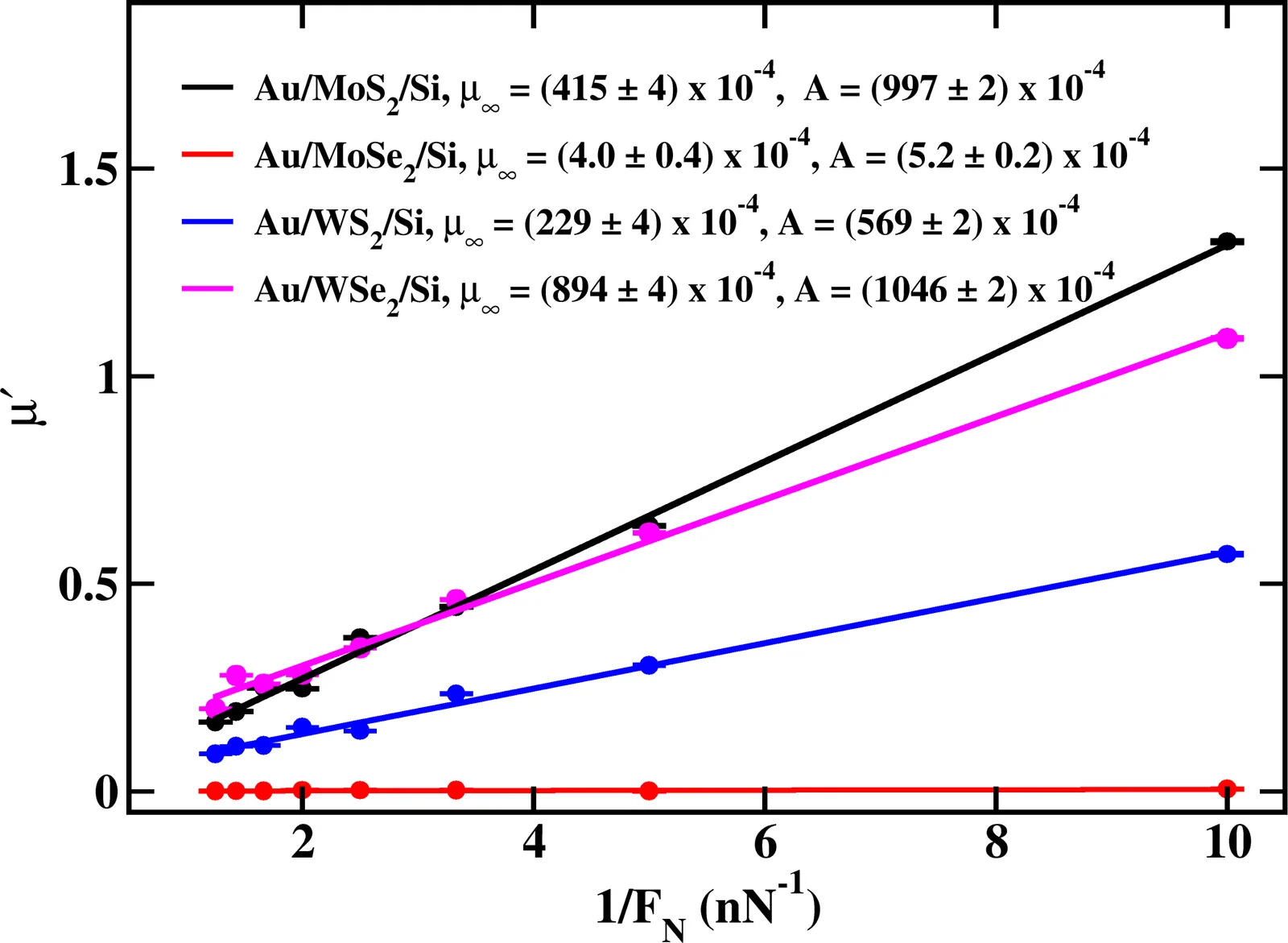
COF as a function of inverse applied load for all the systems with a tip velocity of 2 m/s. Note that the data point at *F*_N_ = 0 nN is excluded from the fit, as μ′ = *F*_fric_/*F*_N_ diverges at zero load.

Figure 8 shows a linear fit of μ′(*F*_N_) as a function of the inverse normal load (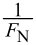) for all the systems with tip velocity equal to 2 m/s. The fitted asymptotic μ_∞_ values for Au/MoS_2_/Si, Au/WS_2_/Si, Au/WSe_2_/Si, and Au/MoSe_2_/Si are 415, 229, 894 and 4.0 of the order of 10^−4^, respectively, with absolute error of the order of 10^−4^. Similar trends are observed at other velocities as mentioned in section “Frictional properties” of Supporting Information File 1. The corresponding adhesive fitting parameters *A* are determined as 997, 569, 1046, and 5.2 of the order of 10^−4^ nN, respectively, within the same error range. We notice here that the use of Equation 4 provides a fit for the parameters with very small errors in comparison with the linear fit using Equation 3. Notably, the Au/MoS_2_/Si and Au/WSe_2_/Si systems exhibit large *A* parameters. This suggests that the adhesive interactions at the Au/MoS_2_/Si and Au/WSe_2_/Si interfaces are considerably stronger than those in the remaining systems. Consequently, the adhesion force exerts a dominant influence on the overall frictional response in these systems, manifesting as a pronounced load-independent contribution to the COF that cannot be attributed to the applied normal load alone. Furthermore, we plot μ′(*F*_N_) as a function of *F*_N_ using Equation 4, with results presented in Supporting Information File 1, Section II, Figure S12. If the friction–load relation were as in Amontons’s law, the friction coefficient μ′ would remain constant and independent of the applied load. However, our results reveal a clear decreasing trend of μ′ with increasing normal load, indicating a departure from this classical behaviour at the nanoscale. It is worth noting that the statistical errors associated with the fit of Equation 4 are comparable to those obtained from the linear fit of Equation 3, confirming that both equations represent algebraically equivalent parametrizations of the same underlying friction–load relationship, as discussed above. This finding underscores the critical role of interfacial adhesion in governing nanoscale friction, and highlights the necessity of explicitly accounting for the adhesive term when interpreting COF measurements across different TMD-based tribological systems.

**Figure 9:**
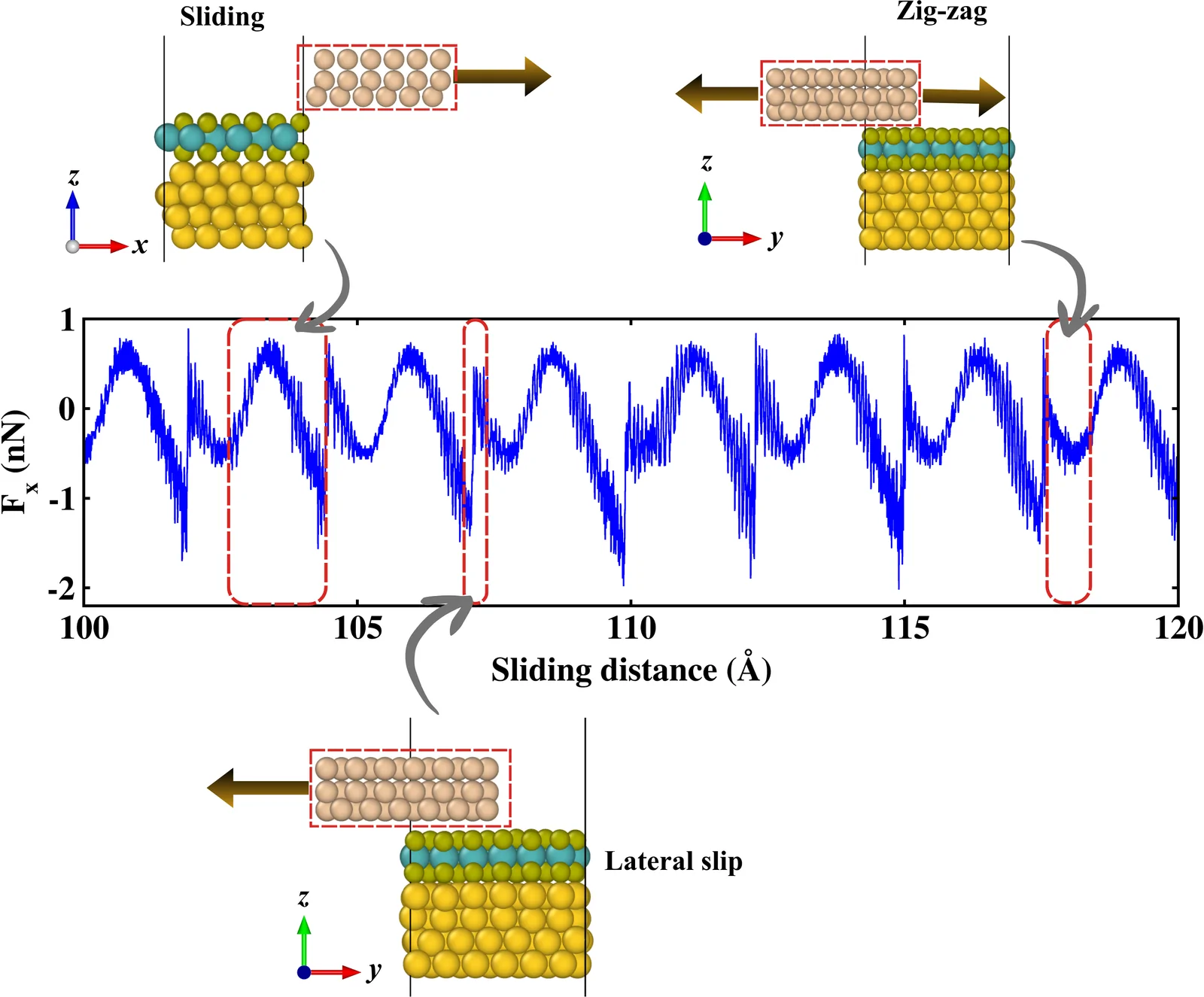
Friction force profile associated with the motion of the tip.

In addition, to understand the origin of this nonmonotonic behaviour, we look at the detail of the friction force on the tip during the sliding. Figure 9 shows the friction force on the Si tip as a function of the sliding distance for the case of Au/MoS_2_/Si system with a velocity of 2 m/s and a normal load of 0 nN. Apart from the sliding events along the sliding direction, some extra features can also be observed. Analysis of the geometric sequence along the trajectory reveals that large sudden drops in the lateral force correspond to slip events associated with the primary sliding motion along the *x* direction, named sliding. In contrast, abrupt increases in the force indicate lateral slip motion involving transverse displacement along the *y* direction, named lateral slip. Following these events, a gradual decrease in the force is observed, which corresponds to a zig-zag-like motion of the tip as it relaxes while moving laterally along the *y* direction, named zig-zag. Anisotropic response has already been reported in the work of Sheehan et al. [65], who have observed anisotropic frictional behaviour and contact-area dependent friction in layered metal dichalcogenides related to easy slip directions at the interface. These observations confirm that the tip does not follow a strictly one-dimensional sliding pathway; instead, the motion involves coupled displacements along both *x* and *y* directions, leading to the different frictional behaviour along the *x* direction. The zig-zag events indicate that the system explores multiple local minima on the interfacial potential energy surface during sliding; such lateral motion effectively modifies the instantaneous contact configuration and the energy dissipation pathway, leading to fluctuations in the lateral force along the *x* direction. Interestingly, these features in the force profile occur in most of the cases except the system Au/MoSe_2_/Si. In the Au/MoSe_2_/Si system, however, the second spectral peak associated with lateral slip is almost negligible, as shown in Supporting Information File 1, Section II, Figure S5, meaning that lateral slip events are essentially absent. Importantly, the first spectral peak, which represents the dominant contribution to the friction force, is also intrinsically weak in this system. Consequently, in this system, the low friction force originates from the inherently weak primary sliding contribution resulting from the low potential energy corrugation of the MoSe_2_ interface. When two surfaces interact, the surface interactions create a corrugated potential energy landscape that governs the lateral motion during sliding. As the tip moves across this landscape, the motion may not remain strictly confined to the imposed sliding direction; instead, the system can explore neighbouring energy minima, resulting in transverse displacements and complex sliding pathways.

**Figure 10:**
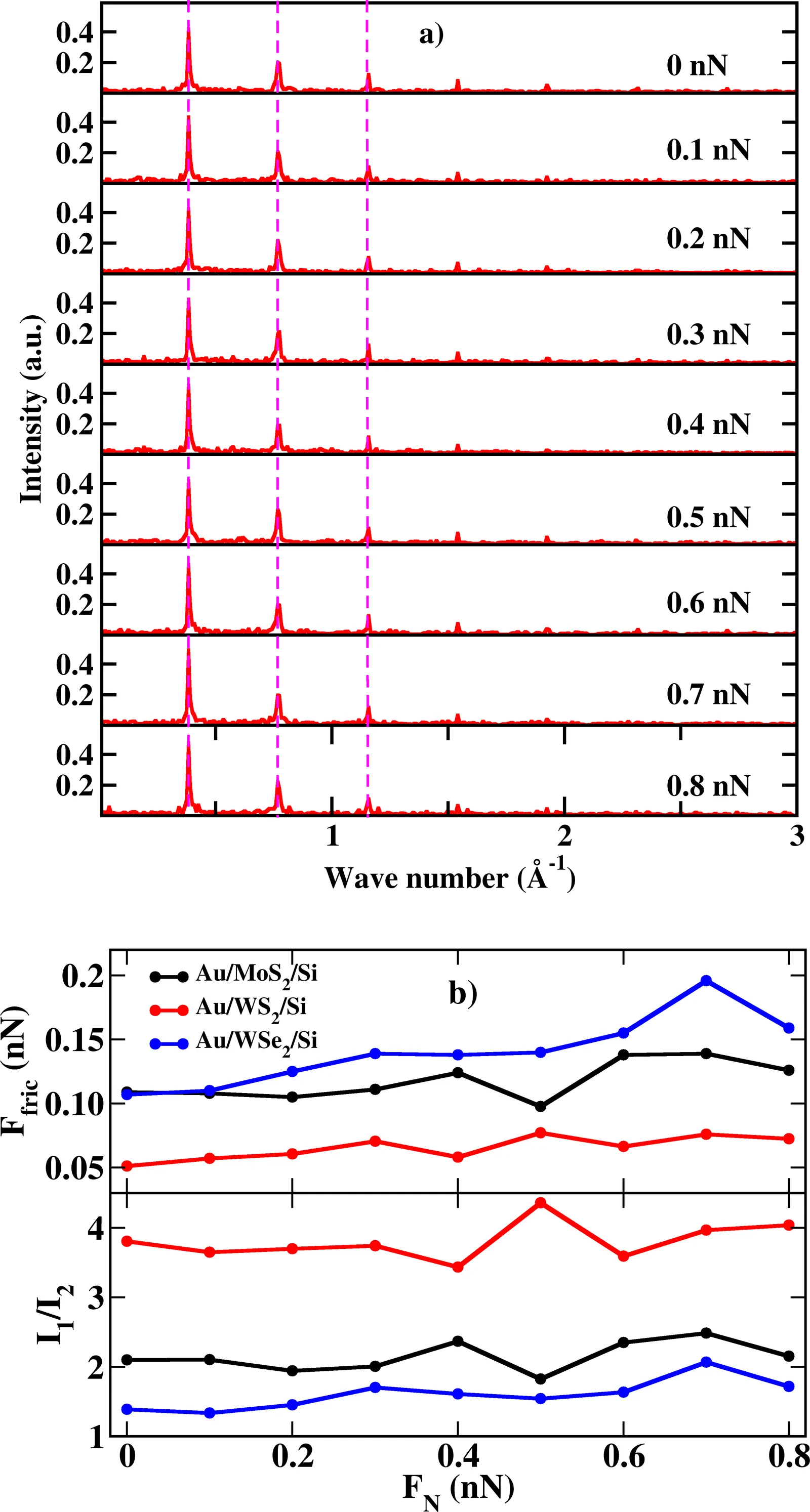
(a) Spatial Fourier transform of Au/MoS_2_/Si with varying loads at a velocity of 2 m/s. (b) Peak intensity ratio and their average friction force with varying loads for the systems indicated in the legend. Lines are a guide to the eye.

To quantitatively characterise these slip events, we perform a spatial Fourier transform of the forces experienced by the Si tip along the *x* direction. This analysis provides insight into the nature of the forces that arise from different modes of tip motion, including sliding, lateral motion, and possible nonlinearities in the tip trajectory. Figure 10a displays the spatial Fourier transform of the average force experienced by the tip for the case of Au/MoS_2_/Si at different normal loads with velocity of 2 m/s. Three magenta dotted lines indicate the main peaks at wave vectors **k**_1_, **k**_2_, and **k**_3_ with the corresponding intensities *I*_1_, *I*_2_, and *I*_3_. The positions of three peaks are identified at **k**_1_ *<* **k**_2_ *<* **k**_3_, corresponding to the values of 0.38 *<* 0.77 *<* 1.6 Å^−1^. Similarly, the positions of the spectral peaks **k**_1_ *<* **k**_2_ *<* **k**_3_ remain invariant across the entire range of investigated sliding velocities, as shown in Supporting Information File 1, Section II, Figure S11, confirming that the lateral slip and zig-zag motions are intrinsic features of the potential energy surface topology rather than artifacts of the elevated sliding velocities employed in the simulations. In the Fourier transform, the intensity of a peak is proportional to the number of times the corresponding event occurs, while the inverse of the peak position gives the width of the events on a distance scale. In our simulations, the first peak at **k**_1_ corresponds to the sliding process along the *x* direction, while the second and third peaks (at **k**_2_ and **k**_3_, respectively) correspond to lateral zig-zag movement and lateral slip along the *y* direction, respectively. We do not consider other peaks, as their intensity is almost as small as the background noise corresponding to thermal fluctuations of the atomic motions. This is confirmed from the friction force profile where the lengths of the events correlate to the peak positions. The Fourier transform spectra reveal a clear correlation between the peak intensities and the average friction force. In particular, the overall ratio *I*_1_/*I*_2_ of the first two peak intensities for all the systems increases as the mean friction force increases (Figure 10b).

This trend is observed for all systems across different velocities, except in the case of a Au/MoSe_2_/Si system; in fact, in this case only a single peak appears in the Fourier transform only at **k**_1_, that is, the peak of the sliding, making it impossible to establish a correlation based on the ratio of peak intensities (see Supporting Information File 1, Section II, Figure S5). This trend suggests that when the number of lateral slip events increases, the effective friction force decreases (Figure 10b). Since the friction force is evaluated under different normal loads, the relative contributions of these sliding modes vary with load. As a result, the mean friction force does not exhibit a strictly linear dependence on the applied load, which leads to the nonmonotonic behaviour observed in the friction–load relationship, and contributes to the large uncertainty in the extracted coefficient of friction.

### Effect of substrate on friction force

**Figure 11:**
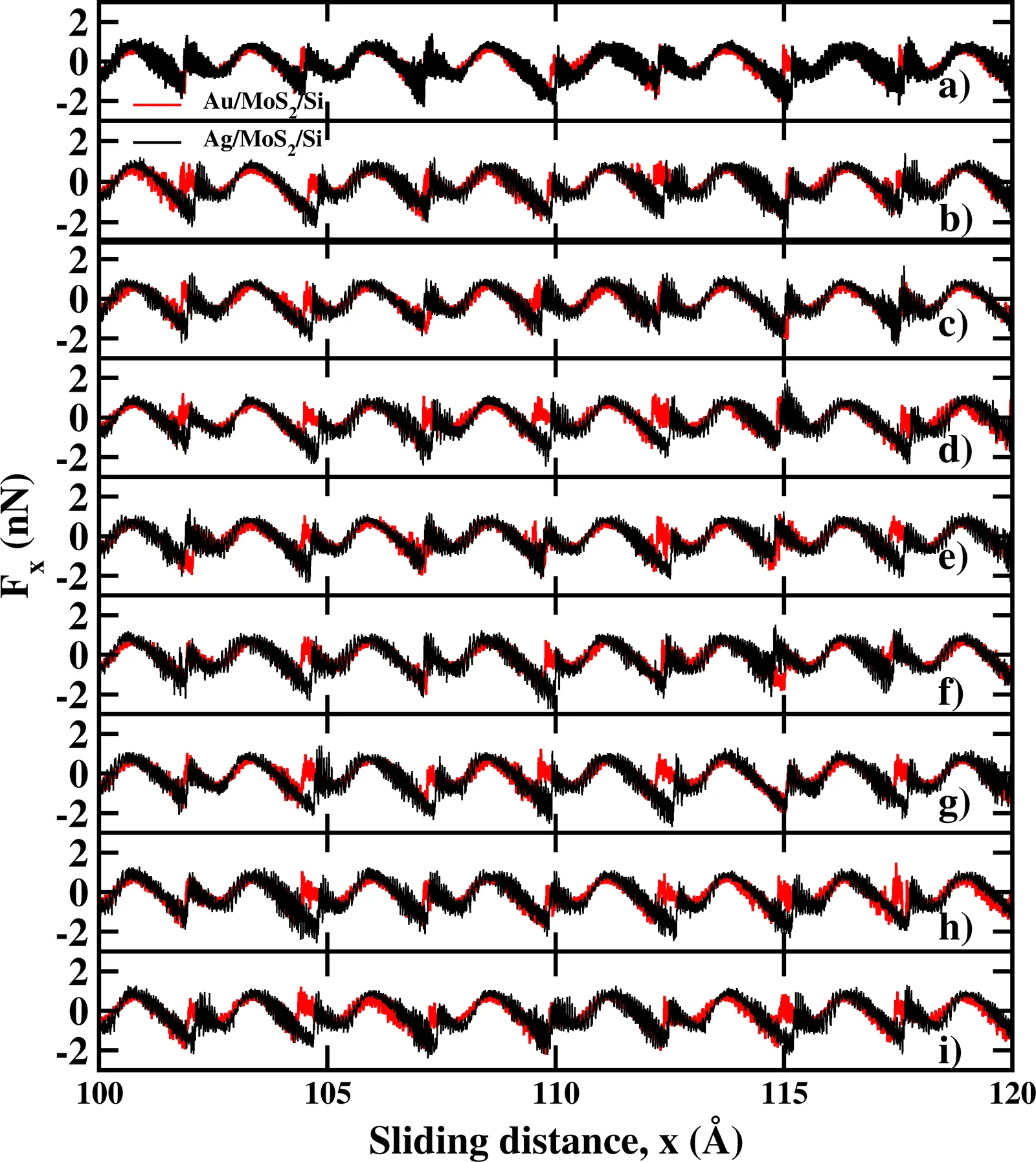
Comparison of friction force profile between Au/MoS_2_/Si and Ag/MoS_2_/Si as a function of displacement at loads (a) 0 nN, (b) 0.1 nN, (c) 0.2 nN, (d) 0.3 nN, (e) 0.4 nN, (f) 0.5 nN, (g) 0.6 nN, (h) 0.7 nN, and i) 0.8 nN.

To investigate the effect of the substrate on the lateral friction force, we analyse the trend of the force experienced by the tip as a function of sliding distance under different applied normal loads and different substrates, namely Au and Ag, as shown in Figure 11.

**Figure 12:**
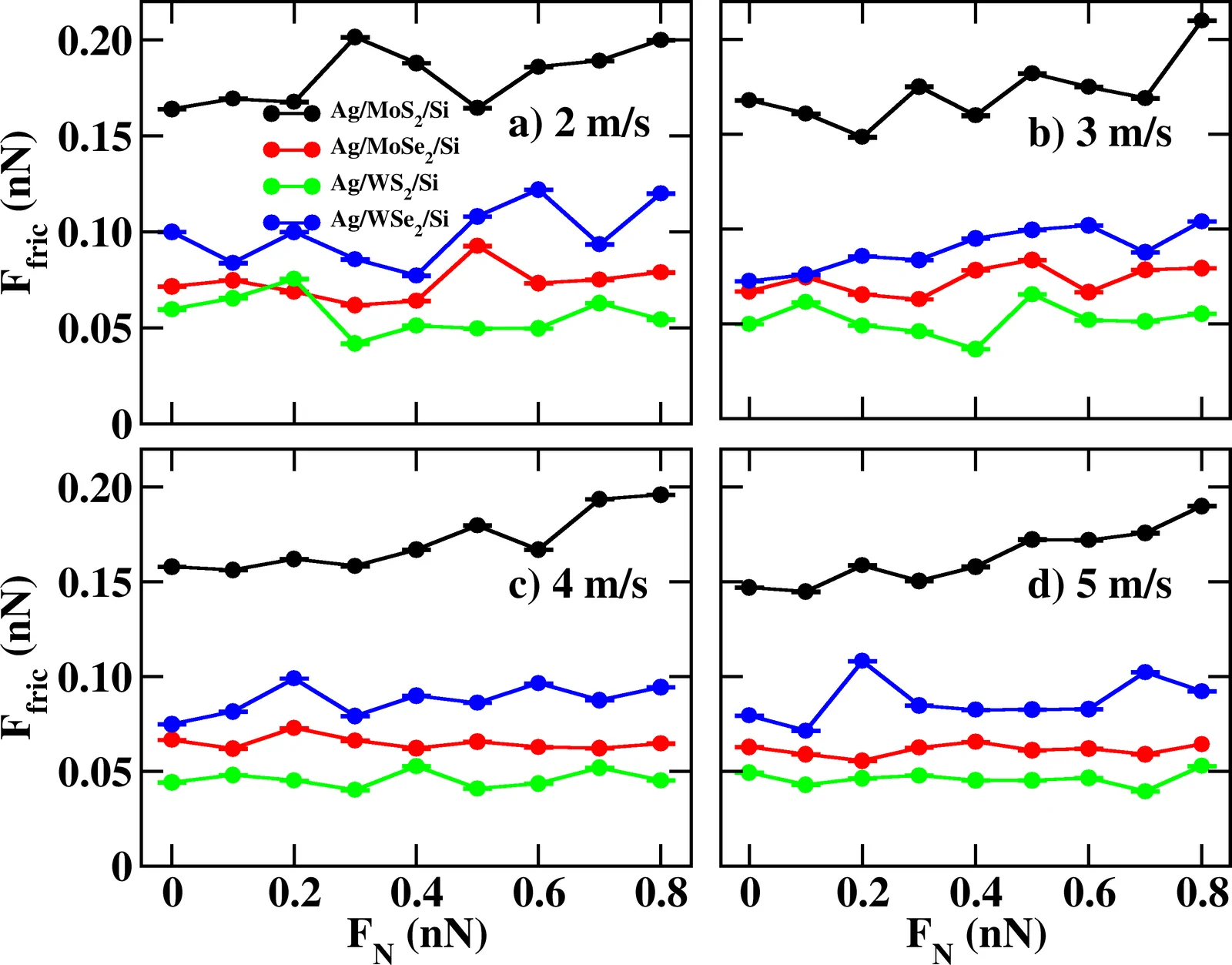
Average friction force as a function of applied loads for all the systems with tip velocities of 2, 3, 4, and 5 m/s. The error bars which are very small denote the uncertainty in the calculated friction force.

We observe that the overall nature of the force signal particularly the shape of the force profile remains essentially unchanged as the load increases, provided the monolayer and velocity are kept constant; moreover, in the presence of the Ag substrate, the average force on the tip follows a trend very similar to that observed for the Au substrate. Furthermore, we plot the average friction force as a function of the applied load for all systems and for all sliding velocities, in the presence of the Ag substrate, as shown in Figure 12.

**Figure 13:**
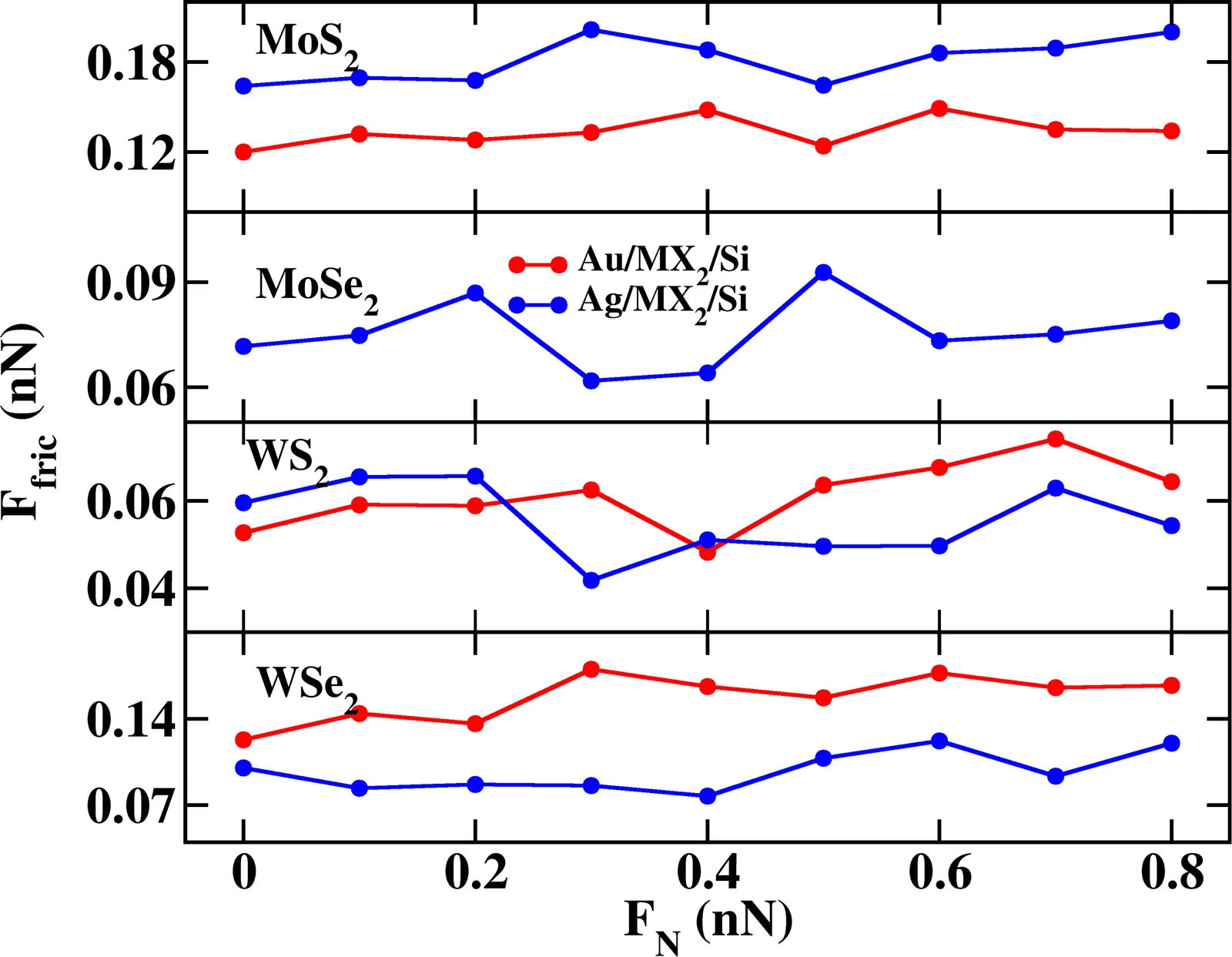
Average friction force, varying with loads and different substrate for a velocity of 2 m/s. The Au/MoSe_2_/Si system has not been considered.

We found that the variation of friction force as a function of loads shows nonmonotonic trend, and it is consistent with the case of Au substrate that we already have discussed before (see Figure 6). Notably, this behaviour is consistently observed for all sliding velocities, indicating that the nonmonotonic features are largely independent of velocity. Overall, among all systems, the Ag/MoS_2_/Si system exhibits the highest friction force across all velocities, followed by Ag/WSe_2_/Si and Ag/MoSe_2_/Si, while the Ag/WS_2_/Si system shows the lowest friction force over the entire velocity range. In contrast, when considering the Au substrate, the Au/WSe_2_/Si system demonstrates the highest friction force at all velocities, followed by Au/MoS_2_/Si and Au/WS_2_/Si, as discussed in above paragraph. This indicates that the friction force can change significantly depending on the substrate. Furthermore, the calculated μ (as shown in Supporting Information File 1, Section II, Figure S6, S7, S8 and S9) show quite large errors, arising from the nonmonotonic dependence of the friction force on the applied load. We already observed a similar trend in the case of the Au substrate, suggesting that the overall nature of frictional behaviour in our systems is largely independent of the substrate. Similarly to the case of the Au substrate, we fit the COF at variable load with tip velocity of 2 m/s by using Equation 4; results are reported in the section “Frictional properties” of Supporting Information File 1. Among all Ag substrate systems, Ag/MoS_2_/Si exhibits the largest value of *A*, indicating significantly stronger adhesive interactions at the interface; this enhanced adhesion contributes directly to the larger friction force observed for this system. Furthermore, the values of *A* obtained for Ag/MoS_2_/Si are consistently higher than those for Au/MoS_2_/Si across all sliding velocities, in agreement with the corresponding friction-force trends reported in Figure 13. In contrast, for the WSe_2_-based systems, Au/WSe_2_/Si exhibits a larger *A* value than Ag/WSe_2_/Si, which likewise correlates with its higher friction force. Overall, the fitting parameter *A* shows a strong positive correlation with the friction force. Systems characterised by larger *A* values generally exhibit higher friction forces, highlighting the dominant role of interfacial adhesion in governing the frictional response of these heterostructures.

**Figure 14:**
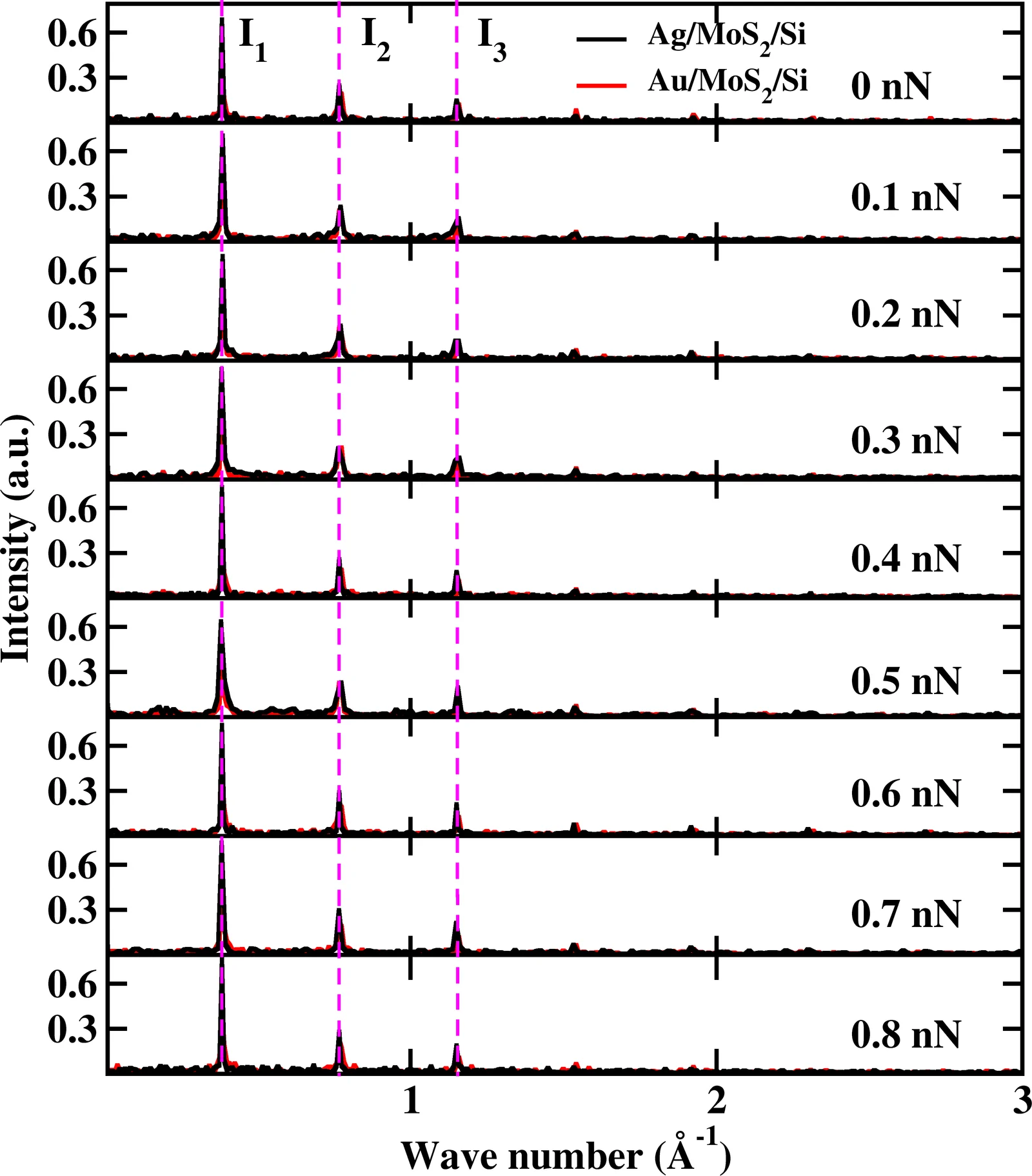
Fourier transform of the force on a tip varying with loads and different substrate for a velocity of 2 m/s.

**Figure 15:**
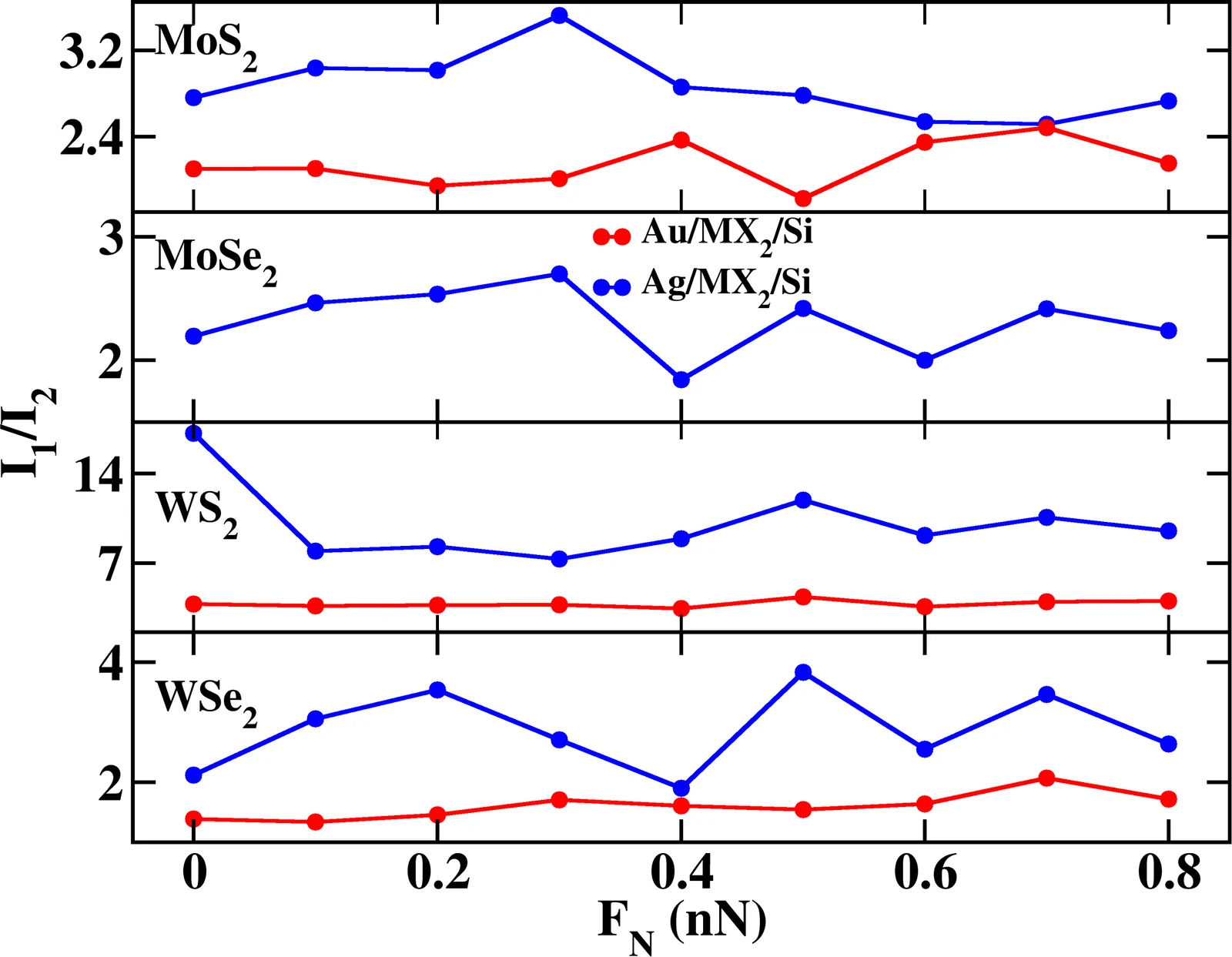
Ratio of the peak intensity *I*_1_ and *I*_2_ varying with loads and different substrate for a tip velocity of 2 m/s. The Au/MoSe_2_/Si system has not been considered because the *I*_2_ intensity is null.

Figure 14 shows the three sharp peaks in FT at a velocity of 2 m/s for both Ag/MoS_2_/Si and Au/MoS_2_/Si systems, which have very similar profiles. We find that the positions of all three peaks remain nearly unchanged for different applied loads and different substrates, indicating that they are independent of loads and substrates. We also observe that the peak positions in the Fourier transforms shift as the monolayer material is varied (Supporting Information File 1, Section II, Figure S10), confirming that the forces experienced by the tip depend on the specific monolayer. In particular, the first peak intensities at varying loads are significantly higher in the Ag/MX_2_/Si systems compared to the Au/MX_2_/Si systems; this indicates that the friction contribution along the sliding direction is greater when using the Ag substrate (Figure 13). In contrast, the opposite trend is observed with the Au/WSe_2_/Si system, where the friction force is higher compared with the Ag/WSe_2_/Si (Figure 13). This behaviour correlates with the Fourier transform results (which is shown in Supporting Information File 1, Section II, Figure S10d), where the first peak intensity is higher (see Figure 15) in systems with the Au substrate. Overall, these findings suggest that the contribution of multiple sliding and lateral motion in the friction profile strongly influences the average friction force, depending on the specific monolayer and substrate combination.

Focusing to the intensity ratio of both Au/MX_2_/Si and Ag/MX_2_/Si system (see Figure 15), the variation of the peak-intensity ratio follows a trend similar to that of the friction force (see Figure 13). In particular, the peak intensity ratios are higher in the case of the Ag substrate compared with the Au substrate, regardless of the applied loads. Furthermore, the peak-intensity ratio exhibits a clear nonmonotonic dependence on the applied load, consistent with the nonmonotonic behaviour observed in the friction force, resulting in high errors in the estimation of the friction coefficient from a linear fit against the applied load. Therefore, the lateral motion of the tip plays an important role in determining the overall friction force and contributes to both the reduction of the average friction force and the emergence of its nonmonotonic behaviour.

## Conclusion

In this study, we investigate the nanoscale friction behaviour of TM/MX_2_/Si systems (TM = Au, Ag; M = Mo, W; X = S, Se) using classical molecular dynamics simulations with machine-learning-based force fields trained on DFT data. Our results show that the mean friction force does not follow a strictly linear dependence on the applied normal load. Instead, a nonmonotonic friction–load relationship is observed, which introduces significant uncertainty in determining the coefficient of friction. Fitting the calculated COF, μ′, at each applied load as a function of the inverse normal load results in a substantial reduction of the fitting errors and more reliable fitting parameters than those obtained from a linear fit of the friction force versus applied load. Analysis of the spatial Fourier transform of lateral force signals reveals multiple spectral peaks, with their intensities strongly correlated with the average friction force. The presence of these peaks indicates that the tip motion is not limited to the primary sliding direction but also includes significant lateral contributions. Furthermore, the qualitative features of both the friction force profiles and the Fourier spectra remain similar across different substrates. While the magnitude of friction depends on the specific substrate material, the underlying mechanism responsible for the nonmonotonic behaviour arising from the coupling between longitudinal sliding and lateral motion of the tip remains essentially substrate-independent. The analysis method we propose is based on the Fourier transform of the friction force signal. Although the spectral features will vary with the chemical composition and geometry of the sliding interface, the method can be applied to the study of a wide range of sliding systems beyond those here considered.

## Supporting Information

File 1 Additional data and figures.

## Data Availability

Data generated and analyzed during this study is available from the corresponding author upon reasonable request.

## References

[1] Sarkar M, Mandal N (2022). Mater Today: Proc.

[2] Vanossi A, Manini N, Urbakh M, Zapperi S, Tosatti E (2013). Rev Mod Phys.

[3] Zhang S, Ma T, Erdemir A, Li Q (2019). Mater Today.

[4] Zhang D, Li Z, Klausen L H, Li Q, Dong M (2022). Mater Today Phys.

[5] Marian M, Berman D, Rota A, Jackson R L, Rosenkranz A (2022). Adv Mater Interfaces.

[6] Wang J, Khosravi A, Vanossi A, Tosatti E (2024). Rev Mod Phys.

[7] Cammarata A, Perviz E, Polcar T (2024). Prog Surf Sci.

[8] Martin J M, Donnet C, Le Mogne T, Epicier T (1993). Phys Rev B.

[9] Mukhtar S H, Gulzar A, Saleem S, Wani M F, Sehgal R, Yakovenko A A, Goryacheva I G, Sharma M D (2024). Tribol Int.

[10] Schwarz U D, Allers W, Gensterblum G, Wiesendanger R (1995). Phys Rev B.

[11] Smolyanitsky A, Killgore J P (2012). Phys Rev B.

[12] Mandelli D, Ouyang W, Hod O, Urbakh M (2019). Phys Rev Lett.

[13] Prandtl L (1928). Z Angew Math Mech.

[14] Tomlinson G A (1929). London, Edinburgh Dublin Philos Mag J Sci.

[15] Cammarata A, Nicolini P, Simonovic K, Ukraintsev E, Polcar T (2019). Phys Rev B.

[16] Bai H, Zou G, Bao H, Li S, Ma F, Gao H (2023). ACS Nano.

[17] Wei Z, Duan Z, Kan Y, Zhang Y, Chen Y (2020). J Appl Phys.

[18] Lee C, Li Q, Kalb W, Liu X-Z, Berger H, Carpick R W, Hone J (2010). Science.

[19] Li Y, Wu B, Ouyang W, Liu Z, Wang W (2024). Nano Lett.

[20] Fang L, Liu D-M, Guo Y, Liao Z-M, Luo J-B, Wen S-Z (2017). Nanotechnology.

[21] Hao Y, Sun T-Y, Ye J-T, Huang L-F, Wang L-P (2024). Adv Mater (Weinheim, Ger).

[22] Levita G, Righi M C (2017). ChemPhysChem.

[23] Zilibotti G, Corni S, Righi M C (2013). Phys Rev Lett.

[24] Vazirisereshk M R, Hasz K, Zhao M-Q, Johnson A T C, Carpick R W, Martini A (2020). ACS Nano.

[25] Yao Y, Song Y, Wu B, Scherb S, Huang S, Hinaut A, Glatzel T, Meyer E, Liu Z, Ouyang W (2025). Adv Sci.

[26] Gao X, Urbakh M, Hod O (2022). Phys Rev Lett.

[27] Shi Y, Cai Z, Pu J, Wang L, Xue Q (2019). Ceram Int.

[28] Ostadhossein A, Rahnamoun A, Wang Y, Zhao P, Zhang S, Crespi V H, van Duin A C T (2017). J Phys Chem Lett.

[29] Brenner D W, Shenderova O A, Harrison J A, Stuart S J, Ni B, Sinnott S B (2002). J Phys: Condens Matter.

[30] Koczanowski P, Nicolini P, Khaksar H, Gnecco E (2025). Small.

[31] Agrawal A, Choudhary A (2016). APL Mater.

[32] Yao K, Herr J E, Toth D W, Mckintyre R, Parkhill J (2018). Chem Sci.

[33] Rizzo T (2021). Phys Rev B.

[34] Bartók A P, Csányi G (2015). Int J Quantum Chem.

[35] Behler J, Parrinello M (2007). Phys Rev Lett.

[36] Shaidu Y, Naik M H, Louie S G, Neaton J B (2025). npj Comput Mater.

[37] Lu D, Wang H, Chen M, Lin L, Car R, E W, Jia W, Zhang L (2021). Comput Phys Commun.

[38] Li R, Liu Z, Rohskopf A, Gordiz K, Henry A, Lee E, Luo T (2020). Appl Phys Lett.

[39] Calegari Andrade M F, Selloni A (2020). Phys Rev Mater.

[40] Liu X, Wang B, Jia K, Wang Q, Wang D, Xiong Y (2024). J Appl Phys.

[41] Zhou J, Fu Y, Liu L, Liu C (2025). J Phys Chem C.

[42] Kresse G, Furthmüller J (1996). Phys Rev B.

[43] Blöchl P E (1994). Phys Rev B.

[44] Perdew J P, Burke K, Ernzerhof M (1996). Phys Rev Lett.

[45] Monkhorst H J, Pack J D (1976). Phys Rev B.

[46] Grimme S, Antony J, Ehrlich S, Krieg H (2010). J Chem Phys.

[47] Zhang L, Han J, Wang H, Car R, E W (2018). Phys Rev Lett.

[48] Wang X, Wang Y, Zhang L, Dai F, Wang H (2022). Nucl Fusion.

[49] Zhang Y, Wang H, Chen W, Zeng J, Zhang L, Wang H, E W (2020). Comput Phys Commun.

[50] Thompson A P, Aktulga H M, Berger R, Bolintineanu D S, Brown W M, Crozier P S, in 't Veld P J, Kohlmeyer A, Moore S G, Nguyen T D (2022). Comput Phys Commun.

[51] Suh I-K, Ohta H, Waseda Y (1988). J Mater Sci.

[52] Bartók A P, Payne M C, Kondor R, Csányi G (2010). Phys Rev Lett.

[53] Wan S, Tong R, Han B, Zhang H (2025). Comput Mater Sci.

[54] Hajinazar S, Thorn A, Sandoval E D, Kharabadze S, Kolmogorov A N (2021). Comput Phys Commun.

[55] Vazirisereshk M R, Ye H, Ye Z, Otero-de-la-Roza A, Zhao M-Q, Gao Z, Johnson A T C, Johnson E R, Carpick R W, Martini A (2019). Nano Lett.

[56] So M, Hong S-H, Kang S-W (2025). Mater Res Express.

[57] Claerbout V E P, Polcar T, Nicolini P (2019). Comput Mater Sci.

[58] Wei Y, Ru G, Qi W, Tang K, Xue T (2022). Front Mech Eng.

[59] Lee H G, Yoon H M, Lee J S (2019). Appl Surf Sci.

[60] Chen R, Wang S, Jia X, Jiang W, Liu Z, Ouyang W, Gao E (2026). ACS Appl Mater Interfaces.

[61] Jung G S, Wang S, Qin Z, Martin-Martinez F J, Warner J H, Buehler M J (2018). ACS Nano.

[62] Song Y, Wang J, Hinaut A, Scherb S, Huang S, Glatzel T, Tosatti E, Meyer E (2024). Phys Rev Lett.

[63] Carpick R W, Salmeron M (1997). Chem Rev.

[64] Gao J, Luedtke W D, Gourdon D, Ruths M, Israelachvili J N, Landman U (2004). J Phys Chem B.

[65] Sheehan P E, Lieber C M (1996). Science.

[66] Momma K, Izumi F (2008). J Appl Crystallogr.

