# Supplementary material for: Atom-scale contributions to nanofriction response in transition metal dichalcogenides
Source: Beilstein J Nanotechnol. 2026 Oct 2;17:1330–47. doi: 10.3762/bjnano.17.92 (PMC13635904; doi:10.3762/bjnano.17.92)
Supplement: Supplementary file 1 [file Beilstein_J_Nanotechnol-17-1330-s001.pdf]

BEILSTEIN JOURNAL OF NANOTECHNOLOGY

Supporting Information

for

Atom-scale contributions to nanofriction response in transition metal dichalcogenides

Suresh Ravisankar, Ravikant Kumar, Antonio Cammarata, Thilo Glatzel and Tomas Polcar

Beilstein J. Nanotechnol. **2026,** *17,* 1330–1347. doi:10.3762/bjnano.17.92

Additional data and figures

License and Terms: This is a supporting information file under the terms of the Creative Commons Attribution License (https://creativecommons.org/licenses/by/4.0). Please note that the reuse, redistribution and reproduction in particular requires that the author(s) and source are credited and that individual graphics may be subject to special legal provisions.
The license is subject to the *Beilstein Journal of Nanotechnology* terms and conditions: (https://www.beilstein-journals.org/bjnano/terms)

Contents

Structural Information **S2**

Au/MoS_2/Si S10

Au/$MoSe_2$/Si S18

Au/WS2/Si S27

Au/WSe2/Si S35

Ag/MoS2/Si S43

Ag/MoSe2/Si S51

Ag/WS2/Si S59

Ag/WSe2/Si S68

Frictional Properties **S68**

Structural Information

The geometry of each hetrostructure used in the molecular dynamics simulations with a total number of 360 atoms (144 TM, 36 M, 72 X, 108 Si) is provided in what follows in POSCAR format.

```
Au/MoS2/Si
1.0
       15.5672988892         0.0000000000         0.0000000000
        0.0000000000        17.9267997742         0.0000000000
        0.0000000000         0.0000000000        50.0000000000
   Au    Mo     S    Si
  144    36    72   108
Cartesian
     3.904829471        1.172879994        16.812799871
     3.904829471        7.148479830        16.812799871
     3.904829471       13.124079933        16.812799871
     9.093929255        1.172879994        16.812799871
     9.093929255        7.148479830        16.812799871
     9.093929255       13.124079933        16.812799871
    14.283028576        1.172879994        16.812799871
    14.283028576        7.148479830        16.812799871
    14.283028576       13.124079933        16.812799871
     3.894879778        1.172879994        23.676000535
```

3.894879778	7.148479830	23.676000535
3.894879778	13.124079933	23.676000535
9.083979562	1.172879994	23.676000535
9.083979562	7.148479830	23.676000535
9.083979562	13.124079933	23.676000535
14.273078883	1.172879994	23.676000535
14.273078883	7.148479830	23.676000535
14.273078883	13.124079933	23.676000535
3.904829471	4.160680046	16.812799871
3.904829471	10.136279614	16.812799871
3.904829471	16.111880252	16.812799871
9.093929255	4.160680046	16.812799871
9.093929255	10.136279614	16.812799871
9.093929255	16.111880252	16.812799871
14.283028576	4.160680046	16.812799871
14.283028576	10.136279614	16.812799871
14.283028576	16.111880252	16.812799871
3.894879778	4.160680046	23.676000535
3.894879778	10.136279614	23.676000535
3.894879778	16.111880252	23.676000535
9.083979562	4.160680046	23.676000535
9.083979562	10.136279614	23.676000535
9.083979562	16.111880252	23.676000535
14.273078883	4.160680046	23.676000535
14.273078883	10.136279614	23.676000535
14.273078883	16.111880252	23.676000535
0.441922960	1.172879994	19.131700695
0.441922960	7.148479830	19.131700695
0.441922960	13.124079933	19.131700695
5.631022773	1.172879994	19.131700695
5.631022773	7.148479830	19.131700695
5.631022773	13.124079933	19.131700695
10.820122558	1.172879994	19.131700695
10.820122558	7.148479830	19.131700695
10.820122558	13.124079933	19.131700695
0.441922960	4.160680046	19.131700695
0.441922960	10.136279614	19.131700695
0.441922960	16.111880252	19.131700695
5.631022773	4.160680046	19.131700695
5.631022773	10.136279614	19.131700695
5.631022773	16.111880252	19.131700695
10.820122558	4.160680046	19.131700695
10.820122558	10.136279614	19.131700695
10.820122558	16.111880252	19.131700695
2.168479790	1.172879994	21.399800479

2.168479790	7.148479830	21.399800479
2.168479790	13.124079933	21.399800479
7.357579342	1.172879994	21.399800479
7.357579342	7.148479830	21.399800479
7.357579342	13.124079933	21.399800479
12.546678663	1.172879994	21.399800479
12.546678663	7.148479830	21.399800479
12.546678663	13.124079933	21.399800479
2.168479790	4.160680046	21.399800479
2.168479790	10.136279614	21.399800479
2.168479790	16.111880252	21.399800479
7.357579342	4.160680046	21.399800479
7.357579342	10.136279614	21.399800479
7.357579342	16.111880252	21.399800479
12.546678663	4.160680046	21.399800479
12.546678663	10.136279614	21.399800479
12.546678663	16.111880252	21.399800479
3.036469634	2.666779887	19.131700695
3.036469634	8.642379989	19.131700695
3.036469634	14.617979558	19.131700695
8.225569650	2.666779887	19.131700695
8.225569650	8.642379989	19.131700695
8.225569650	14.617979558	19.131700695
13.414668970	2.666779887	19.131700695
13.414668970	8.642379989	19.131700695
13.414668970	14.617979558	19.131700695
3.036469634	5.654580205	19.131700695
3.036469634	11.630180308	19.131700695
3.036469634	17.605779877	19.131700695
8.225569650	5.654580205	19.131700695
8.225569650	11.630180308	19.131700695
8.225569650	17.605779877	19.131700695
13.414668970	5.654580205	19.131700695
13.414668970	11.630180308	19.131700695
13.414668970	17.605779877	19.131700695
15.141231570	2.666779887	21.399800479
15.141231570	8.642379989	21.399800479
15.141231570	14.617979558	21.399800479
4.763032466	2.666779887	21.399800479
4.763032466	8.642379989	21.399800479
4.763032466	14.617979558	21.399800479
9.952132250	2.666779887	21.399800479
9.952132250	8.642379989	21.399800479
9.952132250	14.617979558	21.399800479
15.141231570	5.654580205	21.399800479

15.141231570	11.630180308	21.399800479
15.141231570	17.605779877	21.399800479
4.763032466	5.654580205	21.399800479
4.763032466	11.630180308	21.399800479
4.763032466	17.605779877	21.399800479
9.952132250	5.654580205	21.399800479
9.952132250	11.630180308	21.399800479
9.952132250	17.605779877	21.399800479
1.310279927	2.666779887	16.812799871
1.310279927	8.642379989	16.812799871
1.310279927	14.617979558	16.812799871
6.499379595	2.666779887	16.812799871
6.499379595	8.642379989	16.812799871
6.499379595	14.617979558	16.812799871
11.688479379	2.666779887	16.812799871
11.688479379	8.642379989	16.812799871
11.688479379	14.617979558	16.812799871
1.300330002	2.666779887	23.676000535
1.300330002	8.642379989	23.676000535
1.300330002	14.617979558	23.676000535
6.489429438	2.666779887	23.676000535
6.489429438	8.642379989	23.676000535
6.489429438	14.617979558	23.676000535
11.678529687	2.666779887	23.676000535
11.678529687	8.642379989	23.676000535
11.678529687	14.617979558	23.676000535
1.310279927	5.654580205	16.812799871
1.310279927	11.630180308	16.812799871
1.310279927	17.605779877	16.812799871
6.499379595	5.654580205	16.812799871
6.499379595	11.630180308	16.812799871
6.499379595	17.605779877	16.812799871
11.688479379	5.654580205	16.812799871
11.688479379	11.630180308	16.812799871
11.688479379	17.605779877	16.812799871
1.300330002	5.654580205	23.676000535
1.300330002	11.630180308	23.676000535
1.300330002	17.605779877	23.676000535
6.489429438	5.654580205	23.676000535
6.489429438	11.630180308	23.676000535
6.489429438	17.605779877	23.676000535
11.678529687	5.654580205	23.676000535
11.678529687	11.630180308	23.676000535
11.678529687	17.605779877	23.676000535
15.139013001	2.666749968	27.767300606

15.139013001	8.642350071	27.767300606
15.139013001	14.617949639	27.767300606
4.760813897	2.666749968	27.767300606
4.760813897	8.642350071	27.767300606
4.760813897	14.617949639	27.767300606
9.949913681	2.666749968	27.767300606
9.949913681	8.642350071	27.767300606
9.949913681	14.617949639	27.767300606
15.139013001	5.654549752	27.767300606
15.139013001	11.630149321	27.767300606
15.139013001	17.605749958	27.767300606
4.760813897	5.654549752	27.767300606
4.760813897	11.630149321	27.767300606
4.760813897	17.605749958	27.767300606
9.949913681	5.654549752	27.767300606
9.949913681	11.630149321	27.767300606
9.949913681	17.605749958	27.767300606
2.166259829	1.172900029	27.767300606
2.166259829	7.148500132	27.767300606
2.166259829	13.124100235	27.767300606
7.355359381	1.172900029	27.767300606
7.355359381	7.148500132	27.767300606
7.355359381	13.124100235	27.767300606
12.544459166	1.172900029	27.767300606
12.544459166	7.148500132	27.767300606
12.544459166	13.124100235	27.767300606
2.166259829	4.160700081	27.767300606
2.166259829	10.136299916	27.767300606
2.166259829	16.111899485	27.767300606
7.355359381	4.160700081	27.767300606
7.355359381	10.136299916	27.767300606
7.355359381	16.111899485	27.767300606
12.544459166	4.160700081	27.767300606
12.544459166	10.136299916	27.767300606
12.544459166	16.111899485	27.767300606
1.300399825	2.666869909	29.409000278
1.300399825	8.642469745	29.409000278
1.300399825	14.618069314	29.409000278
6.489499494	2.666869909	29.409000278
6.489499494	8.642469745	29.409000278
6.489499494	14.618069314	29.409000278
11.678599278	2.666869909	29.409000278
11.678599278	8.642469745	29.409000278
11.678599278	14.618069314	29.409000278
1.300399825	5.654669961	29.409000278

1.300399825	11.630270064	29.409000278
1.300399825	17.605869632	29.409000278
6.489499494	5.654669961	29.409000278
6.489499494	11.630270064	29.409000278
6.489499494	17.605869632	29.409000278
11.678599278	5.654669961	29.409000278
11.678599278	11.630270064	29.409000278
11.678599278	17.605869632	29.409000278
3.894949833	1.172789972	29.409000278
3.894949833	7.148390074	29.409000278
3.894949833	13.123990177	29.409000278
9.084049154	1.172789972	29.409000278
9.084049154	7.148390074	29.409000278
9.084049154	13.123990177	29.409000278
14.273149402	1.172789972	29.409000278
14.273149402	7.148390074	29.409000278
14.273149402	13.123990177	29.409000278
3.894949833	4.160590023	29.409000278
3.894949833	10.136189859	29.409000278
3.894949833	16.111789427	29.409000278
9.084049154	4.160590023	29.409000278
9.084049154	10.136189859	29.409000278
9.084049154	16.111789427	29.409000278
14.273149402	4.160590023	29.409000278
14.273149402	10.136189859	29.409000278
14.273149402	16.111789427	29.409000278
1.300379876	2.666790038	26.137998700
1.300379876	8.642390140	26.137998700
1.300379876	14.617990243	26.137998700
6.489479544	2.666790038	26.137998700
6.489479544	8.642390140	26.137998700
6.489479544	14.617990243	26.137998700
11.678578864	2.666790038	26.137998700
11.678578864	8.642390140	26.137998700
11.678578864	14.617990243	26.137998700
1.300379876	5.654589822	26.137998700
1.300379876	11.630189925	26.137998700
1.300379876	17.605789493	26.137998700
6.489479544	5.654589822	26.137998700
6.489479544	11.630189925	26.137998700
6.489479544	17.605789493	26.137998700
11.678578864	5.654589822	26.137998700
11.678578864	11.630189925	26.137998700
11.678578864	17.605789493	26.137998700
3.894929884	1.172859960	26.137998700

3.894929884	7.148460063	26.137998700
3.894929884	13.124059631	26.137998700
9.084029668	1.172859960	26.137998700
9.084029668	7.148460063	26.137998700
9.084029668	13.124059631	26.137998700
14.273128989	1.172859960	26.137998700
14.273128989	7.148460063	26.137998700
14.273128989	13.124059631	26.137998700
3.894929884	4.160660011	26.137998700
3.894929884	10.136260381	26.137998700
3.894929884	16.111859950	26.137998700
9.084029668	4.160660011	26.137998700
9.084029668	10.136260381	26.137998700
9.084029668	16.111859950	26.137998700
14.273128989	4.160660011	26.137998700
14.273128989	10.136260381	26.137998700
14.273128989	16.111859950	26.137998700
0.430496947	1.183179998	32.341098785
0.430496947	7.158779834	32.341098785
0.430496947	13.134379402	32.341098785
5.619596354	1.183179998	32.341098785
5.619596354	7.158779834	32.341098785
5.619596354	13.134379402	32.341098785
10.808696603	1.183179998	32.341098785
10.808696603	7.158779834	32.341098785
10.808696603	13.134379402	32.341098785
3.025029760	2.656389860	32.341098785
3.025029760	8.631989696	32.341098785
3.025029760	14.607589264	32.341098785
8.214129776	2.656389860	32.341098785
8.214129776	8.631989696	32.341098785
8.214129776	14.607589264	32.341098785
13.403229097	2.656389860	32.341098785
13.403229097	8.631989696	32.341098785
13.403229097	14.607589264	32.341098785
0.430496947	4.170969899	32.341098785
0.430496947	10.146569467	32.341098785
0.430496947	16.122170104	32.341098785
5.619596354	4.170969899	32.341098785
5.619596354	10.146569467	32.341098785
5.619596354	16.122170104	32.341098785
10.808696603	4.170969899	32.341098785
10.808696603	10.146569467	32.341098785
10.808696603	16.122170104	32.341098785
3.025029760	5.644179761	32.341098785

3.025029760	11.619779329	32.341098785
3.025029760	17.595379966	32.341098785
8.214129776	5.644179761	32.341098785
8.214129776	11.619779329	32.341098785
8.214129776	17.595379966	32.341098785
13.403229097	5.644179761	32.341098785
13.403229097	11.619779329	32.341098785
13.403229097	17.595379966	32.341098785
1.295339845	5.657970087	34.130498767
1.295339845	11.633569655	34.130498767
1.295339845	17.609170292	34.130498767
6.484439282	5.657970087	34.130498767
6.484439282	11.633569655	34.130498767
6.484439282	17.609170292	34.130498767
11.673539530	5.657970087	34.130498767
11.673539530	11.633569655	34.130498767
11.673539530	17.609170292	34.130498767
3.889879647	1.155600014	34.130498767
3.889879647	7.131199716	34.130498767
3.889879647	13.106799819	34.130498767
9.078979199	1.155600014	34.130498767
9.078979199	7.131199716	34.130498767
9.078979199	13.106799819	34.130498767
14.268078520	1.155600014	34.130498767
14.268078520	7.131199716	34.130498767
14.268078520	13.106799819	34.130498767
1.295339845	2.670179919	34.130600095
1.295339845	8.645780022	34.130600095
1.295339845	14.621379590	34.130600095
6.484439282	2.670179919	34.130600095
6.484439282	8.645780022	34.130600095
6.484439282	14.621379590	34.130600095
11.673539530	2.670179919	34.130600095
11.673539530	8.645780022	34.130600095
11.673539530	14.621379590	34.130600095
3.889879647	4.143399932	34.130498767
3.889879647	10.118999500	34.130498767
3.889879647	16.094600137	34.130498767
9.078979199	4.143399932	34.130498767
9.078979199	10.118999500	34.130498767
9.078979199	16.094600137	34.130498767
14.268078520	4.143399932	34.130498767
14.268078520	10.118999500	34.130498767
14.268078520	16.094600137	34.130498767
1.295339845	5.657970087	36.510300636

```
     1.295339845        11.633569655        36.510300636
     1.295339845        17.609170292        36.510300636
     6.484439282         5.657970087        36.510300636
     6.484439282        11.633569655        36.510300636
     6.484439282        17.609170292        36.510300636
    11.673539530         5.657970087        36.510300636
    11.673539530        11.633569655        36.510300636
    11.673539530        17.609170292        36.510300636
     3.889879647         1.155600014        36.510199308
     3.889879647         7.131199716        36.510199308
     3.889879647        13.106799819        36.510199308
     9.078979199         1.155600014        36.510199308
     9.078979199         7.131199716        36.510199308
     9.078979199        13.106799819        36.510199308
    14.268078520         1.155600014        36.510199308
    14.268078520         7.131199716        36.510199308
    14.268078520        13.106799819        36.510199308
     1.295339845         2.670179919        36.510300636
     1.295339845         8.645780022        36.510300636
     1.295339845        14.621379590        36.510300636
     6.484439282         2.670179919        36.510300636
     6.484439282         8.645780022        36.510300636
     6.484439282        14.621379590        36.510300636
    11.673539530         2.670179919        36.510300636
    11.673539530         8.645780022        36.510300636
    11.673539530        14.621379590        36.510300636
     3.889879647         4.143399932        36.510199308
     3.889879647        10.118999500        36.510199308
     3.889879647        16.094600137        36.510199308
     9.078979199         4.143399932        36.510199308
     9.078979199        10.118999500        36.510199308
     9.078979199        16.094600137        36.510199308
    14.268078520         4.143399932        36.510199308
    14.268078520        10.118999500        36.510199308
    14.268078520        16.094600137        36.510199308

Au/MoSe2/Si
1.0
      15.9442605972         0.0000000000         0.0000000000
       0.0000000000        18.3540554047         0.0000000000
       0.0000000000         0.0000000000        50.0000000000
   Au   Mo   Se   Si
  144   36   72  108
Cartesian
     0.000000000         0.000000000        20.000000298
```

0.000000000	6.118018651	20.000000298
0.000000000	12.236037301	20.000000298
5.314753691	0.000000000	20.000000298
5.314753691	6.118018651	20.000000298
5.314753691	12.236037301	20.000000298
10.629507382	0.000000000	20.000000298
10.629507382	6.118018651	20.000000298
10.629507382	12.236037301	20.000000298
5.185026843	6.001260705	26.669687033
5.185026843	12.119279356	26.669687033
5.185026843	18.237297459	26.669687033
10.499780534	6.001260705	26.669687033
10.499780534	12.119279356	26.669687033
10.499780534	18.237297459	26.669687033
15.814533749	6.001260705	26.669687033
15.814533749	12.119279356	26.669687033
15.814533749	18.237297459	26.669687033
0.000000000	3.059009325	20.000000298
0.000000000	9.177027702	20.000000298
0.000000000	15.295045806	20.000000298
5.314753691	3.059009325	20.000000298
5.314753691	9.177027702	20.000000298
5.314753691	15.295045806	20.000000298
10.629507382	3.059009325	20.000000298
10.629507382	9.177027702	20.000000298
10.629507382	15.295045806	20.000000298
5.185026843	2.942251380	26.669687033
5.185026843	9.060269757	26.669687033
5.185026843	15.178287860	26.669687033
10.499780534	2.942251380	26.669687033
10.499780534	9.060269757	26.669687033
10.499780534	15.178287860	26.669687033
15.814533749	2.942251380	26.669687033
15.814533749	9.060269757	26.669687033
15.814533749	15.178287860	26.669687033
1.726741453	6.070536335	22.249865532
1.726741453	12.188554985	22.249865532
1.726741453	18.306573089	22.249865532
7.041494787	6.070536335	22.249865532
7.041494787	12.188554985	22.249865532
7.041494787	18.306573089	22.249865532
12.356248478	6.070536335	22.249865532
12.356248478	12.188554985	22.249865532
12.356248478	18.306573089	22.249865532
1.726741453	3.011527557	22.249865532

1.726741453	9.129545934	22.249865532
1.726741453	15.247564584	22.249865532
7.041494787	3.011527557	22.249865532
7.041494787	9.129545934	22.249865532
7.041494787	15.247564584	22.249865532
12.356248478	3.011527557	22.249865532
12.356248478	9.129545934	22.249865532
12.356248478	15.247564584	22.249865532
3.452869453	6.037171921	24.451269209
3.452869453	12.155190571	24.451269209
3.452869453	18.273208675	24.451269209
8.767623381	6.037171921	24.451269209
8.767623381	12.155190571	24.451269209
8.767623381	18.273208675	24.451269209
14.082376597	6.037171921	24.451269209
14.082376597	12.155190571	24.451269209
14.082376597	18.273208675	24.451269209
3.452869453	2.978162595	24.451269209
3.452869453	9.096180973	24.451269209
3.452869453	15.214199076	24.451269209
8.767623381	2.978162595	24.451269209
8.767623381	9.096180973	24.451269209
8.767623381	15.214199076	24.451269209
14.082376597	2.978162595	24.451269209
14.082376597	9.096180973	24.451269209
14.082376597	15.214199076	24.451269209
4.386159060	1.483371096	22.249738872
4.386159060	7.601389473	22.249738872
4.386159060	13.719408124	22.249738872
9.700912751	1.483371096	22.249738872
9.700912751	7.601389473	22.249738872
9.700912751	13.719408124	22.249738872
15.015665967	1.483371096	22.249738872
15.015665967	7.601389473	22.249738872
15.015665967	13.719408124	22.249738872
4.386159060	4.542380695	22.249738872
4.386159060	10.660399619	22.249738872
4.386159060	16.778417722	22.249738872
9.700912751	4.542380695	22.249738872
9.700912751	10.660399619	22.249738872
9.700912751	16.778417722	22.249738872
15.015665967	4.542380695	22.249738872
15.015665967	10.660399619	22.249738872
15.015665967	16.778417722	22.249738872
0.796382790	1.444921420	24.454124272

0.796382790	7.562939661	24.454124272
0.796382790	13.680958858	24.454124272
6.111136303	1.444921420	24.454124272
6.111136303	7.562939661	24.454124272
6.111136303	13.680958858	24.454124272
11.425889994	1.444921420	24.454124272
11.425889994	7.562939661	24.454124272
11.425889994	13.680958858	24.454124272
0.796382790	4.503931156	24.454124272
0.796382790	10.621949259	24.454124272
0.796382790	16.739968457	24.454124272
6.111136303	4.503931156	24.454124272
6.111136303	10.621949259	24.454124272
6.111136303	16.739968457	24.454124272
11.425889994	4.503931156	24.454124272
11.425889994	10.621949259	24.454124272
11.425889994	16.739968457	24.454124272
2.657376845	1.529504663	20.000000298
2.657376845	7.647522903	20.000000298
2.657376845	13.765541553	20.000000298
7.972130299	1.529504663	20.000000298
7.972130299	7.647522903	20.000000298
7.972130299	13.765541553	20.000000298
13.286883514	1.529504663	20.000000298
13.286883514	7.647522903	20.000000298
13.286883514	13.765541553	20.000000298
2.531148718	1.412257705	26.669946313
2.531148718	7.530275945	26.669946313
2.531148718	13.648294596	26.669946313
7.845902172	1.412257705	26.669946313
7.845902172	7.530275945	26.669946313
7.845902172	13.648294596	26.669946313
13.160655862	1.412257705	26.669946313
13.160655862	7.530275945	26.669946313
13.160655862	13.648294596	26.669946313
2.657376845	4.588514398	20.000000298
2.657376845	10.706533049	20.000000298
2.657376845	16.824551152	20.000000298
7.972130299	4.588514398	20.000000298
7.972130299	10.706533049	20.000000298
7.972130299	16.824551152	20.000000298
13.286883514	4.588514398	20.000000298
13.286883514	10.706533049	20.000000298
13.286883514	16.824551152	20.000000298
2.531148718	4.471267167	26.669946313

2.531148718	10.589286091	26.669946313
2.531148718	16.707304195	26.669946313
7.845902172	4.471267167	26.669946313
7.845902172	10.589286091	26.669946313
7.845902172	16.707304195	26.669946313
13.160655862	4.471267167	26.669946313
13.160655862	10.589286091	26.669946313
13.160655862	16.707304195	26.669946313
0.720179648	1.334453490	31.018465757
0.720179648	7.452472140	31.018465757
0.720179648	13.570490244	31.018465757
6.034933279	1.334453490	31.018465757
6.034933279	7.452472140	31.018465757
6.034933279	13.570490244	31.018465757
11.349686970	1.334453490	31.018465757
11.349686970	7.452472140	31.018465757
11.349686970	13.570490244	31.018465757
0.720179648	4.393462815	31.018465757
0.720179648	10.511481739	31.018465757
0.720179648	16.629499843	31.018465757
6.034933279	4.393462815	31.018465757
6.034933279	10.511481739	31.018465757
6.034933279	16.629499843	31.018465757
11.349686970	4.393462815	31.018465757
11.349686970	10.511481739	31.018465757
11.349686970	16.629499843	31.018465757
3.377824671	5.922980469	31.018307805
3.377824671	12.040999119	31.018307805
3.377824671	18.159017223	31.018307805
8.692577886	5.922980469	31.018307805
8.692577886	12.040999119	31.018307805
8.692577886	18.159017223	31.018307805
14.007332052	5.922980469	31.018307805
14.007332052	12.040999119	31.018307805
14.007332052	18.159017223	31.018307805
3.377824671	2.863971417	31.018307805
3.377824671	8.981990067	31.018307805
3.377824671	15.100008718	31.018307805
8.692577886	2.863971417	31.018307805
8.692577886	8.981990067	31.018307805
8.692577886	15.100008718	31.018307805
14.007332052	2.863971417	31.018307805
14.007332052	8.981990067	31.018307805
14.007332052	15.100008718	31.018307805
2.489290940	1.332780647	32.792428136

2.489290940	7.450798887	32.792428136
2.489290940	13.568817538	32.792428136
7.804044393	1.332780647	32.792428136
7.804044393	7.450798887	32.792428136
7.804044393	13.568817538	32.792428136
13.118797609	1.332780647	32.792428136
13.118797609	7.450798887	32.792428136
13.118797609	13.568817538	32.792428136
2.489290940	4.391790109	32.792428136
2.489290940	10.509809033	32.792428136
2.489290940	16.627827136	32.792428136
7.804044393	4.391790109	32.792428136
7.804044393	10.509809033	32.792428136
7.804044393	16.627827136	32.792428136
13.118797609	4.391790109	32.792428136
13.118797609	10.509809033	32.792428136
13.118797609	16.627827136	32.792428136
5.146511928	5.921592199	32.792788744
5.146511928	12.039610850	32.792788744
5.146511928	18.157628953	32.792788744
10.461265619	5.921592199	32.792788744
10.461265619	12.039610850	32.792788744
10.461265619	18.157628953	32.792788744
15.776018834	5.921592199	32.792788744
15.776018834	12.039610850	32.792788744
15.776018834	18.157628953	32.792788744
5.146511928	2.862582874	32.792788744
5.146511928	8.980601251	32.792788744
5.146511928	15.098619355	32.792788744
10.461265619	2.862582874	32.792788744
10.461265619	8.980601251	32.792788744
10.461265619	15.098619355	32.792788744
15.776018834	2.862582874	32.792788744
15.776018834	8.980601251	32.792788744
15.776018834	15.098619355	32.792788744
2.493253195	1.336985386	29.265865684
2.493253195	7.455003626	29.265865684
2.493253195	13.573022824	29.265865684
7.808006886	1.336985386	29.265865684
7.808006886	7.455003626	29.265865684
7.808006886	13.573022824	29.265865684
13.122760577	1.336985386	29.265865684
13.122760577	7.455003626	29.265865684
13.122760577	13.573022824	29.265865684
2.493253195	4.395994848	29.265865684

2.493253195	10.514013225	29.265865684
2.493253195	16.632031328	29.265865684
7.808006886	4.395994848	29.265865684
7.808006886	10.514013225	29.265865684
7.808006886	16.632031328	29.265865684
13.122760577	4.395994848	29.265865684
13.122760577	10.514013225	29.265865684
13.122760577	16.632031328	29.265865684
5.150610796	5.925518518	29.265716672
5.150610796	12.043537169	29.265716672
5.150610796	18.161555273	29.265716672
10.465364487	5.925518518	29.265716672
10.465364487	12.043537169	29.265716672
10.465364487	18.161555273	29.265716672
15.780117702	5.925518518	29.265716672
15.780117702	12.043537169	29.265716672
15.780117702	18.161555273	29.265716672
5.150610796	2.866509740	29.265716672
5.150610796	8.984528117	29.265716672
5.150610796	15.102546768	29.265716672
10.465364487	2.866509740	29.265716672
10.465364487	8.984528117	29.265716672
10.465364487	15.102546768	29.265716672
15.780117702	2.866509740	29.265716672
15.780117702	8.984528117	29.265716672
15.780117702	15.102546768	29.265716672
0.440924002	1.211381598	35.891878605
0.440924002	7.329400249	35.891878605
0.440924002	13.447418900	35.891878605
5.755677574	1.211381598	35.891878605
5.755677574	7.329400249	35.891878605
5.755677574	13.447418900	35.891878605
11.070430790	1.211381598	35.891878605
11.070430790	7.329400249	35.891878605
11.070430790	13.447418900	35.891878605
3.098300729	2.719712554	35.891494155
3.098300729	8.837730931	35.891494155
3.098300729	14.955749035	35.891494155
8.413054657	2.719712554	35.891494155
8.413054657	8.837730931	35.891494155
8.413054657	14.955749035	35.891494155
13.727807873	2.719712554	35.891494155
13.727807873	8.837730931	35.891494155
13.727807873	14.955749035	35.891494155
0.440924002	4.270391197	35.891878605

0.440924002	10.388409301	35.891878605
0.440924002	16.506428498	35.891878605
5.755677574	4.270391197	35.891878605
5.755677574	10.388409301	35.891878605
5.755677574	16.506428498	35.891878605
11.070430790	4.270391197	35.891878605
11.070430790	10.388409301	35.891878605
11.070430790	16.506428498	35.891878605
3.098300729	5.778721879	35.891494155
3.098300729	11.896740530	35.891494155
3.098300729	18.014758633	35.891494155
8.413054657	5.778721879	35.891494155
8.413054657	11.896740530	35.891494155
8.413054657	18.014758633	35.891494155
13.727807873	5.778721879	35.891494155
13.727807873	11.896740530	35.891494155
13.727807873	18.014758633	35.891494155
1.326716324	5.792836499	37.640830874
1.326716324	11.910855149	37.640830874
1.326716324	18.028873253	37.640830874
6.641469777	5.792836499	37.640830874
6.641469777	11.910855149	37.640830874
6.641469777	18.028873253	37.640830874
11.956223468	5.792836499	37.640830874
11.956223468	11.910855149	37.640830874
11.956223468	18.028873253	37.640830874
3.984093169	1.183149898	37.640309334
3.984093169	7.301168275	37.640309334
3.984093169	13.419186378	37.640309334
9.298846385	1.183149898	37.640309334
9.298846385	7.301168275	37.640309334
9.298846385	13.419186378	37.640309334
14.613600551	1.183149898	37.640309334
14.613600551	7.301168275	37.640309334
14.613600551	13.419186378	37.640309334
1.326716324	2.733827994	37.640830874
1.326716324	8.851846645	37.640830874
1.326716324	14.969864748	37.640830874
6.641469777	2.733827994	37.640830874
6.641469777	8.851846645	37.640830874
6.641469777	14.969864748	37.640830874
11.956223468	2.733827994	37.640830874
11.956223468	8.851846645	37.640830874
11.956223468	14.969864748	37.640830874
3.984093169	4.242158949	37.640309334

3.984093169	10.360177874	37.640309334
3.984093169	16.478195977	37.640309334
9.298846385	4.242158949	37.640309334
9.298846385	10.360177874	37.640309334
9.298846385	16.478195977	37.640309334
14.613600551	4.242158949	37.640309334
14.613600551	10.360177874	37.640309334
14.613600551	16.478195977	37.640309334
1.326716324	5.792836499	40.030124784
1.326716324	11.910855149	40.030124784
1.326716324	18.028873253	40.030124784
6.641469777	5.792836499	40.030124784
6.641469777	11.910855149	40.030124784
6.641469777	18.028873253	40.030124784
11.956223468	5.792836499	40.030124784
11.956223468	11.910855149	40.030124784
11.956223468	18.028873253	40.030124784
3.984093169	1.183149898	40.029674768
3.984093169	7.301168275	40.029674768
3.984093169	13.419186378	40.029674768
9.298846385	1.183149898	40.029674768
9.298846385	7.301168275	40.029674768
9.298846385	13.419186378	40.029674768
14.613600551	1.183149898	40.029674768
14.613600551	7.301168275	40.029674768
14.613600551	13.419186378	40.029674768
1.326716324	2.733827994	40.030124784
1.326716324	8.851846645	40.030124784
1.326716324	14.969864748	40.030124784
6.641469777	2.733827994	40.030124784
6.641469777	8.851846645	40.030124784
6.641469777	14.969864748	40.030124784
11.956223468	2.733827994	40.030124784
11.956223468	8.851846645	40.030124784
11.956223468	14.969864748	40.030124784
3.984093169	4.242158949	40.029674768
3.984093169	10.360177874	40.029674768
3.984093169	16.478195977	40.029674768
9.298846385	4.242158949	40.029674768
9.298846385	10.360177874	40.029674768
9.298846385	16.478195977	40.029674768
14.613600551	4.242158949	40.029674768
14.613600551	10.360177874	40.029674768
14.613600551	16.478195977	40.029674768

```
Au/WS2/Si
1.0
      15.6610240936         0.0000000000         0.0000000000
       0.0000000000        17.9489860535         0.0000000000
       0.0000000000         0.0000000000        50.0000000000
   Au    W    S   Si
  144   36   72  108
Cartesian
     0.000000000         0.000000000        20.000000298
     0.000000000         5.982999809        20.000000298
     0.000000000        11.965999618        20.000000298
     5.220340120         0.000000000        20.000000298
     5.220340120         5.982999809        20.000000298
     5.220340120        11.965999618        20.000000298
    10.440699843         0.000000000        20.000000298
    10.440699843         5.982999809        20.000000298
    10.440699843        11.965999618        20.000000298
     5.123150047         5.875819734        26.829200983
     5.123150047        11.858800285        26.829200983
     5.123150047        17.841799559        26.829200983
    10.343500435         5.875819734        26.829200983
    10.343500435        11.858800285        26.829200983
    10.343500435        17.841799559        26.829200983
    15.563800416         5.875819734        26.829200983
    15.563800416        11.858800285        26.829200983
    15.563800416        17.841799559        26.829200983
     0.000000000         2.991499904        20.000000298
     0.000000000         8.974489817        20.000000298
     0.000000000        14.957499790        20.000000298
     5.220340120         2.991499904        20.000000298
     5.220340120         8.974489817        20.000000298
     5.220340120        14.957499790        20.000000298
    10.440699843         2.991499904        20.000000298
    10.440699843         8.974489817        20.000000298
    10.440699843        14.957499790        20.000000298
     5.123150047         2.884329993        26.829200983
     5.123150047         8.867319906        26.829200983
     5.123150047        14.850300457        26.829200983
    10.343500435         2.884329993        26.829200983
    10.343500435         8.867319906        26.829200983
    10.343500435        14.850300457        26.829200983
    15.563800416         2.884329993        26.829200983
    15.563800416         8.867319906        26.829200983
    15.563800416        14.850300457        26.829200983
     1.702759958         5.940040266        22.307200730
```

1.702759958	11.923000490	22.307200730
1.702759958	17.905999764	22.307200730
6.923109879	5.940040266	22.307200730
6.923109879	11.923000490	22.307200730
6.923109879	17.905999764	22.307200730
12.143400058	5.940040266	22.307200730
12.143400058	11.923000490	22.307200730
12.143400058	17.905999764	22.307200730
1.702759958	2.948540094	22.307200730
1.702759958	8.931539903	22.307200730
1.702759958	14.914499592	22.307200730
6.923109879	2.948540094	22.307200730
6.923109879	8.931539903	22.307200730
6.923109879	14.914499592	22.307200730
12.143400058	2.948540094	22.307200730
12.143400058	8.931539903	22.307200730
12.143400058	14.914499592	22.307200730
3.412740071	5.907780222	24.564200640
3.412740071	11.890800358	24.564200640
3.412740071	17.873799631	24.564200640
8.633080424	5.907780222	24.564200640
8.633080424	11.890800358	24.564200640
8.633080424	17.873799631	24.564200640
13.853400008	5.907780222	24.564200640
13.853400008	11.890800358	24.564200640
13.853400008	17.873799631	24.564200640
3.412740071	2.916289946	24.564200640
3.412740071	8.899279859	24.564200640
3.412740071	14.882300529	24.564200640
8.633080424	2.916289946	24.564200640
8.633080424	8.899279859	24.564200640
8.633080424	14.882300529	24.564200640
13.853400008	2.916289946	24.564200640
13.853400008	8.899279859	24.564200640
13.853400008	14.882300529	24.564200640
4.312930018	1.452790008	22.307200730
4.312930018	7.435789817	22.307200730
4.312930018	13.418800324	22.307200730
9.533280406	1.452790008	22.307200730
9.533280406	7.435789817	22.307200730
9.533280406	13.418800324	22.307200730
14.753599989	1.452790008	22.307200730
14.753599989	7.435789817	22.307200730
14.753599989	13.418800324	22.307200730
4.312930018	4.444289912	22.307200730

4.312930018	10.427300152	22.307200730
4.312930018	16.410300496	22.307200730
9.533280406	4.444289912	22.307200730
9.533280406	10.427300152	22.307200730
9.533280406	16.410300496	22.307200730
14.753599989	4.444289912	22.307200730
14.753599989	10.427300152	22.307200730
14.753599989	16.410300496	22.307200730
0.802572986	1.420539994	24.564200640
0.802572986	7.403529773	24.564200640
0.802572986	13.386499626	24.564200640
6.022909898	1.420539994	24.564200640
6.022909898	7.403529773	24.564200640
6.022909898	13.386499626	24.564200640
11.243299958	1.420539994	24.564200640
11.243299958	7.403529773	24.564200640
11.243299958	13.386499626	24.564200640
0.802572986	4.412040032	24.564200640
0.802572986	10.395000524	24.564200640
0.802572986	16.377999798	24.564200640
6.022909898	4.412040032	24.564200640
6.022909898	10.395000524	24.564200640
6.022909898	16.377999798	24.564200640
11.243299958	4.412040032	24.564200640
11.243299958	10.395000524	24.564200640
11.243299958	16.377999798	24.564200640
2.610170060	1.495749952	20.000000298
2.610170060	7.478740266	20.000000298
2.610170060	13.461699956	20.000000298
7.830510180	1.495749952	20.000000298
7.830510180	7.478740266	20.000000298
7.830510180	13.461699956	20.000000298
13.050899774	1.495749952	20.000000298
13.050899774	7.478740266	20.000000298
13.050899774	13.461699956	20.000000298
2.512979987	1.388570011	26.829200983
2.512979987	7.371569820	26.829200983
2.512979987	13.354600119	26.829200983
7.733320107	1.388570011	26.829200983
7.733320107	7.371569820	26.829200983
7.733320107	13.354600119	26.829200983
12.953700366	1.388570011	26.829200983
12.953700366	7.371569820	26.829200983
12.953700366	13.354600119	26.829200983
2.610170060	4.487250258	20.000000298

2.610170060	10.470199784	20.000000298
2.610170060	16.453200128	20.000000298
7.830510180	4.487250258	20.000000298
7.830510180	10.470199784	20.000000298
7.830510180	16.453200128	20.000000298
13.050899774	4.487250258	20.000000298
13.050899774	10.470199784	20.000000298
13.050899774	16.453200128	20.000000298
2.512979987	4.380069915	26.829200983
2.512979987	10.363099947	26.829200983
2.512979987	16.346100291	26.829200983
7.733320107	4.380069915	26.829200983
7.733320107	10.363099947	26.829200983
7.733320107	16.346100291	26.829200983
12.953700366	4.380069915	26.829200983
12.953700366	10.363099947	26.829200983
12.953700366	16.346100291	26.829200983
3.362009956	2.848309986	30.914399028
3.362009956	8.831310062	30.914399028
3.362009956	14.814300242	30.914399028
8.582360344	2.848309986	30.914399028
8.582360344	8.831310062	30.914399028
8.582360344	14.814300242	30.914399028
13.802700464	2.848309986	30.914399028
13.802700464	8.831310062	30.914399028
13.802700464	14.814300242	30.914399028
3.362009956	5.839809890	30.914399028
3.362009956	11.822800071	30.914399028
3.362009956	17.805800414	30.914399028
8.582360344	5.839809890	30.914399028
8.582360344	11.822800071	30.914399028
8.582360344	17.805800414	30.914399028
13.802700464	5.839809890	30.914399028
13.802700464	11.822800071	30.914399028
13.802700464	17.805800414	30.914399028
0.751833012	1.352579960	30.914399028
0.751833012	7.335570140	30.914399028
0.751833012	13.318599904	30.914399028
5.972170214	1.352579960	30.914399028
5.972170214	7.335570140	30.914399028
5.972170214	13.318599904	30.914399028
11.192499599	1.352579960	30.914399028
11.192499599	7.335570140	30.914399028
11.192499599	13.318599904	30.914399028
0.751833012	4.344080131	30.914399028

0.751833012	10.327099732	30.914399028
0.751833012	16.310100076	30.914399028
5.972170214	4.344080131	30.914399028
5.972170214	10.327099732	30.914399028
5.972170214	16.310100076	30.914399028
11.192499599	4.344080131	30.914399028
11.192499599	10.327099732	30.914399028
11.192499599	16.310100076	30.914399028
5.097550105	2.846580050	32.559499145
5.097550105	8.829569963	32.559499145
5.097550105	14.812600262	32.559499145
10.317900026	2.846580050	32.559499145
10.317900026	8.829569963	32.559499145
10.317900026	14.812600262	32.559499145
15.538200007	2.846580050	32.559499145
15.538200007	8.829569963	32.559499145
15.538200007	14.812600262	32.559499145
5.097550105	5.838069791	32.559499145
5.097550105	11.821100090	32.559499145
5.097550105	17.804100434	32.559499145
10.317900026	5.838069791	32.559499145
10.317900026	11.821100090	32.559499145
10.317900026	17.804100434	32.559499145
15.538200007	5.838069791	32.559499145
15.538200007	11.821100090	32.559499145
15.538200007	17.804100434	32.559499145
2.487400115	1.350749993	32.559400797
2.487400115	7.333740173	32.559400797
2.487400115	13.316699863	32.559400797
7.707739768	1.350749993	32.559400797
7.707739768	7.333740173	32.559400797
7.707739768	13.316699863	32.559400797
12.928099957	1.350749993	32.559400797
12.928099957	7.333740173	32.559400797
12.928099957	13.316699863	32.559400797
2.487400115	4.342240002	32.559400797
2.487400115	10.325199691	32.559400797
2.487400115	16.308200035	32.559400797
7.707739768	4.342240002	32.559400797
7.707739768	10.325199691	32.559400797
7.707739768	16.308200035	32.559400797
12.928099957	4.342240002	32.559400797
12.928099957	10.325199691	32.559400797
12.928099957	16.308200035	32.559400797
5.099859976	2.850210027	29.288899899

5.099859976	8.833210103	29.288899899
5.099859976	14.816200284	29.288899899
10.320200096	2.850210027	29.288899899
10.320200096	8.833210103	29.288899899
10.320200096	14.816200284	29.288899899
15.540500077	2.850210027	29.288899899
15.540500077	8.833210103	29.288899899
15.540500077	14.816200284	29.288899899
5.099859976	5.841709931	29.288899899
5.099859976	11.824700112	29.288899899
5.099859976	17.807700455	29.288899899
10.320200096	5.841709931	29.288899899
10.320200096	11.824700112	29.288899899
10.320200096	17.807700455	29.288899899
15.540500077	5.841709931	29.288899899
15.540500077	11.824700112	29.288899899
15.540500077	17.807700455	29.288899899
2.489680115	1.354450045	29.288899899
2.489680115	7.337449854	29.288899899
2.489680115	13.320400450	29.288899899
7.710019768	1.354450045	29.288899899
7.710019768	7.337449854	29.288899899
7.710019768	13.320400450	29.288899899
12.930400027	1.354450045	29.288899899
12.930400027	7.337449854	29.288899899
12.930400027	13.320400450	29.288899899
2.489680115	4.345949949	29.288899899
2.489680115	10.328900278	29.288899899
2.489680115	16.311899552	29.288899899
7.710019768	4.345949949	29.288899899
7.710019768	10.328900278	29.288899899
7.710019768	16.311899552	29.288899899
12.930400027	4.345949949	29.288899899
12.930400027	10.328900278	29.288899899
12.930400027	16.311899552	29.288899899
0.433091005	1.184650057	35.513699055
0.433091005	7.167640103	35.513699055
0.433091005	13.150600328	35.513699055
5.653429958	1.184650057	35.513699055
5.653429958	7.167640103	35.513699055
5.653429958	13.150600328	35.513699055
10.873799949	1.184650057	35.513699055
10.873799949	7.167640103	35.513699055
10.873799949	13.150600328	35.513699055
3.043259898	2.659689989	35.513699055

3.043259898	8.642680170	35.513699055
3.043259898	14.625699771	35.513699055
8.263600018	2.659689989	35.513699055
8.263600018	8.642680170	35.513699055
8.263600018	14.625699771	35.513699055
13.483899998	2.659689989	35.513699055
13.483899998	8.642680170	35.513699055
13.483899998	14.625699771	35.513699055
0.433091005	4.176139932	35.513699055
0.433091005	10.159100156	35.513699055
0.433091005	16.142100500	35.513699055
5.653429958	4.176139932	35.513699055
5.653429958	10.159100156	35.513699055
5.653429958	16.142100500	35.513699055
10.873799949	4.176139932	35.513699055
10.873799949	10.159100156	35.513699055
10.873799949	16.142100500	35.513699055
3.043259898	5.651190161	35.513699055
3.043259898	11.634199599	35.513699055
3.043259898	17.617199943	35.513699055
8.263600018	5.651190161	35.513699055
8.263600018	11.634199599	35.513699055
8.263600018	17.617199943	35.513699055
13.483899998	5.651190161	35.513699055
13.483899998	11.634199599	35.513699055
13.483899998	17.617199943	35.513699055
1.303149947	5.664990065	37.314298749
1.303149947	11.647999503	37.314298749
1.303149947	17.630999847	37.314298749
6.523490067	5.664990065	37.314298749
6.523490067	11.647999503	37.314298749
6.523490067	17.630999847	37.314298749
11.743800316	5.664990065	37.314298749
11.743800316	11.647999503	37.314298749
11.743800316	17.630999847	37.314298749
3.913320007	1.157039952	37.314298749
3.913320007	7.140030132	37.314298749
3.913320007	13.123000520	37.314298749
9.133660127	1.157039952	37.314298749
9.133660127	7.140030132	37.314298749
9.133660127	13.123000520	37.314298749
14.354000247	1.157039952	37.314298749
14.354000247	7.140030132	37.314298749
14.354000247	13.123000520	37.314298749
1.303149947	2.673489894	37.314298749

1.303149947	8.656490237	37.314298749
1.303149947	14.639499675	37.314298749
6.523490067	2.673489894	37.314298749
6.523490067	8.656490237	37.314298749
6.523490067	14.639499675	37.314298749
11.743800316	2.673489894	37.314298749
11.743800316	8.656490237	37.314298749
11.743800316	14.639499675	37.314298749
3.913320007	4.148540123	37.314298749
3.913320007	10.131500348	37.314298749
3.913320007	16.114499622	37.314298749
9.133660127	4.148540123	37.314298749
9.133660127	10.131500348	37.314298749
9.133660127	16.114499622	37.314298749
14.354000247	4.148540123	37.314298749
14.354000247	10.131500348	37.314298749
14.354000247	16.114499622	37.314298749
1.303149947	5.664990065	39.695098996
1.303149947	11.647999503	39.695098996
1.303149947	17.630999847	39.695098996
6.523490067	5.664990065	39.695098996
6.523490067	11.647999503	39.695098996
6.523490067	17.630999847	39.695098996
11.743800316	5.664990065	39.695098996
11.743800316	11.647999503	39.695098996
11.743800316	17.630999847	39.695098996
3.913320007	1.157039952	39.695000648
3.913320007	7.140030132	39.695000648
3.913320007	13.123000520	39.695000648
9.133660127	1.157039952	39.695000648
9.133660127	7.140030132	39.695000648
9.133660127	13.123000520	39.695000648
14.354000247	1.157039952	39.695000648
14.354000247	7.140030132	39.695000648
14.354000247	13.123000520	39.695000648
1.303149947	2.673489894	39.695098996
1.303149947	8.656490237	39.695098996
1.303149947	14.639499675	39.695098996
6.523490067	2.673489894	39.695098996
6.523490067	8.656490237	39.695098996
6.523490067	14.639499675	39.695098996
11.743800316	2.673489894	39.695098996
11.743800316	8.656490237	39.695098996
11.743800316	14.639499675	39.695098996
3.913320007	4.148540123	39.695000648

```
     3.913320007         10.131500348         39.695000648
     3.913320007         16.114499622         39.695000648
     9.133660127          4.148540123         39.695000648
     9.133660127         10.131500348         39.695000648
     9.133660127         16.114499622         39.695000648
    14.354000247          4.148540123         39.695000648
    14.354000247         10.131500348         39.695000648
    14.354000247         16.114499622         39.695000648
```

```
Au/WSe2/Si
1.0
       15.9505681992          0.0000000000          0.0000000000
        0.0000000000         18.4212131500          0.0000000000
        0.0000000000          0.0000000000         50.0000000000
   Au    W   Se   Si
  144   36   72  108
Cartesian
     0.000000000          0.000000000         20.000000298
     0.000000000          6.140400174         20.000000298
     0.000000000         12.280800349         20.000000298
     5.316860028          0.000000000         20.000000298
     5.316860028          6.140400174         20.000000298
     5.316860028         12.280800349         20.000000298
    10.633700090          0.000000000         20.000000298
    10.633700090          6.140400174         20.000000298
    10.633700090         12.280800349         20.000000298
     5.334040157          6.160960035         26.642000675
     5.334070105         12.301499654         26.641801000
     5.334070105          0.020486295         26.641899347
    10.650899710          6.161130223         26.641801000
    10.650799883         12.301399737         26.641899347
    10.650899710          0.020587310         26.641899347
     0.017230993          6.161180182         26.641899347
     0.017230993         12.301299820         26.641899347
     0.017230993          0.020587310         26.641899347
     0.000000000          3.070200087         20.000000298
     0.000000000          9.210609869         20.000000298
     0.000000000         15.350999612         20.000000298
     5.316860028          3.070200087         20.000000298
     5.316860028          9.210609869         20.000000298
     5.316860028         15.350999612         20.000000298
    10.633700090          3.070200087         20.000000298
    10.633700090          9.210609869         20.000000298
    10.633700090         15.350999612         20.000000298
     5.334050140          3.090770104         26.641899347
```

5.334040157	9.231230119	26.641899347
5.334040157	15.371600098	26.641801000
10.650899710	3.090930136	26.641899347
10.650999536	9.231220237	26.641899347
10.650799883	15.371500181	26.641899347
0.017230993	3.090890059	26.641899347
0.017230993	9.231220237	26.641899347
0.017230993	15.371500181	26.641899347
1.778879974	6.148199745	22.235900164
1.778860009	12.288700386	22.235900164
1.778840044	0.007885763	22.236000001
7.095670124	6.148220058	22.236000001
7.095710054	12.288600469	22.235900164
7.095730019	0.007885763	22.235900164
12.412599554	6.148279899	22.235900164
12.412499728	12.288700386	22.235900164
12.412499728	0.007885763	22.236000001
1.778900058	3.078080086	22.236000001
1.778879974	9.218400107	22.235900164
1.778819960	15.358899650	22.235900164
7.095670124	3.078049891	22.235900164
7.095710054	9.218400107	22.236000001
7.095720037	15.358799732	22.235900164
12.412599554	3.078040009	22.235900164
12.412599554	9.218509906	22.235900164
12.412599554	15.358899650	22.236000001
3.556050064	6.153650167	24.433200061
3.556009896	12.294000383	24.433100224
3.556080012	0.013186858	24.433100224
8.872919599	6.153589778	24.433100224
8.872850196	12.294100300	24.433100224
8.872919599	0.013287873	24.433100224
14.189800068	6.153600209	24.433100224
14.189800068	12.294000383	24.433100224
14.189700241	0.013085843	24.433100224
3.556080012	3.083480000	24.433200061
3.556030099	9.223809903	24.433100224
3.556070030	15.364199647	24.433000386
8.872919599	3.083419885	24.433100224
8.872930057	9.223850529	24.433100224
8.872839738	15.364299564	24.433100224
14.189800068	3.083299930	24.433100224
14.189800068	9.223850529	24.433100224
14.189800068	15.364199647	24.433200061
4.437289785	1.542830030	22.235900164

4.437310226	7.683209891	22.236000001
4.437289785	13.823600184	22.235900164
9.754179761	1.542799972	22.235900164
9.754110358	7.683230204	22.235900164
9.754139830	13.823700101	22.235900164
15.071000333	1.542779934	22.235900164
15.071000333	7.683180245	22.235900164
15.070899556	13.823700101	22.235900164
4.437289785	4.613060037	22.236000001
4.437289785	10.753399822	22.235900164
4.437320208	16.893800545	22.235900164
9.754210184	4.612979884	22.235900164
9.754110358	10.753399822	22.235900164
9.754110358	16.893900462	22.235900164
15.071000333	4.612929925	22.235900164
15.071000333	10.753499739	22.235900164
15.071000333	16.893800545	22.235900164
0.897605017	1.548640043	24.433200061
0.897688027	7.688929869	24.433100224
0.897595985	13.829299849	24.433100224
6.214479840	1.548490030	24.433100224
6.214449893	7.688870029	24.433200061
6.214499806	13.829299849	24.433000386
11.531300413	1.548580065	24.433200061
11.531400239	7.689000141	24.433100224
11.531300413	13.829399766	24.433100224
0.897559026	4.618839856	24.433100224
0.897705972	10.759099487	24.433100224
0.897648988	16.899600128	24.433100224
6.214489823	4.618709744	24.433200061
6.214439910	10.759099487	24.433100224
6.214580142	16.899500211	24.433100224
11.531400239	4.618799779	24.433100224
11.531300413	10.759200502	24.433100224
11.531199636	16.899500211	24.433100224
2.658430014	1.535100044	20.000000298
2.658430014	7.675510237	20.000000298
2.658430014	13.815899981	20.000000298
7.975279821	1.535100044	20.000000298
7.975279821	7.675510237	20.000000298
7.975279821	13.815899981	20.000000298
13.292099918	1.535100044	20.000000298
13.292099918	7.675510237	20.000000298
13.292099918	13.815899981	20.000000298
2.675680022	1.555370035	26.641899347

2.675640091	7.695909791	26.642000675
2.675619888	13.836199617	26.641801000
7.992499881	1.555549968	26.641899347
7.992530305	7.695830187	26.641899347
7.992450443	13.836299534	26.641899347
13.309400315	1.555339977	26.641899347
13.309400315	7.695870263	26.641801000
13.309299538	13.836199617	26.641899347
2.658430014	4.605299994	20.000000298
2.658430014	10.745699619	20.000000298
2.658430014	16.886100342	20.000000298
7.975279821	4.605299994	20.000000298
7.975279821	10.745699619	20.000000298
7.975279821	16.886100342	20.000000298
13.292099918	4.605299994	20.000000298
13.292099918	10.745699619	20.000000298
13.292099918	16.886100342	20.000000298
2.675530044	4.625650138	26.641899347
2.675709970	10.766100271	26.641899347
2.675690004	16.906399979	26.641801000
7.992559777	4.625660020	26.641899347
7.992510339	10.766100271	26.641899347
7.992539812	16.906599813	26.641899347
13.309500141	4.625619943	26.641899347
13.309400315	10.766100271	26.641899347
13.309199711	16.906300062	26.641899347
0.917297980	1.574730066	31.008499861
0.917239985	7.715189944	31.008499861
0.917275994	13.855600000	31.008398533
6.234159909	1.574770006	31.008499861
6.234100013	7.715189944	31.008499861
6.234109996	13.855600000	31.008398533
11.551000447	1.574780025	31.008499861
11.551000447	7.715149867	31.008398533
11.551000447	13.855600000	31.008499861
0.917249017	4.644969818	31.008499861
0.917267973	10.785399639	31.008499861
0.917275994	16.925800362	31.008499861
6.234129961	4.644980249	31.008499861
6.234090031	10.785399639	31.008499861
6.234139944	16.925800362	31.008499861
11.551000447	4.644959936	31.008398533
11.551000447	10.785399639	31.008499861
11.551000447	16.925800362	31.008499861
3.575719913	6.180089763	31.008499861

3.575740116	12.320500368	31.008398533
3.575750098	0.039687942	31.008398533
8.892599668	6.180050235	31.008499861
8.892550230	12.320500368	31.008499861
8.892619633	0.039687942	31.008499861
14.209400275	6.180050235	31.008499861
14.209400275	12.320500368	31.008499861
14.209500102	0.039586927	31.008499861
3.575750098	3.109860030	31.008499861
3.575709930	9.250280243	31.008499861
3.575770063	15.390699632	31.008398533
8.892610126	3.109860030	31.008499861
8.892579703	9.250260479	31.008499861
8.892610126	15.390699632	31.008499861
14.209500102	3.109849874	31.008499861
14.209400275	9.250260479	31.008499861
14.209400275	15.390699632	31.008499861
2.690219980	1.575549990	32.784798741
2.690190032	7.716020024	32.784900069
2.690239945	13.856400435	32.784700394
8.007049822	1.575599949	32.784798741
8.007080245	7.715960184	32.784798741
8.007029857	13.856400435	32.784798741
13.323899867	1.575570029	32.784798741
13.323899867	7.715989830	32.784798741
13.323899867	13.856400435	32.784798741
2.690200014	4.645780135	32.784798741
2.690200014	10.786200073	32.784798741
2.690249928	16.926599699	32.784700394
8.007100210	4.645770253	32.784798741
8.007049822	10.786200073	32.784798741
8.007080245	16.926599699	32.784798741
13.323899867	4.645759822	32.784798741
13.323899867	10.786200073	32.784798741
13.323899867	16.926599699	32.784798741
5.348640011	6.180829808	32.784798741
5.348640011	12.321299705	32.784798741
5.348649994	0.040487278	32.784700394
10.665500039	6.180819926	32.784700394
10.665500039	12.321299705	32.784798741
10.665500039	0.040487278	32.784798741
0.031731496	6.180829808	32.784798741
0.031832273	12.321199788	32.784798741
0.031832273	0.040386263	32.784798741
5.348649994	3.110650034	32.784798741

5.348630028	9.251059816	32.784798741
5.348630028	15.391500067	32.784700394
10.665500039	3.110620114	32.784798741
10.665500039	9.251030170	32.784798741
10.665500039	15.391500067	32.784798741
0.031832273	3.110589919	32.784798741
0.031731496	9.251069698	32.784798741
0.031832273	15.391400150	32.784798741
2.689790013	1.574210031	29.243999720
2.689719897	7.714629969	29.243999720
2.689790013	13.855000498	29.243901372
8.006649565	1.574200012	29.243999720
8.006620093	7.714610205	29.243999720
8.006679989	13.855000498	29.243999720
13.323600388	1.574150053	29.243999720
13.323499611	7.714580011	29.243999720
13.323499611	13.855000498	29.243999720
2.689790013	4.644420274	29.243999720
2.689790013	10.784800136	29.243999720
2.689770048	16.925199761	29.243901372
8.006640058	4.644399962	29.243999720
8.006649565	10.784800136	29.243999720
8.006690447	16.925199761	29.243999720
13.323499611	4.644350003	29.243999720
13.323499611	10.784800136	29.243999720
13.323499611	16.925199761	29.243999720
5.348169876	6.179519906	29.243999720
5.348199824	12.319899768	29.243901372
5.348250213	0.039086243	29.243999720
10.664999956	6.179500143	29.243901372
10.664999956	12.319899768	29.243999720
10.665099782	0.039086243	29.243999720
0.031332190	6.179500143	29.243999720
0.031332190	12.319899768	29.243999720
0.031332190	0.039086243	29.243999720
5.348189841	3.109350014	29.243999720
5.348139928	9.249689524	29.243999720
5.348229772	15.390200047	29.243901372
10.665099782	3.109280017	29.243901372
10.664999956	9.249710386	29.243999720
10.664999956	15.390100130	29.243999720
0.031332190	3.109309937	29.243999720
0.031332190	9.249700504	29.243999720
0.031332190	15.390100130	29.243999720
0.441097987	1.215809943	36.240199208

0.441097987	7.356220274	36.240300536
0.441097987	13.496600136	36.240300536
5.757949785	1.215809943	36.240300536
5.757949785	7.356220274	36.240199208
5.757949785	13.496600136	36.240199208
11.074799831	1.215809943	36.240199208
11.074799831	7.356220274	36.240300536
11.074799831	13.496600136	36.240199208
3.099529992	2.729659903	36.240300536
3.099529992	8.870069959	36.240300536
3.099529992	15.010499779	36.240300536
8.416380037	2.729659903	36.240300536
8.416380037	8.870069959	36.240300536
8.416380037	15.010499779	36.240300536
13.733199659	2.729659903	36.240300536
13.733199659	8.870069959	36.240300536
13.733199659	15.010499779	36.240300536
0.441097987	4.286019912	36.240199208
0.441097987	10.426399774	36.240300536
0.441097987	16.566800497	36.240300536
5.757949785	4.286019912	36.240300536
5.757949785	10.426399774	36.240300536
5.757949785	16.566800497	36.240199208
11.074799831	4.286019912	36.240300536
11.074799831	10.426399774	36.240300536
11.074799831	16.566800497	36.240199208
3.099529992	5.799870146	36.240300536
3.099529992	11.940300515	36.240300536
3.099529992	18.080700141	36.240300536
8.416380037	5.799870146	36.240300536
8.416380037	11.940300515	36.240300536
8.416380037	18.080700141	36.240300536
13.733199659	5.799870146	36.240300536
13.733199659	11.940300515	36.240300536
13.733199659	18.080700141	36.240300536
1.327239988	5.814029824	37.962201238
1.327239988	11.954399803	37.962201238
1.327239988	18.094800527	37.962201238
6.644100016	5.814029824	37.962201238
6.644100016	11.954399803	37.962201238
6.644100016	18.094800527	37.962201238
11.960999975	5.814029824	37.962201238
11.960999975	11.954399803	37.962201238
11.960999975	18.094800527	37.962201238
3.985670002	1.187480020	37.962201238

3.985670002	7.327880058	37.962201238
3.985670002	13.468300545	37.962201238
9.302529792	1.187480020	37.962099910
9.302529792	7.327880058	37.962201238
9.302529792	13.468300545	37.962099910
14.619399803	1.187480020	37.962099910
14.619399803	7.327880058	37.962201238
14.619399803	13.468300545	37.962099910
1.327239988	2.743830011	37.962201238
1.327239988	8.884240067	37.962201238
1.327239988	15.024600165	37.962201238
6.644100016	2.743830011	37.962201238
6.644100016	8.884240067	37.962201238
6.644100016	15.024600165	37.962201238
11.960999975	2.743830011	37.962201238
11.960999975	8.884240067	37.962201238
11.960999975	15.024600165	37.962201238
3.985670002	4.257679970	37.962201238
3.985670002	10.398100183	37.962201238
3.985670002	16.538499808	37.962201238
9.302529792	4.257679970	37.962201238
9.302529792	10.398100183	37.962201238
9.302529792	16.538499808	37.962099910
14.619399803	4.257679970	37.962099910
14.619399803	10.398100183	37.962201238
14.619399803	16.538499808	37.962099910
1.327239988	5.814029824	40.353500843
1.327239988	11.954399803	40.353500843
1.327239988	18.094800527	40.353500843
6.644100016	5.814029824	40.353500843
6.644100016	11.954399803	40.353500843
6.644100016	18.094800527	40.353500843
11.960999975	5.814029824	40.353500843
11.960999975	11.954399803	40.353500843
11.960999975	18.094800527	40.353500843
3.985670002	1.187480020	40.353399515
3.985670002	7.327880058	40.353399515
3.985670002	13.468300545	40.353399515
9.302529792	1.187480020	40.353399515
9.302529792	7.327880058	40.353399515
9.302529792	13.468300545	40.353399515
14.619399803	1.187480020	40.353399515
14.619399803	7.327880058	40.353399515
14.619399803	13.468300545	40.353399515
1.327239988	2.743830011	40.353500843

```
     1.327239988         8.884240067        40.353500843
     1.327239988        15.024600165        40.353500843
     6.644100016         2.743830011        40.353500843
     6.644100016         8.884240067        40.353500843
     6.644100016        15.024600165        40.353500843
    11.960999975         2.743830011        40.353500843
    11.960999975         8.884240067        40.353500843
    11.960999975        15.024600165        40.353500843
     3.985670002         4.257679970        40.353399515
     3.985670002        10.398100183        40.353399515
     3.985670002        16.538499808        40.353399515
     9.302529792         4.257679970        40.353399515
     9.302529792        10.398100183        40.353399515
     9.302529792        16.538499808        40.353399515
    14.619399803         4.257679970        40.353399515
    14.619399803        10.398100183        40.353399515
    14.619399803        16.538499808        40.353399515

Ag/MoS2/Si
1.0
       15.6201000214         0.0000000000         0.0000000000
        0.0000000000        17.9876518250         0.0000000000
        0.0000000000         0.0000000000        50.0000000000
   Ag   Mo    S   Si
  144   36   72  108
Cartesian
     0.000000000         0.000000000        20.000000298
     0.000000000         5.995884120        20.000000298
     0.000000000        11.991768241        20.000000298
     5.206700162         0.000000000        20.000000298
     5.206700162         5.995884120        20.000000298
     5.206700162        11.991768241        20.000000298
    10.413400325         0.000000000        20.000000298
    10.413400325         5.995884120        20.000000298
    10.413400325        11.991768241        20.000000298
     0.128135009         0.071177994        24.562700093
     0.128135009         6.067061855        24.562700093
     0.128135009        12.062945975        24.562700093
     5.334835099         0.071177994        24.562700093
     5.334835099         6.067061855        24.562700093
     5.334835099        12.062945975        24.562700093
    10.541535261         0.071177994        24.562700093
    10.541535261         6.067061855        24.562700093
    10.541535261        12.062945975        24.562700093
     0.000000000         2.997939916        20.000000298
```

0.000000000	8.993823768	20.000000298
0.000000000	14.989707352	20.000000298
5.206700162	2.997939916	20.000000298
5.206700162	8.993823768	20.000000298
5.206700162	14.989707352	20.000000298
10.413400325	2.997939916	20.000000298
10.413400325	8.993823768	20.000000298
10.413400325	14.989707352	20.000000298
0.128135009	3.069120063	24.562700093
0.128135009	9.065003647	24.562700093
0.128135009	15.060888304	24.562700093
5.334835099	3.069120063	24.562700093
5.334835099	9.065003647	24.562700093
5.334835099	15.060888304	24.562700093
10.541535261	3.069120063	24.562700093
10.541535261	9.065003647	24.562700093
10.541535261	15.060888304	24.562700093
1.799930102	0.029904598	22.291800380
1.799930102	6.025788461	22.291800380
1.799930102	12.021672581	22.291800380
7.006630264	0.029904598	22.291800380
7.006630264	6.025788461	22.291800380
7.006630264	12.021672581	22.291800380
12.213329961	0.029904598	22.291800380
12.213329961	6.025788461	22.291800380
12.213329961	12.021672581	22.291800380
1.799930102	3.027849885	22.291800380
1.799930102	9.023733470	22.291800380
1.799930102	15.019618126	22.291800380
7.006630264	3.027849885	22.291800380
7.006630264	9.023733470	22.291800380
7.006630264	15.019618126	22.291800380
12.213329961	3.027849885	22.291800380
12.213329961	9.023733470	22.291800380
12.213329961	15.019618126	22.291800380
3.658500229	0.109276994	26.818400621
3.658500229	6.105161156	26.818400621
3.658500229	12.101044740	26.818400621
8.865199926	0.109276994	26.818400621
8.865199926	6.105161156	26.818400621
8.865199926	12.101044740	26.818400621
14.071900553	0.109276994	26.818400621
14.071900553	6.105161156	26.818400621
14.071900553	12.101044740	26.818400621
3.658500229	3.107219900	26.818400621

3.658500229	9.103103484	26.818400621
3.658500229	15.098988141	26.818400621
8.865199926	3.107219900	26.818400621
8.865199926	9.103103484	26.818400621
8.865199926	15.098988141	26.818400621
14.071900553	3.107219900	26.818400621
14.071900553	9.103103484	26.818400621
14.071900553	15.098988141	26.818400621
4.403280415	1.528879927	22.291800380
4.403280415	7.524763914	22.291800380
4.403280415	13.520647498	22.291800380
9.609980578	1.528879927	22.291800380
9.609980578	7.524763914	22.291800380
9.609980578	13.520647498	22.291800380
14.816680274	1.528879927	22.291800380
14.816680274	7.524763914	22.291800380
14.816680274	13.520647498	22.291800380
4.403280415	4.526819977	22.291800380
4.403280415	10.522704098	22.291800380
4.403280415	16.518587682	22.291800380
9.609980578	4.526819977	22.291800380
9.609980578	10.522704098	22.291800380
9.609980578	16.518587682	22.291800380
14.816680274	4.526819977	22.291800380
14.816680274	10.522704098	22.291800380
14.816680274	16.518587682	22.291800380
1.055150031	1.608249942	26.818400621
1.055150031	7.604133928	26.818400621
1.055150031	13.600017513	26.818400621
6.261850077	1.608249942	26.818400621
6.261850077	7.604133928	26.818400621
6.261850077	13.600017513	26.818400621
11.468550240	1.608249942	26.818400621
11.468550240	7.604133928	26.818400621
11.468550240	13.600017513	26.818400621
1.055150031	4.606189992	26.818400621
1.055150031	10.602074112	26.818400621
1.055150031	16.597957696	26.818400621
6.261850077	4.606189992	26.818400621
6.261850077	10.602074112	26.818400621
6.261850077	16.597957696	26.818400621
11.468550240	4.606189992	26.818400621
11.468550240	10.602074112	26.818400621
11.468550240	16.597957696	26.818400621
2.603350314	1.498969958	20.000000298

2.603350314	7.494853676	20.000000298
2.603350314	13.490737797	20.000000298
7.810050011	1.498969958	20.000000298
7.810050011	7.494853676	20.000000298
7.810050011	13.490737797	20.000000298
13.016750638	1.498969958	20.000000298
13.016750638	7.494853676	20.000000298
13.016750638	13.490737797	20.000000298
2.731480362	1.570149971	24.562700093
2.731480362	7.566034091	24.562700093
2.731480362	13.561917676	24.562700093
7.938180292	1.570149971	24.562700093
7.938180292	7.566034091	24.562700093
7.938180292	13.561917676	24.562700093
13.144879989	1.570149971	24.562700093
13.144879989	7.566034091	24.562700093
13.144879989	13.561917676	24.562700093
2.603350314	4.496909740	20.000000298
2.603350314	10.492793324	20.000000298
2.603350314	16.488677980	20.000000298
7.810050011	4.496909740	20.000000298
7.810050011	10.492793324	20.000000298
7.810050011	16.488677980	20.000000298
13.016750638	4.496909740	20.000000298
13.016750638	10.492793324	20.000000298
13.016750638	16.488677980	20.000000298
2.731480362	4.568090155	24.562700093
2.731480362	10.563974275	24.562700093
2.731480362	16.559857859	24.562700093
7.938180292	4.568090155	24.562700093
7.938180292	10.563974275	24.562700093
7.938180292	16.559857859	24.562700093
13.144879989	4.568090155	24.562700093
13.144879989	10.563974275	24.562700093
13.144879989	16.559857859	24.562700093
4.768780185	1.745140005	30.968898535
4.768780185	7.741024125	30.968898535
4.768780185	13.736908246	30.968898535
9.975479881	1.745140005	30.968898535
9.975479881	7.741024125	30.968898535
9.975479881	13.736908246	30.968898535
15.182180509	1.745140005	30.968898535
15.182180509	7.741024125	30.968898535
15.182180509	13.736908246	30.968898535
4.768780185	4.743079653	30.968898535

4.768780185	10.738963773	30.968898535
4.768780185	16.734847357	30.968898535
9.975479881	4.743079653	30.968898535
9.975479881	10.738963773	30.968898535
9.975479881	16.734847357	30.968898535
15.182180509	4.743079653	30.968898535
15.182180509	10.738963773	30.968898535
15.182180509	16.734847357	30.968898535
2.165490155	0.246140982	30.968898535
2.165490155	6.242025085	30.968898535
2.165490155	12.237908670	30.968898535
7.372190084	0.246140982	30.968898535
7.372190084	6.242025085	30.968898535
7.372190084	12.237908670	30.968898535
12.578889781	0.246140982	30.968898535
12.578889781	6.242025085	30.968898535
12.578889781	12.237908670	30.968898535
2.165490155	3.244079809	30.968898535
2.165490155	9.239964197	30.968898535
2.165490155	15.235847781	30.968898535
7.372190084	3.244079809	30.968898535
7.372190084	9.239964197	30.968898535
7.372190084	15.235847781	30.968898535
12.578889781	3.244079809	30.968898535
12.578889781	9.239964197	30.968898535
12.578889781	15.235847781	30.968898535
1.291740014	1.742209960	29.339799285
1.291740014	7.738093946	29.339799285
1.291740014	13.733978066	29.339799285
6.498439827	1.742209960	29.339799285
6.498439827	7.738093946	29.339799285
6.498439827	13.733978066	29.339799285
11.705139989	1.742209960	29.339799285
11.705139989	7.738093946	29.339799285
11.705139989	13.733978066	29.339799285
1.291740014	4.740160195	29.339799285
1.291740014	10.736044315	29.339799285
1.291740014	16.731927900	29.339799285
6.498439827	4.740160195	29.339799285
6.498439827	10.736044315	29.339799285
6.498439827	16.731927900	29.339799285
11.705139989	4.740160195	29.339799285
11.705139989	10.736044315	29.339799285
11.705139989	16.731927900	29.339799285
3.895110229	0.243256000	29.339700937

3.895110229	6.239139936	29.339700937
3.895110229	12.235023520	29.339700937
9.101810158	0.243256000	29.339700937
9.101810158	6.239139936	29.339700937
9.101810158	12.235023520	29.339700937
14.308509855	0.243256000	29.339700937
14.308509855	6.239139936	29.339700937
14.308509855	12.235023520	29.339700937
3.895110229	3.241199752	29.339700937
3.895110229	9.237083337	29.339700937
3.895110229	15.232967993	29.339700937
9.101810158	3.241199752	29.339700937
9.101810158	9.237083337	29.339700937
9.101810158	15.232967993	29.339700937
14.308509855	3.241199752	29.339700937
14.308509855	9.237083337	29.339700937
14.308509855	15.232967993	29.339700937
1.300430020	1.747209920	32.605299354
1.300430020	7.743093906	32.605299354
1.300430020	13.738977491	32.605299354
6.507130066	1.747209920	32.605299354
6.507130066	7.743093906	32.605299354
6.507130066	13.738977491	32.605299354
11.713830228	1.747209920	32.605299354
11.713830228	7.743093906	32.605299354
11.713830228	13.738977491	32.605299354
1.300430020	4.745149970	32.605299354
1.300430020	10.741034090	32.605299354
1.300430020	16.736917674	32.605299354
6.507130066	4.745149970	32.605299354
6.507130066	10.741034090	32.605299354
6.507130066	16.736917674	32.605299354
11.713830228	4.745149970	32.605299354
11.713830228	10.741034090	32.605299354
11.713830228	16.736917674	32.605299354
3.903760433	0.248339002	32.605499029
3.903760433	6.244222988	32.605499029
3.903760433	12.240106572	32.605499029
9.110460363	0.248339002	32.605499029
9.110460363	6.244222988	32.605499029
9.110460363	12.240106572	32.605499029
14.317160059	0.248339002	32.605499029
14.317160059	6.244222988	32.605499029
14.317160059	12.240106572	32.605499029
3.903760433	3.246279856	32.605499029

3.903760433	9.242164244	32.605499029
3.903760433	15.238047828	32.605499029
9.110460363	3.246279856	32.605499029
9.110460363	9.242164244	32.605499029
9.110460363	15.238047828	32.605499029
14.317160059	3.246279856	32.605499029
14.317160059	9.242164244	32.605499029
14.317160059	15.238047828	32.605499029
0.431960015	1.187199890	35.576900840
0.431960015	7.183083876	35.576900840
0.431960015	13.178967997	35.576900840
5.638660148	1.187199890	35.576900840
5.638660148	7.183083876	35.576900840
5.638660148	13.178967997	35.576900840
10.845360310	1.187199890	35.576900840
10.845360310	7.183083876	35.576900840
10.845360310	13.178967997	35.576900840
3.035310300	2.665419771	35.576701164
3.035310300	8.661303624	35.576701164
3.035310300	14.657187208	35.576701164
8.242009997	2.665419771	35.576701164
8.242009997	8.661303624	35.576701164
8.242009997	14.657187208	35.576701164
13.448710624	2.665419771	35.576701164
13.448710624	8.661303624	35.576701164
13.448710624	14.657187208	35.576701164
0.431960015	4.185139940	35.576900840
0.431960015	10.181023524	35.576900840
0.431960015	16.176908181	35.576900840
5.638660148	4.185139940	35.576900840
5.638660148	10.181023524	35.576900840
5.638660148	16.176908181	35.576900840
10.845360310	4.185139940	35.576900840
10.845360310	10.181023524	35.576900840
10.845360310	16.176908181	35.576900840
3.035310300	5.663359687	35.576701164
3.035310300	11.659243808	35.576701164
3.035310300	17.655127392	35.576701164
8.242009997	5.663359687	35.576701164
8.242009997	11.659243808	35.576701164
8.242009997	17.655127392	35.576701164
13.448710624	5.663359687	35.576701164
13.448710624	11.659243808	35.576701164
13.448710624	17.655127392	35.576701164
1.299740126	5.677189855	37.355598807

1.299740126	11.673073440	37.355598807
1.299740126	17.668958096	37.355598807
6.506440172	5.677189855	37.355598807
6.506440172	11.673073440	37.355598807
6.506440172	17.668958096	37.355598807
11.713140335	5.677189855	37.355598807
11.713140335	11.673073440	37.355598807
11.713140335	17.668958096	37.355598807
3.903090324	1.159529905	37.355500460
3.903090324	7.155413891	37.355500460
3.903090324	13.151298012	37.355500460
9.109790021	1.159529905	37.355500460
9.109790021	7.155413891	37.355500460
9.109790021	13.151298012	37.355500460
14.316490649	1.159529905	37.355500460
14.316490649	7.155413891	37.355500460
14.316490649	13.151298012	37.355500460
1.299740126	2.679249939	37.355598807
1.299740126	8.675133792	37.355598807
1.299740126	14.671017912	37.355598807
6.506440172	2.679249939	37.355598807
6.506440172	8.675133792	37.355598807
6.506440172	14.671017912	37.355598807
11.713140335	2.679249939	37.355598807
11.713140335	8.675133792	37.355598807
11.713140335	14.671017912	37.355598807
3.903090324	4.157469955	37.355500460
3.903090324	10.153353539	37.355500460
3.903090324	16.149238195	37.355500460
9.109790021	4.157469955	37.355500460
9.109790021	10.153353539	37.355500460
9.109790021	16.149238195	37.355500460
14.316490649	4.157469955	37.355500460
14.316490649	10.153353539	37.355500460
14.316490649	16.149238195	37.355500460
1.299740126	5.677189855	39.734199643
1.299740126	11.673073440	39.734199643
1.299740126	17.668958096	39.734199643
6.506440172	5.677189855	39.734199643
6.506440172	11.673073440	39.734199643
6.506440172	17.668958096	39.734199643
11.713140335	5.677189855	39.734199643
11.713140335	11.673073440	39.734199643
11.713140335	17.668958096	39.734199643
3.903090324	1.159529905	39.734199643

```
     3.903090324         7.155413891        39.734199643
     3.903090324        13.151298012        39.734199643
     9.109790021         1.159529905        39.734199643
     9.109790021         7.155413891        39.734199643
     9.109790021        13.151298012        39.734199643
    14.316490649         1.159529905        39.734199643
    14.316490649         7.155413891        39.734199643
    14.316490649        13.151298012        39.734199643
     1.299740126         2.679249939        39.734199643
     1.299740126         8.675133792        39.734199643
     1.299740126        14.671017912        39.734199643
     6.506440172         2.679249939        39.734199643
     6.506440172         8.675133792        39.734199643
     6.506440172        14.671017912        39.734199643
    11.713140335         2.679249939        39.734199643
    11.713140335         8.675133792        39.734199643
    11.713140335        14.671017912        39.734199643
     3.903090324         4.157469955        39.734199643
     3.903090324        10.153353539        39.734199643
     3.903090324        16.149238195        39.734199643
     9.109790021         4.157469955        39.734199643
     9.109790021        10.153353539        39.734199643
     9.109790021        16.149238195        39.734199643
    14.316490649         4.157469955        39.734199643
    14.316490649        10.153353539        39.734199643
    14.316490649        16.149238195        39.734199643

Ag/MoSe2/Si
1.0
       15.9722995758         0.0000000000         0.0000000000
        0.0000000000        18.3864002228         0.0000000000
        0.0000000000         0.0000000000        50.0000000000
   Ag   Mo   Se   Si
  144   36   72  108
Cartesian
     5.324110014         0.000000000        20.000000298
     5.324110014         6.128800257        20.000000298
     5.324110014        12.257600514        20.000000298
    10.648209555         0.000000000        20.000000298
    10.648209555         6.128800257        20.000000298
    10.648209555        12.257600514        20.000000298
     0.000010155         0.000000000        20.000000298
     0.000010155         6.128800257        20.000000298
     0.000010155        12.257600514        20.000000298
     5.272609840         6.086380133        26.672500372
```

5.272609840	12.215179842	26.672500372
5.272609840	18.343980647	26.672500372
10.596709857	6.086380133	26.672500372
10.596709857	12.215179842	26.672500372
10.596709857	18.343980647	26.672500372
15.920809399	6.086380133	26.672500372
15.920809399	12.215179842	26.672500372
15.920809399	18.343980647	26.672500372
5.324110014	3.064400128	20.000000298
5.324110014	9.193200111	20.000000298
5.324110014	15.321999820	20.000000298
10.648209555	3.064400128	20.000000298
10.648209555	9.193200111	20.000000298
10.648209555	15.321999820	20.000000298
0.000010155	3.064400128	20.000000298
0.000010155	9.193200111	20.000000298
0.000010155	15.321999820	20.000000298
5.272609840	3.021980004	26.672500372
5.272609840	9.150779987	26.672500372
5.272609840	15.279580244	26.672500372
10.596709857	3.021980004	26.672500372
10.596709857	9.150779987	26.672500372
10.596709857	15.279580244	26.672500372
15.920809399	3.021980004	26.672500372
15.920809399	9.150779987	26.672500372
15.920809399	15.279580244	26.672500372
1.756749925	6.113820197	22.237199545
1.756749925	12.242620454	22.237199545
1.756749925	18.371420163	22.237199545
7.080849942	6.113820197	22.237199545
7.080849942	12.242620454	22.237199545
7.080849942	18.371420163	22.237199545
12.404949483	6.113820197	22.237199545
12.404949483	12.242620454	22.237199545
12.404949483	18.371420163	22.237199545
1.756749925	3.049420068	22.237199545
1.756749925	9.178220051	22.237199545
1.756749925	15.307019760	22.237199545
7.080849942	3.049420068	22.237199545
7.080849942	9.178220051	22.237199545
7.080849942	15.307019760	22.237199545
12.404949483	3.049420068	22.237199545
12.404949483	9.178220051	22.237199545
12.404949483	15.307019760	22.237199545
3.513999900	6.099400149	24.461200833

3.513999900	12.228200406	24.461200833
3.513999900	18.357000115	24.461200833
8.838100155	6.099400149	24.461200833
8.838100155	12.228200406	24.461200833
8.838100155	18.357000115	24.461200833
14.162199696	6.099400149	24.461200833
14.162199696	12.228200406	24.461200833
14.162199696	18.357000115	24.461200833
3.514009896	3.035000021	24.461200833
3.514009896	9.163800004	24.461200833
3.514009896	15.292599713	24.461200833
8.838109675	3.035000021	24.461200833
8.838109675	9.163800004	24.461200833
8.838109675	15.292599713	24.461200833
14.162209217	3.035000021	24.461200833
14.162209217	9.163800004	24.461200833
14.162209217	15.292599713	24.461200833
4.418799695	1.517220004	22.237199545
4.418799695	7.646019850	22.237199545
4.418799695	13.774820107	22.237199545
9.742899237	1.517220004	22.237199545
9.742899237	7.646019850	22.237199545
9.742899237	13.774820107	22.237199545
15.066999730	1.517220004	22.237199545
15.066999730	7.646019850	22.237199545
15.066999730	13.774820107	22.237199545
4.418799695	4.581620270	22.237199545
4.418799695	10.710420800	22.237199545
4.418799695	16.839220509	22.237199545
9.742899237	4.581620270	22.237199545
9.742899237	10.710420800	22.237199545
9.742899237	16.839220509	22.237199545
15.066999730	4.581620270	22.237199545
15.066999730	10.710420800	22.237199545
15.066999730	16.839220509	22.237199545
0.851953937	1.502799956	24.461200833
0.851953937	7.631599802	24.461200833
0.851953937	13.760400059	24.461200833
6.176053717	1.502799956	24.461200833
6.176053717	7.631599802	24.461200833
6.176053717	13.760400059	24.461200833
11.500153258	1.502799956	24.461200833
11.500153258	7.631599802	24.461200833
11.500153258	13.760400059	24.461200833
0.851953937	4.567200222	24.461200833

0.851953937	10.696000753	24.461200833
0.851953937	16.824800462	24.461200833
6.176053717	4.567200222	24.461200833
6.176053717	10.696000753	24.461200833
6.176053717	16.824800462	24.461200833
11.500153258	4.567200222	24.461200833
11.500153258	10.696000753	24.461200833
11.500153258	16.824800462	24.461200833
2.662060005	1.532200064	20.000000298
2.662060005	7.660999910	20.000000298
2.662060005	13.789800167	20.000000298
7.986160260	1.532200064	20.000000298
7.986160260	7.660999910	20.000000298
7.986160260	13.789800167	20.000000298
13.310259801	1.532200064	20.000000298
13.310259801	7.660999910	20.000000298
13.310259801	13.789800167	20.000000298
2.610559832	1.489779940	26.672500372
2.610559832	7.618579786	26.672500372
2.610559832	13.747380591	26.672500372
7.934659611	1.489779940	26.672500372
7.934659611	7.618579786	26.672500372
7.934659611	13.747380591	26.672500372
13.258759152	1.489779940	26.672500372
13.258759152	7.618579786	26.672500372
13.258759152	13.747380591	26.672500372
2.662060005	4.596590192	20.000000298
2.662060005	10.725389901	20.000000298
2.662060005	16.854190706	20.000000298
7.986160260	4.596590192	20.000000298
7.986160260	10.725389901	20.000000298
7.986160260	16.854190706	20.000000298
13.310259801	4.596590192	20.000000298
13.310259801	10.725389901	20.000000298
13.310259801	16.854190706	20.000000298
2.610559832	4.554180205	26.672500372
2.610559832	10.682980188	26.672500372
2.610559832	16.811779897	26.672500372
7.934659611	4.554180205	26.672500372
7.934659611	10.682980188	26.672500372
7.934659611	16.811779897	26.672500372
13.258759152	4.554180205	26.672500372
13.258759152	10.682980188	26.672500372
13.258759152	16.811779897	26.672500372
0.758539037	1.413870028	31.071400642

0.758539037	7.542670148	31.071400642
0.758539037	13.671469857	31.071400642
6.082638816	1.413870028	31.071400642
6.082638816	7.542670148	31.071400642
6.082638816	13.671469857	31.071400642
11.406738833	1.413870028	31.071400642
11.406738833	7.542670148	31.071400642
11.406738833	13.671469857	31.071400642
0.758539037	4.478270019	31.071400642
0.758539037	10.607070550	31.071400642
0.758539037	16.735870259	31.071400642
6.082638816	4.478270019	31.071400642
6.082638816	10.607070550	31.071400642
6.082638816	16.735870259	31.071400642
11.406738833	4.478270019	31.071400642
11.406738833	10.607070550	31.071400642
11.406738833	16.735870259	31.071400642
3.420599756	6.010439809	31.071400642
3.420599756	12.139239518	31.071400642
3.420599756	18.268040323	31.071400642
8.744700011	6.010439809	31.071400642
8.744700011	12.139239518	31.071400642
8.744700011	18.268040323	31.071400642
14.068799552	6.010439809	31.071400642
14.068799552	12.139239518	31.071400642
14.068799552	18.268040323	31.071400642
3.420599756	2.946039954	31.071400642
3.420599756	9.074840211	31.071400642
3.420599756	15.203639920	31.071400642
8.744700011	2.946039954	31.071400642
8.744700011	9.074840211	31.071400642
8.744700011	15.203639920	31.071400642
14.068799552	2.946039954	31.071400642
14.068799552	9.074840211	31.071400642
14.068799552	15.203639920	31.071400642
2.530689842	1.412520135	32.844799757
2.530689842	7.541319981	32.844799757
2.530689842	13.670120785	32.844799757
7.854789621	1.412520135	32.844799757
7.854789621	7.541319981	32.844799757
7.854789621	13.670120785	32.844799757
13.178889162	1.412520135	32.844799757
13.178889162	7.541319981	32.844799757
13.178889162	13.670120785	32.844799757
2.530689842	4.476920400	32.844799757

2.530689842	10.605720383	32.844799757
2.530689842	16.734520092	32.844799757
7.854789621	4.476920400	32.844799757
7.854789621	10.605720383	32.844799757
7.854789621	16.734520092	32.844799757
13.178889162	4.476920400	32.844799757
13.178889162	10.605720383	32.844799757
13.178889162	16.734520092	32.844799757
5.192739850	6.009240330	32.844799757
5.192739850	12.138040587	32.844799757
5.192739850	18.266840296	32.844799757
10.516839868	6.009240330	32.844799757
10.516839868	12.138040587	32.844799757
10.516839868	18.266840296	32.844799757
15.840939409	6.009240330	32.844799757
15.840939409	12.138040587	32.844799757
15.840939409	18.266840296	32.844799757
5.192739850	2.944839928	32.844799757
5.192739850	9.073640184	32.844799757
5.192739850	15.202439893	32.844799757
10.516839868	2.944839928	32.844799757
10.516839868	9.073640184	32.844799757
10.516839868	15.202439893	32.844799757
15.840939409	2.944839928	32.844799757
15.840939409	9.073640184	32.844799757
15.840939409	15.202439893	32.844799757
2.532909960	1.414860050	29.316899180
2.532909960	7.543660307	29.316899180
2.532909960	13.672460564	29.316899180
7.857009739	1.414860050	29.316899180
7.857009739	7.543660307	29.316899180
7.857009739	13.672460564	29.316899180
13.181109280	1.414860050	29.316899180
13.181109280	7.543660307	29.316899180
13.181109280	13.672460564	29.316899180
2.532909960	4.479260178	29.316899180
2.532909960	10.608060161	29.316899180
2.532909960	16.736859870	29.316899180
7.857009739	4.479260178	29.316899180
7.857009739	10.608060161	29.316899180
7.857009739	16.736859870	29.316899180
13.181109280	4.479260178	29.316899180
13.181109280	10.608060161	29.316899180
13.181109280	16.736859870	29.316899180
5.194959968	6.011479832	29.316899180

5.194959968	12.140279541	29.316899180
5.194959968	18.269080346	29.316899180
10.519059986	6.011479832	29.316899180
10.519059986	12.140279541	29.316899180
10.519059986	18.269080346	29.316899180
15.843159527	6.011479832	29.316899180
15.843159527	12.140279541	29.316899180
15.843159527	18.269080346	29.316899180
5.194959968	2.947079978	29.316899180
5.194959968	9.075880234	29.316899180
5.194959968	15.204679943	29.316899180
10.519059986	2.947079978	29.316899180
10.519059986	9.075880234	29.316899180
10.519059986	15.204679943	29.316899180
15.843159527	2.947079978	29.316899180
15.843159527	9.075880234	29.316899180
15.843159527	15.204679943	29.316899180
0.441700971	1.213520080	35.948398709
0.441700971	7.342319926	35.948398709
0.441700971	13.471120183	35.948398709
5.765800721	1.213520080	35.948398709
5.765800721	7.342319926	35.948398709
5.765800721	13.471120183	35.948398709
11.089900738	1.213520080	35.948398709
11.089900738	7.342319926	35.948398709
11.089900738	13.471120183	35.948398709
3.103759756	2.724499945	35.948398709
3.103759756	8.853299928	35.948398709
3.103759756	14.982099637	35.948398709
8.427860012	2.724499945	35.948398709
8.427860012	8.853299928	35.948398709
8.427860012	14.982099637	35.948398709
13.751959553	2.724499945	35.948398709
13.751959553	8.853299928	35.948398709
13.751959553	14.982099637	35.948398709
0.441700971	4.277910208	35.948398709
0.441700971	10.406709917	35.948398709
0.441700971	16.535510722	35.948398709
5.765800721	4.277910208	35.948398709
5.765800721	10.406709917	35.948398709
5.765800721	16.535510722	35.948398709
11.089900738	4.277910208	35.948398709
11.089900738	10.406709917	35.948398709
11.089900738	16.535510722	35.948398709
3.103759756	5.788900073	35.948398709

3.103759756	11.917700330	35.948398709
3.103759756	18.046500039	35.948398709
8.427860012	5.788900073	35.948398709
8.427860012	11.917700330	35.948398709
8.427860012	18.046500039	35.948398709
13.751959553	5.788900073	35.948398709
13.751959553	11.917700330	35.948398709
13.751959553	18.046500039	35.948398709
1.329049913	5.803040115	37.684699893
1.329049913	11.931839823	37.684699893
1.329049913	18.060640628	37.684699893
6.653149692	5.803040115	37.684699893
6.653149692	11.931839823	37.684699893
6.653149692	18.060640628	37.684699893
11.977249234	5.803040115	37.684699893
11.977249234	11.931839823	37.684699893
11.977249234	18.060640628	37.684699893
3.991109918	1.185229997	37.684601545
3.991109918	7.314029980	37.684601545
3.991109918	13.442830237	37.684601545
9.315209459	1.185229997	37.684601545
9.315209459	7.314029980	37.684601545
9.315209459	13.442830237	37.684601545
14.639309953	1.185229997	37.684601545
14.639309953	7.314029980	37.684601545
14.639309953	13.442830237	37.684601545
1.329049913	2.738639986	37.684699893
1.329049913	8.867439969	37.684699893
1.329049913	14.996240226	37.684699893
6.653149692	2.738639986	37.684699893
6.653149692	8.867439969	37.684699893
6.653149692	14.996240226	37.684699893
11.977249234	2.738639986	37.684699893
11.977249234	8.867439969	37.684699893
11.977249234	14.996240226	37.684699893
3.991109918	4.249630125	37.684601545
3.991109918	10.378429834	37.684601545
3.991109918	16.507230639	37.684601545
9.315209459	4.249630125	37.684601545
9.315209459	10.378429834	37.684601545
9.315209459	16.507230639	37.684601545
14.639309953	4.249630125	37.684601545
14.639309953	10.378429834	37.684601545
14.639309953	16.507230639	37.684601545
1.329049913	5.803040115	40.072602034

```
     1.329049913        11.931839823        40.072602034
     1.329049913        18.060640628        40.072602034
     6.653149692         5.803040115        40.072602034
     6.653149692        11.931839823        40.072602034
     6.653149692        18.060640628        40.072602034
    11.977249234         5.803040115        40.072602034
    11.977249234        11.931839823        40.072602034
    11.977249234        18.060640628        40.072602034
     3.991109918         1.185229997        40.072602034
     3.991109918         7.314029980        40.072602034
     3.991109918        13.442830237        40.072602034
     9.315209459         1.185229997        40.072602034
     9.315209459         7.314029980        40.072602034
     9.315209459        13.442830237        40.072602034
    14.639309953         1.185229997        40.072602034
    14.639309953         7.314029980        40.072602034
    14.639309953        13.442830237        40.072602034
     1.329049913         2.738639986        40.072602034
     1.329049913         8.867439969        40.072602034
     1.329049913        14.996240226        40.072602034
     6.653149692         2.738639986        40.072602034
     6.653149692         8.867439969        40.072602034
     6.653149692        14.996240226        40.072602034
    11.977249234         2.738639986        40.072602034
    11.977249234         8.867439969        40.072602034
    11.977249234        14.996240226        40.072602034
     3.991109918         4.249630125        40.072602034
     3.991109918        10.378429834        40.072602034
     3.991109918        16.507230639        40.072602034
     9.315209459         4.249630125        40.072602034
     9.315209459        10.378429834        40.072602034
     9.315209459        16.507230639        40.072602034
    14.639309953         4.249630125        40.072602034
    14.639309953        10.378429834        40.072602034
    14.639309953        16.507230639        40.072602034

Ag/WS2/Si
1.0
       15.6965999603         0.0000000000         0.0000000000
        0.0000000000        17.9897994995         0.0000000000
        0.0000000000         0.0000000000        50.0000000000
   Ag     W     S    Si
  144    36    72   108
Cartesian
     5.232180028         0.000000000        20.000000298
```

5.232180028	5.996600012	20.000000298
5.232180028	11.993200024	20.000000298
10.464379702	0.000000000	20.000000298
10.464379702	5.996600012	20.000000298
10.464379702	11.993200024	20.000000298
15.696580313	0.000000000	20.000000298
15.696580313	5.996600012	20.000000298
15.696580313	11.993200024	20.000000298
5.160210214	5.948310080	26.780399680
5.160210214	11.944910092	26.780399680
5.160210214	17.941509567	26.780399680
10.392410357	5.948310080	26.780399680
10.392410357	11.944910092	26.780399680
10.392410357	17.941509567	26.780399680
15.624610032	5.948310080	26.780399680
15.624610032	11.944910092	26.780399680
15.624610032	17.941509567	26.780399680
5.232180028	2.998279901	20.000000298
5.232180028	8.994879913	20.000000298
5.232180028	14.991479925	20.000000298
10.464379702	2.998279901	20.000000298
10.464379702	8.994879913	20.000000298
10.464379702	14.991479925	20.000000298
15.696580313	2.998279901	20.000000298
15.696580313	8.994879913	20.000000298
15.696580313	14.991479925	20.000000298
5.160210214	2.950010074	26.780399680
5.160210214	8.946609818	26.780399680
5.160210214	14.943209293	26.780399680
10.392410357	2.950010074	26.780399680
10.392410357	8.946609818	26.780399680
10.392410357	14.943209293	26.780399680
15.624610032	2.950010074	26.780399680
15.624610032	8.946609818	26.780399680
15.624610032	14.943209293	26.780399680
1.719030046	5.979379802	22.276699543
1.719030046	11.975979277	22.276699543
1.719030046	17.972579826	22.276699543
6.951229838	5.979379802	22.276699543
6.951229838	11.975979277	22.276699543
6.951229838	17.972579826	22.276699543
12.183430448	5.979379802	22.276699543
12.183430448	11.975979277	22.276699543
12.183430448	17.972579826	22.276699543
1.719030046	2.981079796	22.276699543

1.719030046	8.977679540	22.276699543
1.719030046	14.974279552	22.276699543
6.951229838	2.981079796	22.276699543
6.951229838	8.977679540	22.276699543
6.951229838	14.974279552	22.276699543
12.183430448	2.981079796	22.276699543
12.183430448	8.977679540	22.276699543
12.183430448	14.974279552	22.276699543
3.439080119	5.963379841	24.534900486
3.439080119	11.959979853	24.534900486
3.439080119	17.956579329	24.534900486
8.671279794	5.963379841	24.534900486
8.671279794	11.959979853	24.534900486
8.671279794	17.956579329	24.534900486
13.903480404	5.963379841	24.534900486
13.903480404	11.959979853	24.534900486
13.903480404	17.956579329	24.534900486
3.439090177	2.965079835	24.534900486
3.439090177	8.961679579	24.534900486
3.439090177	14.958279055	24.534900486
8.671290085	2.965079835	24.534900486
8.671290085	8.961679579	24.534900486
8.671290085	14.958279055	24.534900486
13.903489760	2.965079835	24.534900486
13.903489760	8.961679579	24.534900486
13.903489760	14.958279055	24.534900486
4.335130000	1.481930061	22.276699543
4.335130000	7.478529939	22.276699543
4.335130000	13.475129951	22.276699543
9.567330143	1.481930061	22.276699543
9.567330143	7.478529939	22.276699543
9.567330143	13.475129951	22.276699543
14.799529818	1.481930061	22.276699543
14.799529818	7.478529939	22.276699543
14.799529818	13.475129951	22.276699543
4.335140292	4.480229933	22.276699543
4.335140292	10.476829677	22.276699543
4.335140292	16.473429152	22.276699543
9.567340434	4.480229933	22.276699543
9.567340434	10.476829677	22.276699543
9.567340434	16.473429152	22.276699543
14.799540109	4.480229933	22.276699543
14.799540109	10.476829677	22.276699543
14.799540109	16.473429152	22.276699543
0.822988000	1.465929966	24.534900486

0.822988000	7.462529978	24.534900486
0.822988000	13.459129454	24.534900486
6.055187909	1.465929966	24.534900486
6.055187909	7.462529978	24.534900486
6.055187909	13.459129454	24.534900486
11.287387584	1.465929966	24.534900486
11.287387584	7.462529978	24.534900486
11.287387584	13.459129454	24.534900486
0.822987006	4.464229972	24.534900486
0.822987006	10.460830252	24.534900486
0.822987006	16.457429728	24.534900486
6.055186973	4.464229972	24.534900486
6.055186973	10.460830252	24.534900486
6.055186973	16.457429728	24.534900486
11.287386648	4.464229972	24.534900486
11.287386648	10.460830252	24.534900486
11.287386648	16.457429728	24.534900486
2.616090014	1.499139950	20.000000298
2.616090014	7.495739962	20.000000298
2.616090014	13.492339974	20.000000298
7.848290156	1.499139950	20.000000298
7.848290156	7.495739962	20.000000298
7.848290156	13.492339974	20.000000298
13.080490299	1.499139950	20.000000298
13.080490299	7.495739962	20.000000298
13.080490299	13.492339974	20.000000298
2.544110143	1.450860071	26.780399680
2.544110143	7.447459681	26.780399680
2.544110143	13.444059693	26.780399680
7.776310052	1.450860071	26.780399680
7.776310052	7.447459681	26.780399680
7.776310052	13.444059693	26.780399680
13.008509727	1.450860071	26.780399680
13.008509727	7.447459681	26.780399680
13.008509727	13.444059693	26.780399680
2.616090014	4.497419851	20.000000298
2.616090014	10.494019327	20.000000298
2.616090014	16.490619875	20.000000298
7.848290156	4.497419851	20.000000298
7.848290156	10.494019327	20.000000298
7.848290156	16.490619875	20.000000298
13.080490299	4.497419851	20.000000298
13.080490299	10.494019327	20.000000298
13.080490299	16.490619875	20.000000298
2.544110143	4.449159943	26.780399680

2.544110143	10.445759419	26.780399680
2.544110143	16.442359967	26.780399680
7.776310052	4.449159943	26.780399680
7.776310052	10.445759419	26.780399680
7.776310052	16.442359967	26.780399680
13.008509727	4.449159943	26.780399680
13.008509727	10.445759419	26.780399680
13.008509727	16.442359967	26.780399680
3.371540092	2.901329834	30.936500430
3.371540092	8.897929577	30.936500430
3.371540092	14.894529053	30.936500430
8.603740468	2.901329834	30.936500430
8.603740468	8.897929577	30.936500430
8.603740468	14.894529053	30.936500430
13.835940143	2.901329834	30.936500430
13.835940143	8.897929577	30.936500430
13.835940143	14.894529053	30.936500430
3.371540092	5.899629839	30.936500430
3.371540092	11.896229851	30.936500430
3.371540092	17.892829327	30.936500430
8.603740468	5.899629839	30.936500430
8.603740468	11.896229851	30.936500430
8.603740468	17.892829327	30.936500430
13.835940143	5.899629839	30.936500430
13.835940143	11.896229851	30.936500430
13.835940143	17.892829327	30.936500430
0.755415987	1.402199936	30.936500430
0.755415987	7.398799814	30.936500430
0.755415987	13.395399825	30.936500430
5.987615838	1.402199936	30.936500430
5.987615838	7.398799814	30.936500430
5.987615838	13.395399825	30.936500430
11.219815513	1.402199936	30.936500430
11.219815513	7.398799814	30.936500430
11.219815513	13.395399825	30.936500430
0.755415987	4.400499808	30.936500430
0.755415987	10.397099551	30.936500430
0.755415987	16.393699027	30.936500430
5.987615838	4.400499808	30.936500430
5.987615838	10.397099551	30.936500430
5.987615838	16.393699027	30.936500430
11.219815513	4.400499808	30.936500430
11.219815513	10.397099551	30.936500430
11.219815513	16.393699027	30.936500430
5.111970245	2.900420008	32.579100132

5.111970245	8.897019752	32.579100132
5.111970245	14.893619763	32.579100132
10.344170387	2.900420008	32.579100132
10.344170387	8.897019752	32.579100132
10.344170387	14.893619763	32.579100132
15.576370062	2.900420008	32.579100132
15.576370062	8.897019752	32.579100132
15.576370062	14.893619763	32.579100132
5.111970245	5.898719477	32.579100132
5.111970245	11.895319489	32.579100132
5.111970245	17.891918965	32.579100132
10.344170387	5.898719477	32.579100132
10.344170387	11.895319489	32.579100132
10.344170387	17.891918965	32.579100132
15.576370062	5.898719477	32.579100132
15.576370062	11.895319489	32.579100132
15.576370062	17.891918965	32.579100132
2.495870173	1.401149910	32.579100132
2.495870173	7.397749520	32.579100132
2.495870173	13.394350068	32.579100132
7.728070082	1.401149910	32.579100132
7.728070082	7.397749520	32.579100132
7.728070082	13.394350068	32.579100132
12.960269757	1.401149910	32.579100132
12.960269757	7.397749520	32.579100132
12.960269757	13.394350068	32.579100132
2.495870173	4.399449514	32.579100132
2.495870173	10.396049794	32.579100132
2.495870173	16.392649269	32.579100132
7.728070082	4.399449514	32.579100132
7.728070082	10.396049794	32.579100132
7.728070082	16.392649269	32.579100132
12.960269757	4.399449514	32.579100132
12.960269757	10.396049794	32.579100132
12.960269757	16.392649269	32.579100132
5.113719798	2.902349836	29.305401444
5.113719798	8.898949848	29.305401444
5.113719798	14.895549859	29.305401444
10.345919941	2.902349836	29.305401444
10.345919941	8.898949848	29.305401444
10.345919941	14.895549859	29.305401444
15.578119616	2.902349836	29.305401444
15.578119616	8.898949848	29.305401444
15.578119616	14.895549859	29.305401444
5.113719798	5.900649574	29.305401444

5.113719798	11.897249585	29.305401444
5.113719798	17.893849061	29.305401444
10.345919941	5.900649574	29.305401444
10.345919941	11.897249585	29.305401444
10.345919941	17.893849061	29.305401444
15.578119616	5.900649574	29.305401444
15.578119616	11.897249585	29.305401444
15.578119616	17.893849061	29.305401444
2.497610137	1.403179996	29.305401444
2.497610137	7.399779873	29.305401444
2.497610137	13.396379885	29.305401444
7.729810280	1.403179996	29.305401444
7.729810280	7.399779873	29.305401444
7.729810280	13.396379885	29.305401444
12.962009955	1.403179996	29.305401444
12.962009955	7.399779873	29.305401444
12.962009955	13.396379885	29.305401444
2.497610137	4.401480136	29.305401444
2.497610137	10.398079611	29.305401444
2.497610137	16.394680159	29.305401444
7.729810280	4.401480136	29.305401444
7.729810280	10.398079611	29.305401444
7.729810280	16.394680159	29.305401444
12.962009955	4.401480136	29.305401444
12.962009955	10.398079611	29.305401444
12.962009955	16.394680159	29.305401444
0.434074003	1.187329977	35.656398535
0.434074003	7.183929721	35.656398535
0.434074003	13.180529733	35.656398535
5.666273795	1.187329977	35.656398535
5.666273795	7.183929721	35.656398535
5.666273795	13.180529733	35.656398535
10.898473938	1.187329977	35.656398535
10.898473938	7.183929721	35.656398535
10.898473938	13.180529733	35.656398535
3.050169981	2.665719786	35.656398535
3.050169981	8.662319530	35.656398535
3.050169981	14.658919006	35.656398535
8.282369890	2.665719786	35.656398535
8.282369890	8.662319530	35.656398535
8.282369890	14.658919006	35.656398535
13.514569565	2.665719786	35.656398535
13.514569565	8.662319530	35.656398535
13.514569565	14.658919006	35.656398535
0.434074003	4.185619529	35.656398535

0.434074003	10.182219809	35.656398535
0.434074003	16.178819284	35.656398535
5.666273795	4.185619529	35.656398535
5.666273795	10.182219809	35.656398535
5.666273795	16.178819284	35.656398535
10.898473938	4.185619529	35.656398535
10.898473938	10.182219809	35.656398535
10.898473938	16.178819284	35.656398535
3.050169981	5.664010142	35.656398535
3.050169981	11.660610154	35.656398535
3.050169981	17.657209629	35.656398535
8.282369890	5.664010142	35.656398535
8.282369890	11.660610154	35.656398535
8.282369890	17.657209629	35.656398535
13.514569565	5.664010142	35.656398535
13.514569565	11.660610154	35.656398535
13.514569565	17.657209629	35.656398535
1.306100032	5.677840353	37.427300215
1.306100032	11.674440364	37.427300215
1.306100032	17.671039840	37.427300215
6.538300057	5.677840353	37.427300215
6.538300057	11.674440364	37.427300215
6.538300057	17.671039840	37.427300215
11.770500200	5.677840353	37.427300215
11.770500200	11.674440364	37.427300215
11.770500200	17.671039840	37.427300215
3.922199986	1.159659905	37.427300215
3.922199986	7.156259649	37.427300215
3.922199986	13.152859661	37.427300215
9.154399895	1.159659905	37.427300215
9.154399895	7.156259649	37.427300215
9.154399895	13.152859661	37.427300215
14.386599570	1.159659905	37.427300215
14.386599570	7.156259649	37.427300215
14.386599570	13.152859661	37.427300215
1.306100032	2.679559916	37.427300215
1.306100032	8.676159928	37.427300215
1.306100032	14.672759939	37.427300215
6.538300057	2.679559916	37.427300215
6.538300057	8.676159928	37.427300215
6.538300057	14.672759939	37.427300215
11.770500200	2.679559916	37.427300215
11.770500200	8.676159928	37.427300215
11.770500200	14.672759939	37.427300215
3.922199986	4.157949993	37.427300215

3.922199986	10.154549736	37.427300215
3.922199986	16.151149212	37.427300215
9.154399895	4.157949993	37.427300215
9.154399895	10.154549736	37.427300215
9.154399895	16.151149212	37.427300215
14.386599570	4.157949993	37.427300215
14.386599570	10.154549736	37.427300215
14.386599570	16.151149212	37.427300215
1.306100032	5.677840353	39.805400372
1.306100032	11.674440364	39.805400372
1.306100032	17.671039840	39.805400372
6.538300057	5.677840353	39.805400372
6.538300057	11.674440364	39.805400372
6.538300057	17.671039840	39.805400372
11.770500200	5.677840353	39.805400372
11.770500200	11.674440364	39.805400372
11.770500200	17.671039840	39.805400372
3.922199986	1.159659905	39.805400372
3.922199986	7.156259649	39.805400372
3.922199986	13.152859661	39.805400372
9.154399895	1.159659905	39.805400372
9.154399895	7.156259649	39.805400372
9.154399895	13.152859661	39.805400372
14.386599570	1.159659905	39.805400372
14.386599570	7.156259649	39.805400372
14.386599570	13.152859661	39.805400372
1.306100032	2.679559916	39.805498719
1.306100032	8.676159928	39.805498719
1.306100032	14.672759939	39.805498719
6.538300057	2.679559916	39.805498719
6.538300057	8.676159928	39.805498719
6.538300057	14.672759939	39.805498719
11.770500200	2.679559916	39.805498719
11.770500200	8.676159928	39.805498719
11.770500200	14.672759939	39.805498719
3.922199986	4.157949993	39.805400372
3.922199986	10.154549736	39.805400372
3.922199986	16.151149212	39.805400372
9.154399895	4.157949993	39.805400372
9.154399895	10.154549736	39.805400372
9.154399895	16.151149212	39.805400372
14.386599570	4.157949993	39.805400372
14.386599570	10.154549736	39.805400372
14.386599570	16.151149212	39.805400372

```
Ag/WSe2/Si
1.0
       15.9893999100         0.0000000000         0.0000000000
        0.0000000000        18.4659004211         0.0000000000
        0.0000000000         0.0000000000        50.0000000000
   Ag    W   Se   Si
  144   36   72  108
Cartesian
     0.000000000         0.000000000        20.000000298
     0.000000000         6.155300324        20.000000298
     0.000000000        12.310600648        20.000000298
     5.329800129         0.000000000        20.000000298
     5.329800129         6.155300324        20.000000298
     5.329800129        12.310600648        20.000000298
    10.659600258         0.000000000        20.000000298
    10.659600258         6.155300324        20.000000298
    10.659600258        12.310600648        20.000000298
     5.290209790         6.110600036        26.654100418
     5.290209790        12.265899810        26.654100418
     5.290209790        18.421200684        26.654100418
    10.620009919         6.110600036        26.654100418
    10.620009919        12.265899810        26.654100418
    10.620009919        18.421200684        26.654100418
    15.949809571         6.110600036        26.654100418
    15.949809571        12.265899810        26.654100418
    15.949809571        18.421200684        26.654100418
     0.000000000         3.077650162        20.000000298
     0.000000000         9.232950211        20.000000298
     0.000000000        15.388249984        20.000000298
     5.329800129         3.077650162        20.000000298
     5.329800129         9.232950211        20.000000298
     5.329800129        15.388249984        20.000000298
    10.659600258         3.077650162        20.000000298
    10.659600258         9.232950211        20.000000298
    10.659600258        15.388249984        20.000000298
     5.290209790         3.032949874        26.654100418
     5.290209790         9.188249923        26.654100418
     5.290209790        15.343550247        26.654100418
    10.620009919         3.032949874        26.654100418
    10.620009919         9.188249923        26.654100418
    10.620009919        15.343550247        26.654100418
    15.949809571         3.032949874        26.654100418
    15.949809571         9.188249923        26.654100418
    15.949809571        15.343550247        26.654100418
     1.762600047         6.139490538        22.230899334
```

1.762600047	12.294790862	22.230899334
1.762600047	18.450090635	22.230899334
7.092399819	6.139490538	22.230899334
7.092399819	12.294790862	22.230899334
7.092399819	18.450090635	22.230899334
12.422199947	6.139490538	22.230899334
12.422199947	12.294790862	22.230899334
12.422199947	18.450090635	22.230899334
1.762600047	3.061840101	22.230899334
1.762600047	9.217140425	22.230899334
1.762600047	15.372440198	22.230899334
7.092399819	3.061840101	22.230899334
7.092399819	9.217140425	22.230899334
7.092399819	15.372440198	22.230899334
12.422199947	3.061840101	22.230899334
12.422199947	9.217140425	22.230899334
12.422199947	15.372440198	22.230899334
3.525830055	6.124380217	24.449199438
3.525830055	12.279679991	24.449199438
3.525830055	18.434980865	24.449199438
8.855629946	6.124380217	24.449199438
8.855629946	12.279679991	24.449199438
8.855629946	18.434980865	24.449199438
14.185429598	6.124380217	24.449199438
14.185429598	12.279679991	24.449199438
14.185429598	18.434980865	24.449199438
3.525830055	3.046730055	24.449199438
3.525830055	9.202030104	24.449199438
3.525830055	15.357330428	24.449199438
8.855629946	3.046730055	24.449199438
8.855629946	9.202030104	24.449199438
8.855629946	15.357330428	24.449199438
14.185429598	3.046730055	24.449199438
14.185429598	9.202030104	24.449199438
14.185429598	15.357330428	24.449199438
4.427499516	1.523010067	22.230899334
4.427499516	7.678310253	22.230899334
4.427499516	13.833610027	22.230899334
9.757299645	1.523010067	22.230899334
9.757299645	7.678310253	22.230899334
9.757299645	13.833610027	22.230899334
15.087099297	1.523010067	22.230899334
15.087099297	7.678310253	22.230899334
15.087099297	13.833610027	22.230899334
4.427499516	4.600659816	22.230899334

4.427499516	10.755959590	22.230899334
4.427499516	16.911260464	22.230899334
9.757299645	4.600659816	22.230899334
9.757299645	10.755959590	22.230899334
9.757299645	16.911260464	22.230899334
15.087099297	4.600659816	22.230899334
15.087099297	10.755959590	22.230899334
15.087099297	16.911260464	22.230899334
0.860926953	1.507910065	24.449199438
0.860926953	7.663210389	24.449199438
0.860926953	13.818510162	24.449199438
6.190726784	1.507910065	24.449199438
6.190726784	7.663210389	24.449199438
6.190726784	13.818510162	24.449199438
11.520526436	1.507910065	24.449199438
11.520526436	7.663210389	24.449199438
11.520526436	13.818510162	24.449199438
0.860926953	4.585559952	24.449199438
0.860926953	10.740859725	24.449199438
0.860926953	16.896160599	24.449199438
6.190726784	4.585559952	24.449199438
6.190726784	10.740859725	24.449199438
6.190726784	16.896160599	24.449199438
11.520526436	4.585559952	24.449199438
11.520526436	10.740859725	24.449199438
11.520526436	16.896160599	24.449199438
2.664880051	1.538820128	20.000000298
2.664880051	7.694120039	20.000000298
2.664880051	13.849420913	20.000000298
7.994679941	1.538820128	20.000000298
7.994679941	7.694120039	20.000000298
7.994679941	13.849420913	20.000000298
13.324479593	1.538820128	20.000000298
13.324479593	7.694120039	20.000000298
13.324479593	13.849420913	20.000000298
2.625309964	1.494130021	26.654100418
2.625309964	7.649430208	26.654100418
2.625309964	13.804729981	26.654100418
7.955110093	1.494130021	26.654100418
7.955110093	7.649430208	26.654100418
7.955110093	13.804729981	26.654100418
13.284910222	1.494130021	26.654100418
13.284910222	7.649430208	26.654100418
13.284910222	13.804729981	26.654100418
2.664880051	4.616469877	20.000000298

2.664880051	10.771770476	20.000000298
2.664880051	16.927070250	20.000000298
7.994679941	4.616469877	20.000000298
7.994679941	10.771770476	20.000000298
7.994679941	16.927070250	20.000000298
13.324479593	4.616469877	20.000000298
13.324479593	10.771770476	20.000000298
13.324479593	16.927070250	20.000000298
2.625309964	4.571780046	26.654100418
2.625309964	10.727080645	26.654100418
2.625309964	16.882380418	26.654100418
7.955110093	4.571780046	26.654100418
7.955110093	10.727080645	26.654100418
7.955110093	16.882380418	26.654100418
13.284910222	4.571780046	26.654100418
13.284910222	10.727080645	26.654100418
13.284910222	16.882380418	26.654100418
0.764953071	1.403700060	31.070399284
0.764953071	7.558999971	31.070399284
0.764953071	13.714300295	31.070399284
6.094753022	1.403700060	31.070399284
6.094753022	7.558999971	31.070399284
6.094753022	13.714300295	31.070399284
11.424553150	1.403700060	31.070399284
11.424553150	7.558999971	31.070399284
11.424553150	13.714300295	31.070399284
0.764953071	4.481350084	31.070399284
0.764953071	10.636649858	31.070399284
0.764953071	16.791950732	31.070399284
6.094753022	4.481350084	31.070399284
6.094753022	10.636649858	31.070399284
6.094753022	16.791950732	31.070399284
11.424553150	4.481350084	31.070399284
11.424553150	10.636649858	31.070399284
11.424553150	16.791950732	31.070399284
3.429879881	6.020160444	31.070399284
3.429879881	12.175460218	31.070399284
3.429879881	18.330761092	31.070399284
8.759679533	6.020160444	31.070399284
8.759679533	12.175460218	31.070399284
8.759679533	18.330761092	31.070399284
14.089480138	6.020160444	31.070399284
14.089480138	12.175460218	31.070399284
14.089480138	18.330761092	31.070399284
3.429879881	2.942510007	31.070399284

3.429879881	9.097810331	31.070399284
3.429879881	15.253110655	31.070399284
8.759679533	2.942510007	31.070399284
8.759679533	9.097810331	31.070399284
8.759679533	15.253110655	31.070399284
14.089480138	2.942510007	31.070399284
14.089480138	9.097810331	31.070399284
14.089480138	15.253110655	31.070399284
2.540400072	1.402289985	32.849702239
2.540400072	7.557590034	32.849702239
2.540400072	13.712890358	32.849702239
7.870200201	1.402289985	32.849702239
7.870200201	7.557590034	32.849702239
7.870200201	13.712890358	32.849702239
13.199999853	1.402289985	32.849702239
13.199999853	7.557590034	32.849702239
13.199999853	13.712890358	32.849702239
2.540400072	4.479940147	32.849702239
2.540400072	10.635239921	32.849702239
2.540400072	16.790540795	32.849702239
7.870200201	4.479940147	32.849702239
7.870200201	10.635239921	32.849702239
7.870200201	16.790540795	32.849702239
13.199999853	4.479940147	32.849702239
13.199999853	10.635239921	32.849702239
13.199999853	16.790540795	32.849702239
5.205309905	6.018869928	32.849702239
5.205309905	12.174170252	32.849702239
5.205309905	18.329470025	32.849702239
10.535110034	6.018869928	32.849702239
10.535110034	12.174170252	32.849702239
10.535110034	18.329470025	32.849702239
15.864909686	6.018869928	32.849702239
15.864909686	12.174170252	32.849702239
15.864909686	18.329470025	32.849702239
5.205309905	2.941220041	32.849702239
5.205309905	9.096520365	32.849702239
5.205309905	15.251820689	32.849702239
10.535110034	2.941220041	32.849702239
10.535110034	9.096520365	32.849702239
10.535110034	15.251820689	32.849702239
15.864909686	2.941220041	32.849702239
15.864909686	9.096520365	32.849702239
15.864909686	15.251820689	32.849702239
2.542780057	1.404909953	29.307401180

2.542780057	7.560210140	29.307401180
2.542780057	13.715509913	29.307401180
7.872579948	1.404909953	29.307401180
7.872579948	7.560210140	29.307401180
7.872579948	13.715509913	29.307401180
13.202379600	1.404909953	29.307401180
13.202379600	7.560210140	29.307401180
13.202379600	13.715509913	29.307401180
2.542780057	4.482560528	29.307401180
2.542780057	10.637860577	29.307401180
2.542780057	16.793160350	29.307401180
7.872579948	4.482560528	29.307401180
7.872579948	10.637860577	29.307401180
7.872579948	16.793160350	29.307401180
13.202379600	4.482560528	29.307401180
13.202379600	10.637860577	29.307401180
13.202379600	16.793160350	29.307401180
5.207680122	6.021400330	29.307401180
5.207680122	12.176700654	29.307401180
5.207680122	18.332000428	29.307401180
10.537480251	6.021400330	29.307401180
10.537480251	12.176700654	29.307401180
10.537480251	18.332000428	29.307401180
15.867279903	6.021400330	29.307401180
15.867279903	12.176700654	29.307401180
15.867279903	18.332000428	29.307401180
5.207680122	2.943749893	29.307401180
5.207680122	9.099050217	29.307401180
5.207680122	15.254349990	29.307401180
10.537480251	2.943749893	29.307401180
10.537480251	9.099050217	29.307401180
10.537480251	15.254349990	29.307401180
15.867279903	2.943749893	29.307401180
15.867279903	9.099050217	29.307401180
15.867279903	15.254349990	29.307401180
0.442168000	1.218760015	36.044299603
0.442168000	7.374059926	36.044299603
0.442168000	13.529360800	36.044299603
5.771968010	1.218760015	36.044299603
5.771968010	7.374059926	36.044299603
5.771968010	13.529360800	36.044299603
11.101768139	1.218760015	36.044299603
11.101768139	7.374059926	36.044299603
11.101768139	13.529360800	36.044299603
3.107039831	2.736280023	36.044299603

3.107039831	8.891580346	36.044299603
3.107039831	15.046880120	36.044299603
8.436840198	2.736280023	36.044299603
8.436840198	8.891580346	36.044299603
8.436840198	15.046880120	36.044299603
13.766639850	2.736280023	36.044299603
13.766639850	8.891580346	36.044299603
13.766639850	15.046880120	36.044299603
0.442168000	4.296410314	36.044299603
0.442168000	10.451710363	36.044299603
0.442168000	16.607010137	36.044299603
5.771968010	4.296410314	36.044299603
5.771968010	10.451710363	36.044299603
5.771968010	16.607010137	36.044299603
11.101768139	4.296410314	36.044299603
11.101768139	10.451710363	36.044299603
11.101768139	16.607010137	36.044299603
3.107039831	5.813930460	36.044299603
3.107039831	11.969230783	36.044299603
3.107039831	18.124530557	36.044299603
8.436840198	5.813930460	36.044299603
8.436840198	11.969230783	36.044299603
8.436840198	18.124530557	36.044299603
13.766639850	5.813930460	36.044299603
13.766639850	11.969230783	36.044299603
13.766639850	18.124530557	36.044299603
1.330459960	5.828139895	37.771901488
1.330459960	11.983440219	37.771901488
1.330459960	18.138739993	37.771901488
6.660259851	5.828139895	37.771901488
6.660259851	11.983440219	37.771901488
6.660259851	18.138739993	37.771901488
11.990059980	5.828139895	37.771901488
11.990059980	11.983440219	37.771901488
11.990059980	18.138739993	37.771901488
3.995340011	1.190359992	37.771800160
3.995340011	7.345660316	37.771800160
3.995340011	13.500960640	37.771800160
9.325139663	1.190359992	37.771800160
9.325139663	7.345660316	37.771800160
9.325139663	13.500960640	37.771800160
14.654940269	1.190359992	37.771800160
14.654940269	7.345660316	37.771800160
14.654940269	13.500960640	37.771800160
1.330459960	2.750490009	37.771901488

1.330459960	8.905790333	37.771901488
1.330459960	15.061090656	37.771901488
6.660259851	2.750490009	37.771901488
6.660259851	8.905790333	37.771901488
6.660259851	15.061090656	37.771901488
11.990059980	2.750490009	37.771901488
11.990059980	8.905790333	37.771901488
11.990059980	15.061090656	37.771901488
3.995340011	4.268010154	37.771800160
3.995340011	10.423310203	37.771800160
3.995340011	16.578609976	37.771800160
9.325139663	4.268010154	37.771800160
9.325139663	10.423310203	37.771800160
9.325139663	16.578609976	37.771800160
14.654940269	4.268010154	37.771800160
14.654940269	10.423310203	37.771800160
14.654940269	16.578609976	37.771800160
1.330459960	5.828139895	40.160998702
1.330459960	11.983440219	40.160998702
1.330459960	18.138739993	40.160998702
6.660259851	5.828139895	40.160998702
6.660259851	11.983440219	40.160998702
6.660259851	18.138739993	40.160998702
11.990059980	5.828139895	40.160998702
11.990059980	11.983440219	40.160998702
11.990059980	18.138739993	40.160998702
3.995340011	1.190359992	40.160998702
3.995340011	7.345660316	40.160998702
3.995340011	13.500960640	40.160998702
9.325139663	1.190359992	40.160998702
9.325139663	7.345660316	40.160998702
9.325139663	13.500960640	40.160998702
14.654940269	1.190359992	40.160998702
14.654940269	7.345660316	40.160998702
14.654940269	13.500960640	40.160998702
1.330459960	2.750490009	40.160998702
1.330459960	8.905790333	40.160998702
1.330459960	15.061090656	40.160998702
6.660259851	2.750490009	40.160998702
6.660259851	8.905790333	40.160998702
6.660259851	15.061090656	40.160998702
11.990059980	2.750490009	40.160998702
11.990059980	8.905790333	40.160998702
11.990059980	15.061090656	40.160998702
3.995340011	4.268010154	40.160998702

3.995340011	10.423310203	40.160998702
3.995340011	16.578609976	40.160998702
9.325139663	4.268010154	40.160998702
9.325139663	10.423310203	40.160998702
9.325139663	16.578609976	40.160998702
14.654940269	4.268010154	40.160998702
14.654940269	10.423310203	40.160998702
14.654940269	16.578609976	40.160998702

Frictional Properties

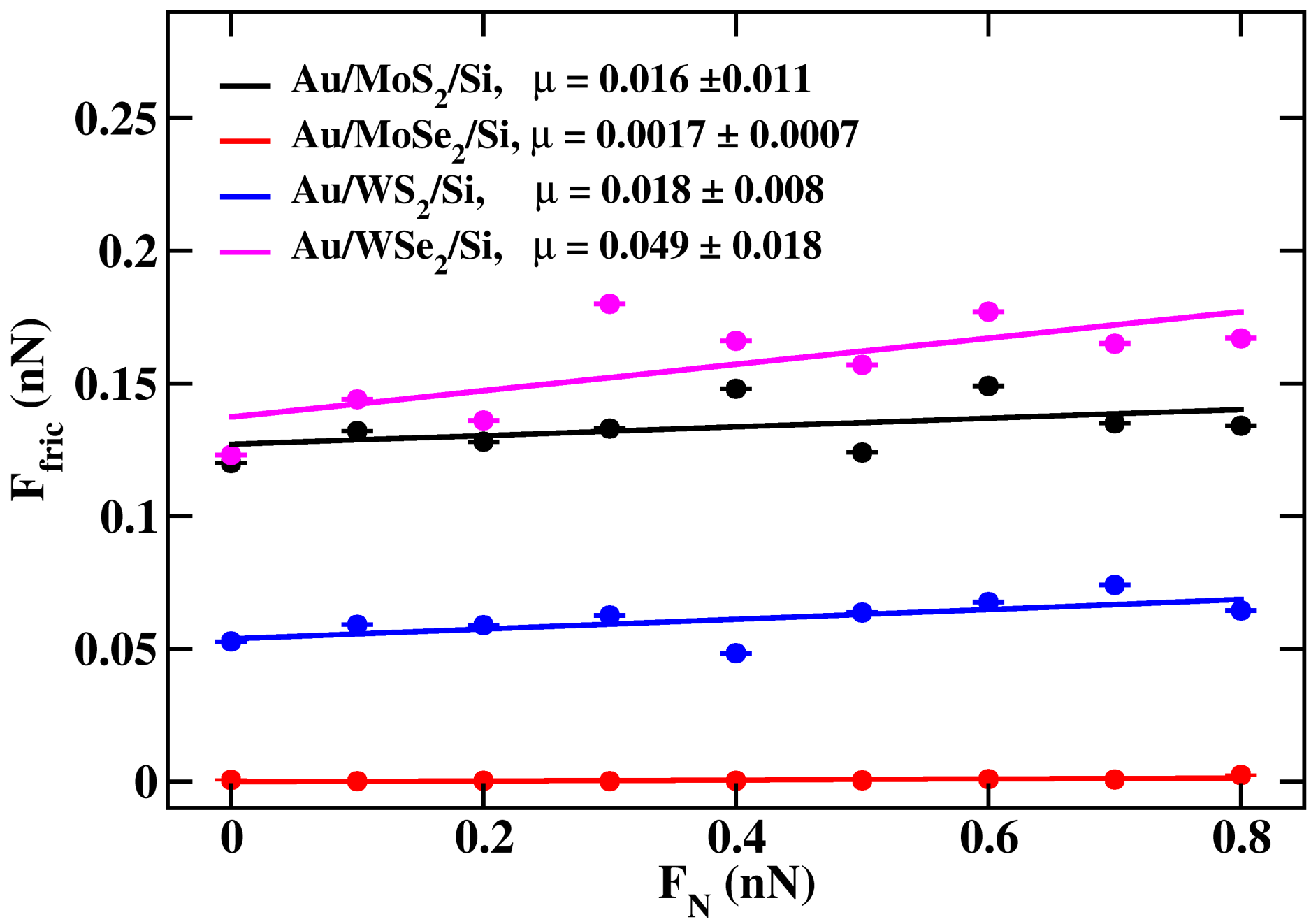

Figure S1: Linear fit of average friction force as a function of load with a tip velocity of 3 m/s.

Figure S1, Figure S2 and Figure S3 show friction force as a function of normal load for four Au/MX_2/Si systems expect Au/MoS_2/Si which is presented in the main manuscript, with linear fits used to extract the coefficient of friction μ. Most of the systems exhibhit nonmonotonic nature with loads and extraction of μ from a linear fit results in large errors. This same trend is observed in Ag/MX_2/Si systems as shown in Figure S6, Figure S7, Figure S8 and Figure S9.
Table S1, Table S2, Table S3, and Table S4 summarize the fitted values of the asymptotic friction coefficient (μ_∞) and the adhesion force parameter (A), along with their corresponding absolute errors, which are on the order of 10^{-4}. The TM/MoS_2/Si and TM/WSe_2/Si systems exhibit the highest values of the adhesion parameter A among all the considered systems and across all sliding velocities. This

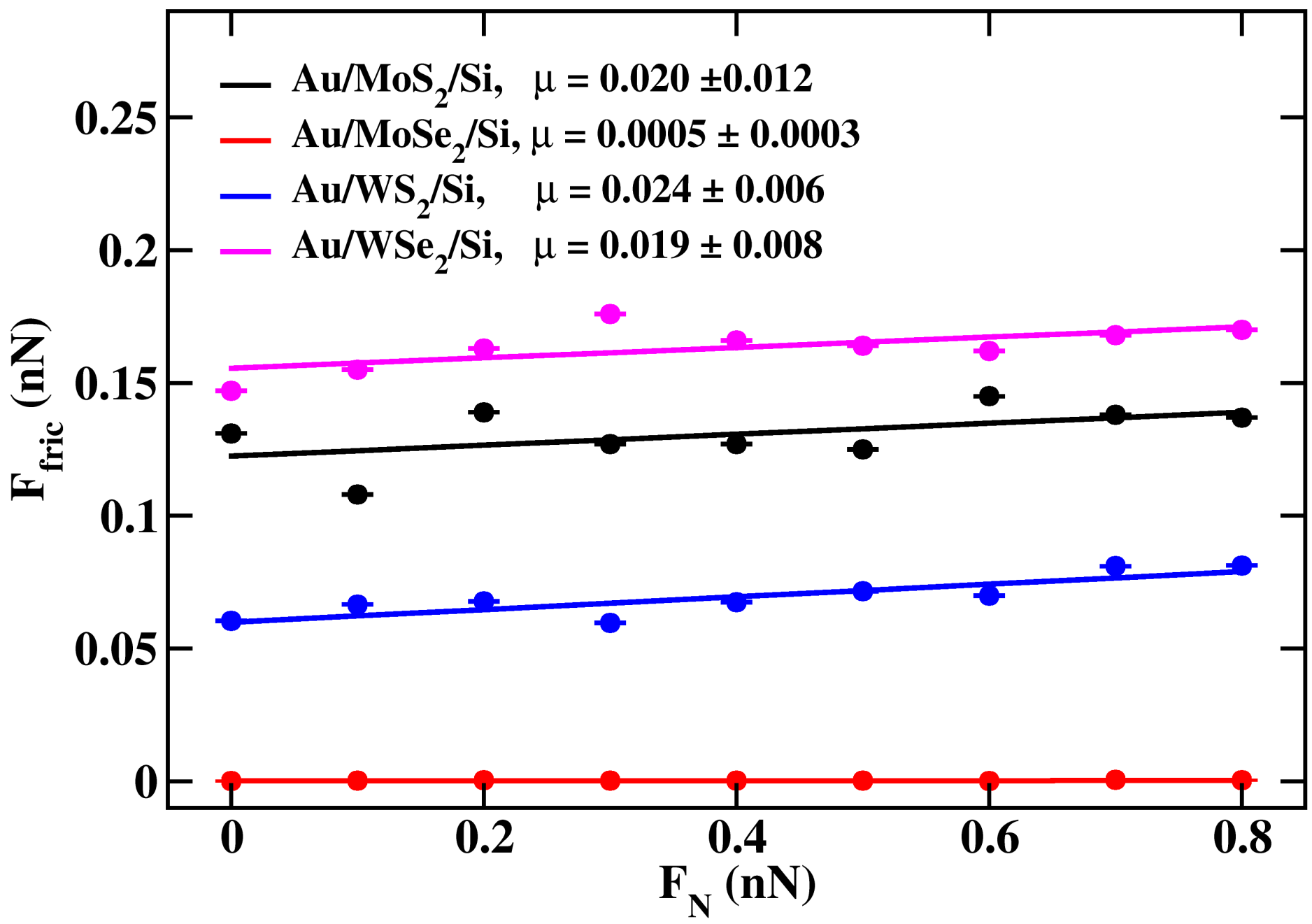

Figure S2: Linear fit of average friction force as a function of load with a tip velocity of 4 m/s.

behaviour indicates enhanced interfacial adhesion, reflecting stronger adhesive interactions between the contacting surfaces relative to the other systems studied.
The friction force profile for the Au/$MoSe_2$/Si system is shown in Figure S4. In this case, both lateral slip and zigzag behaviour are suppressed and the tip moves along $\boldsymbol{y}$-direction. This is also observed in the Fourier transform spectra of Au/$MoSe_2$/Si where the second and third peaks, representing the lateral and ziz-zag motions, are absent (Figure S5).
Figure S10 shows the spatial Fourier transform of the frictional force in the Ag/MX_2/Si and Au/MX_2/Si systems. In the Au/WSe_2/Si case, the first peak dominates than the Ag/WSe_2/Si system which leads to higher frictional force.
Figure S11 shows the Fourier transform of friction force as a function of different velocity for Au/MoS_2/Si system. As we can see that the peak positions are identical for all the velocity confirming that the lateral slip and zig-zag motions are intrinsic features of the potential energy surface topology rather than artifacts of increasing sliding velocity.
Figure S12 shows the calculated μ' over each load as a function load for Au/MX_2/Si system with tip velocity of 2 m/s. The plot shows the decreasing trend of μ' by increasing the load, indicating a departure from the classical behaviour at the nanoscale.

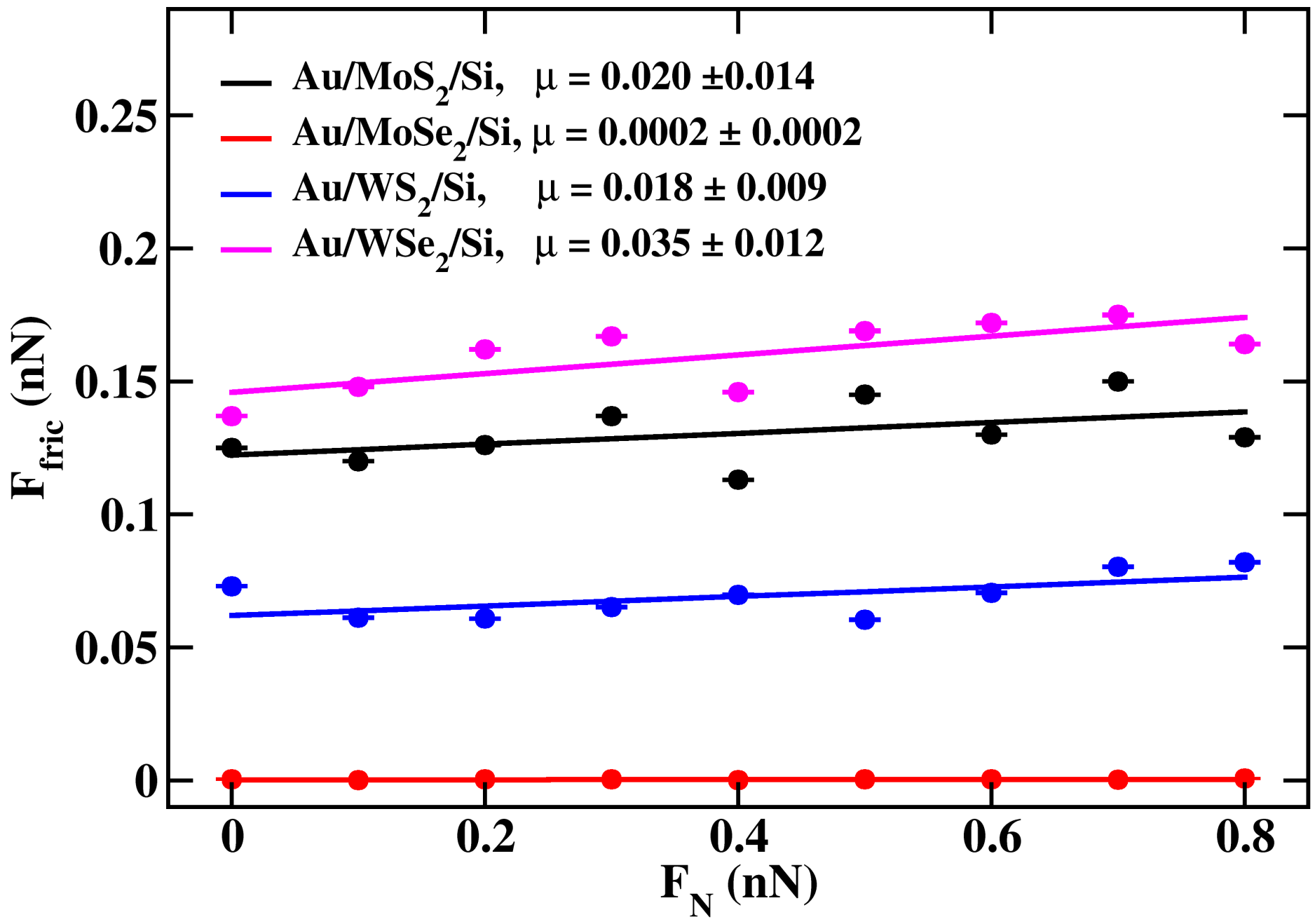

Figure S3: Linear fit of average friction force as a function of load with a tip velocity of 5 m/s.

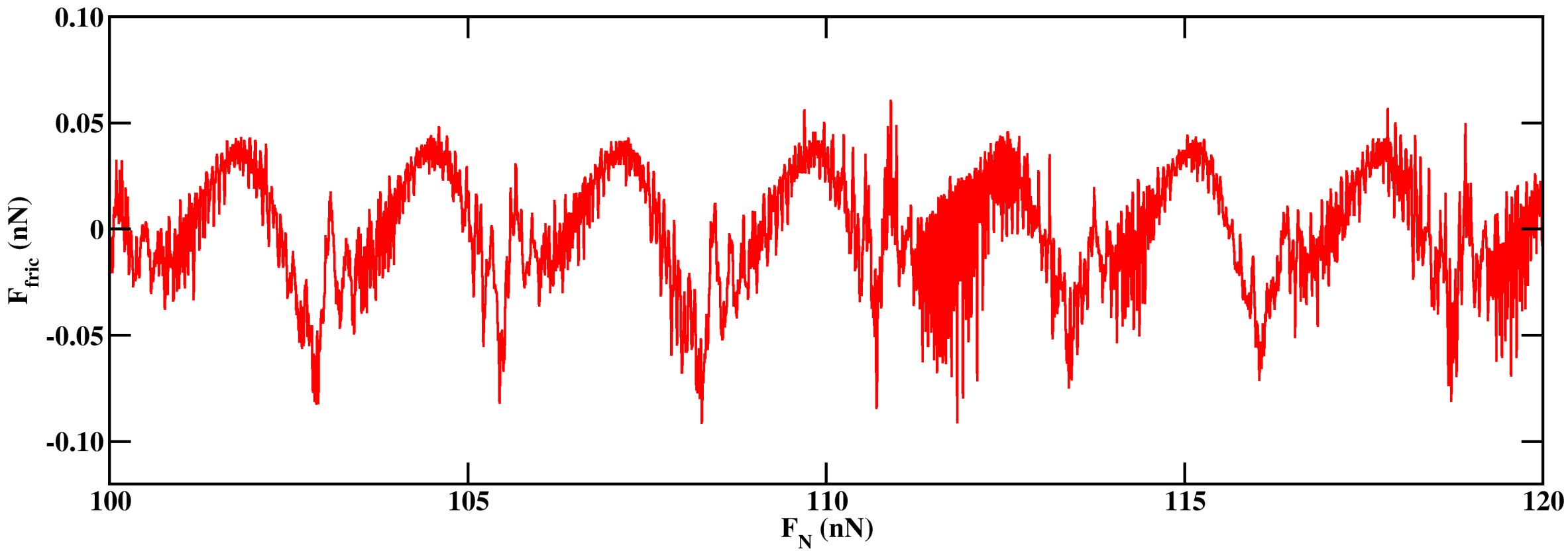

Figure S4: Friction force profile of Au/$MoSe_2$/Si where tip moving with velocity 2 m/s for load of 0 nN.

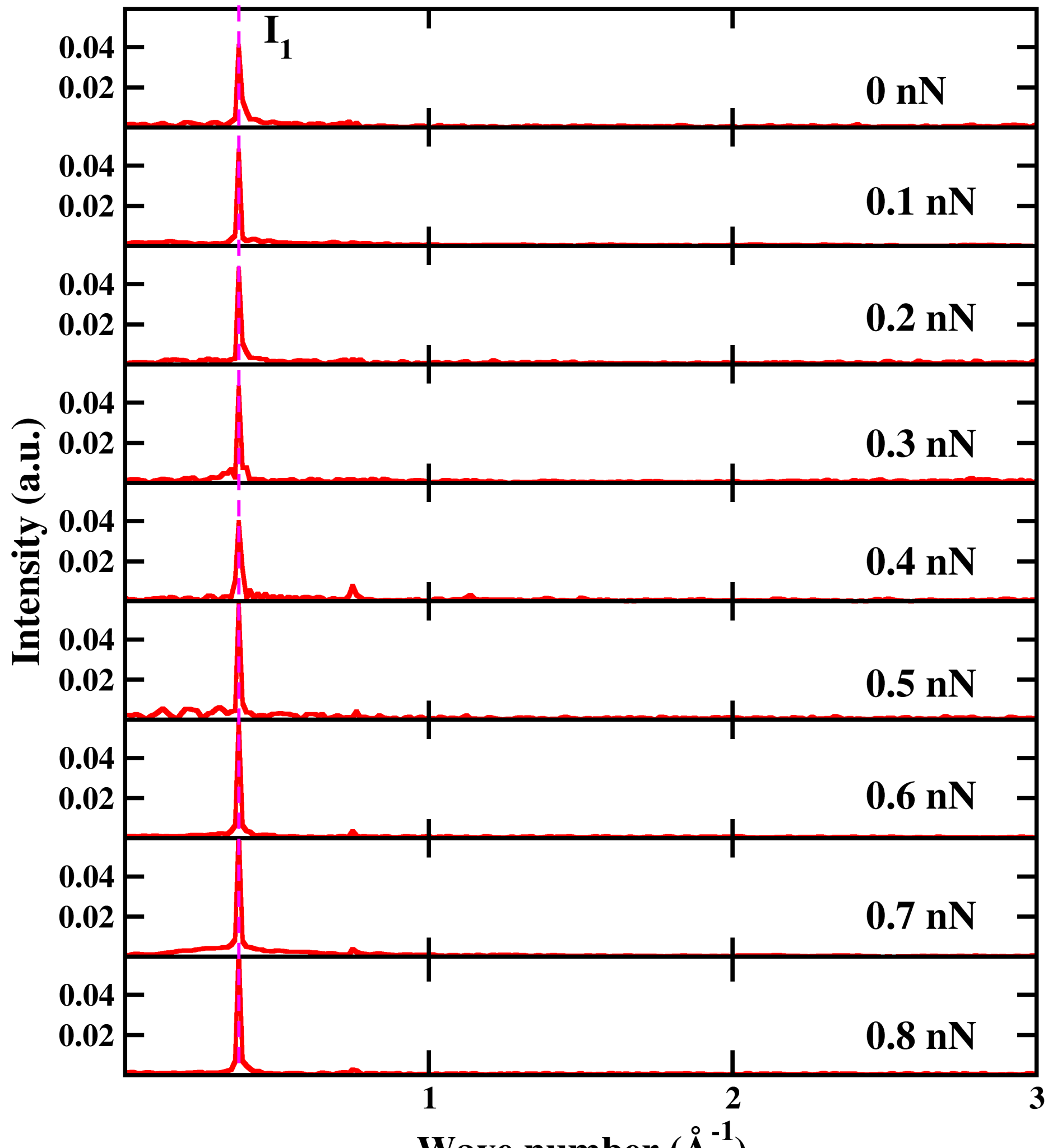

Figure S5: Spatial Fourier transform of Au/$MoSe_2$/Si with varying loads at velocity 2 m/s.

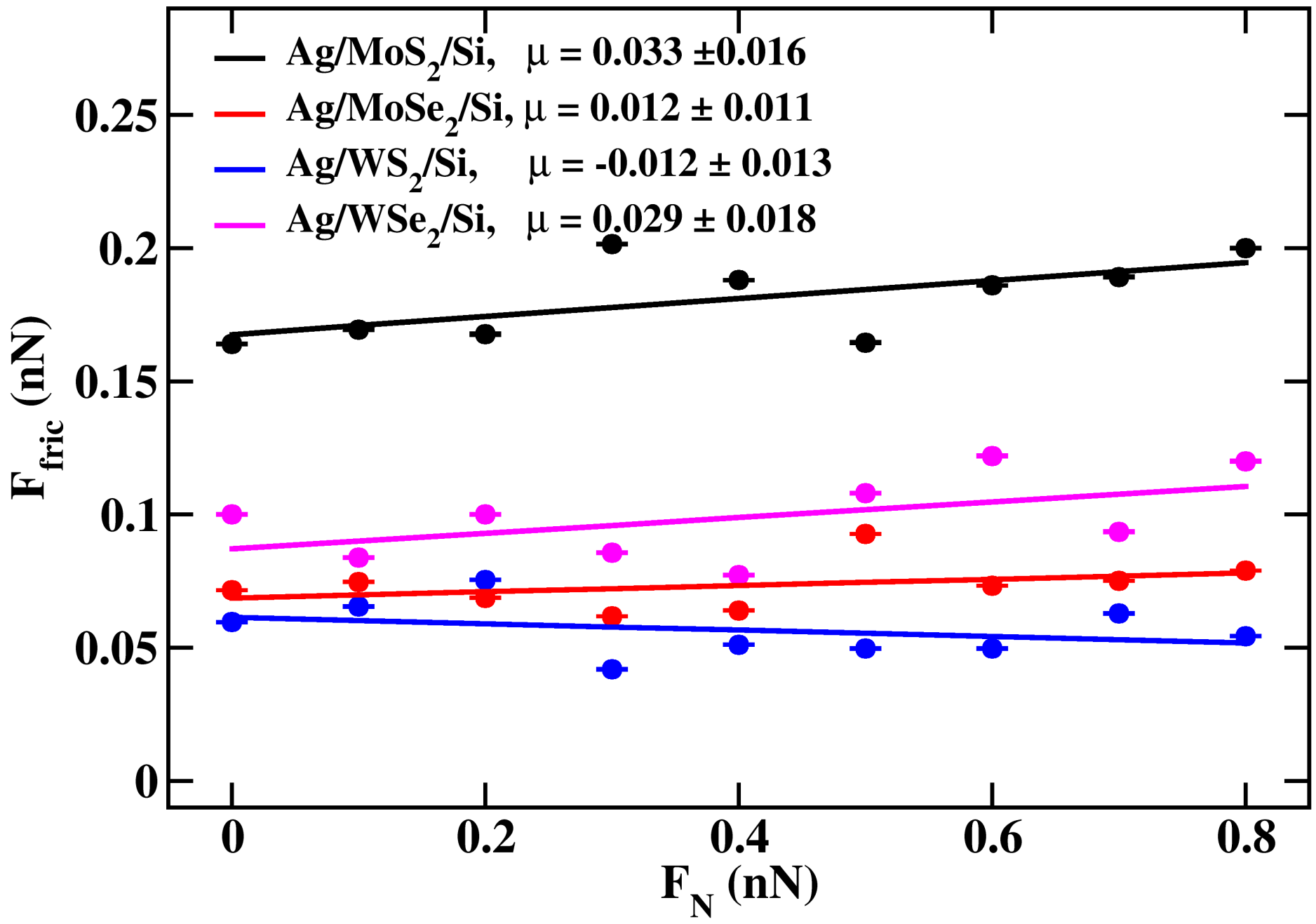

Figure S6: μ extracted from a linear fit on the average friction force as a function of load with a tip velocity of 2 m/s.

Table S1: Fitting parameters extracted from simulations with tip velocity equal to 2 m/s.

**Monolayer**	**Substrate**	$\mu_\infty (10^{-4})$	A $(10^{-4}$ nN)
MoS_2	Au	415 ± 4	997 ± 2
$MoSe_2$		4.0 ± 0.4	5.2 ± 0.2
WS_2		229 ± 4	569 ± 2
WSe_2		894 ± 4	1046 ± 2
MoS_2	Ag	292 ± 5	1707 ± 3
$MoSe_2$		87 ± 3	704 ± 2
WS_2		-63 ± 4	571 ± 2
WSe_2		555 ± 4	717 ± 2

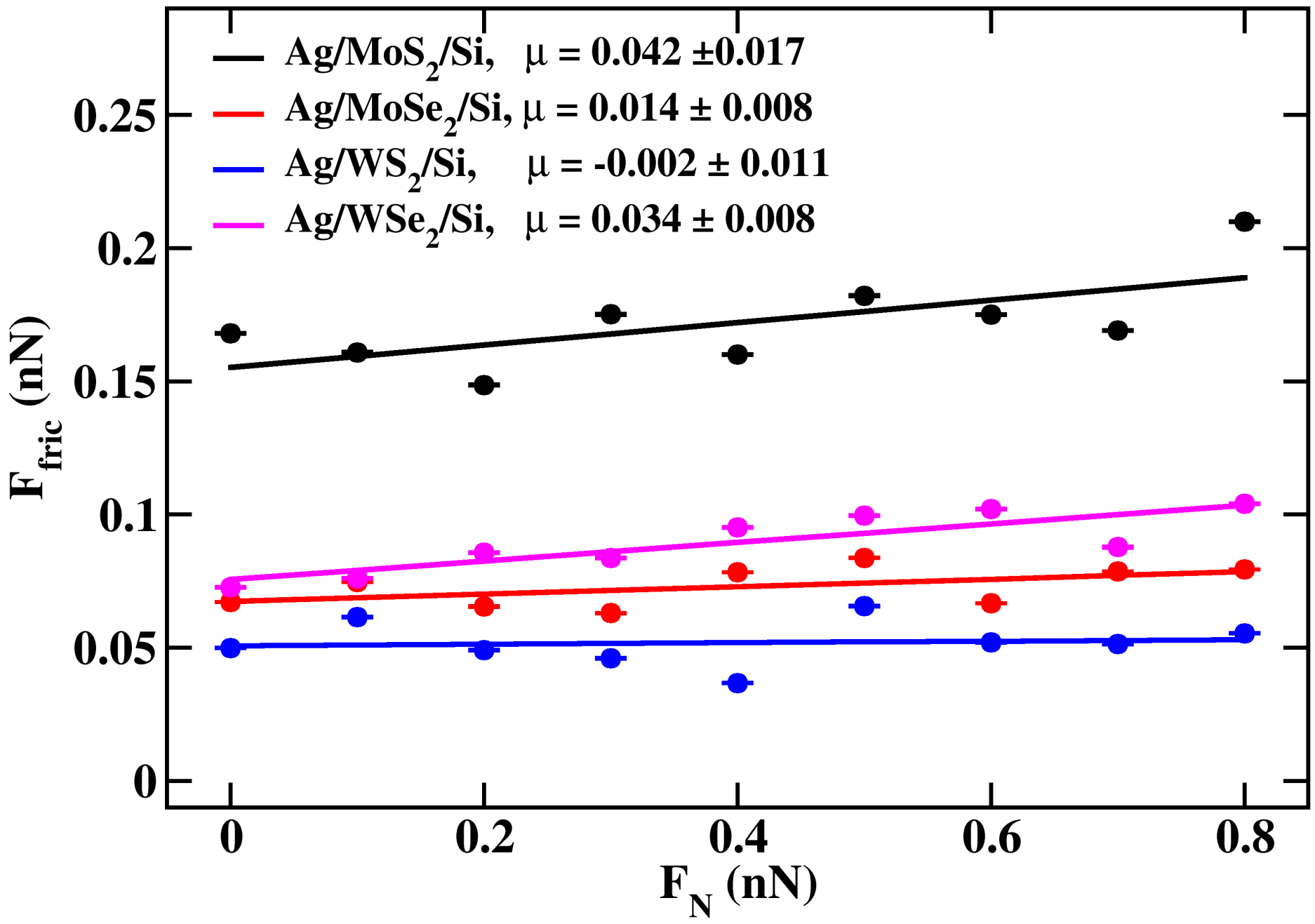

Figure S7: μ extracted from a linear fit function on the average friction force as a function of load with a tip velocity of 3 m/s.

Table S2: Fitting parameters extracted from simulations with tip velocity equal to 3 m/s.

**Monolayer**	**Substrate**	$\mu_\infty (10^{-4})$	A $(10^{-4}$ nN)
MoS_2	Au	79 ± 4	1317 ± 2
$MoSe_2$	Au	20.1 ± 0.4	-3.3 ± 0.2
WS_2	Au	167 ± 4	547 ± 2
WSe_2	Au	367 ± 5	1447 ± 3
MoS_2	Ag	565 ± 5	1474 ± 3
$MoSe_2$	Ag	109.2 ± 0.3	688 ± 2
WS_2	Ag	20 ± 4	506 ± 2
WSe_2	Ag	336 ± 3	770 ± 2

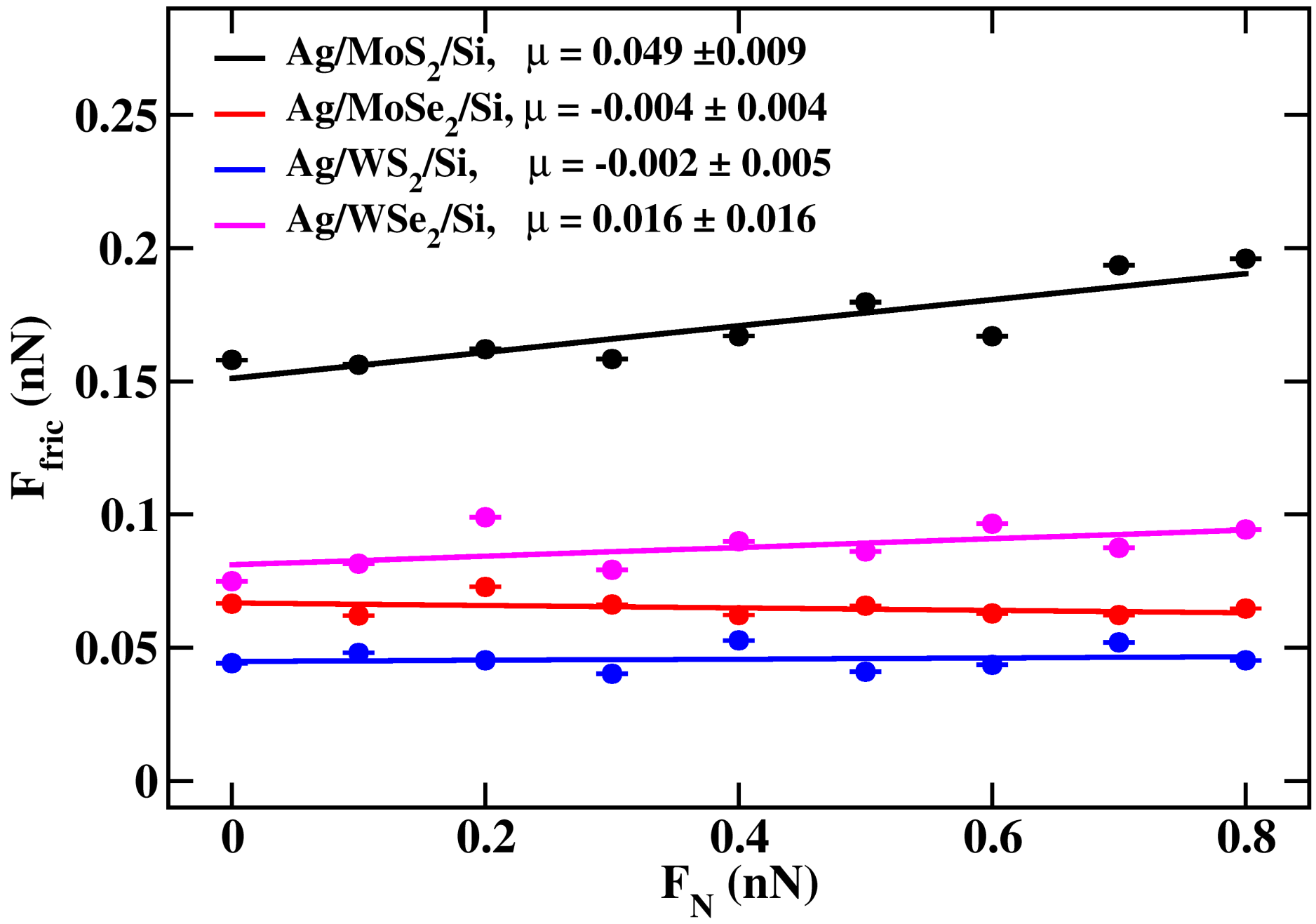

Figure S8: μ extracted from a linear fit function on the average friction force as a function of load with a tip velocity of 4 m/s.

Table S3: Fitting parameters extracted from simulations with tip velocity equal to 4 m/s.

**Monolayer**	**Substrate**	$\mu_\infty (10^{-4})$	A (10^{-4} nN)
MoS_2	Au	323 ± 4	1164 ± 2
$MoSe_2$		4.6 ± 0.4	0.9 ± 0.2
WS_2		237 ± 5	599 ± 2
WSe_2		113 ± 5	1603 ± 2
MoS_2	Ag	563 ± 5	1464 ± 3
$MoSe_2$		-12 ± 2	693 ± 2
WS_2		7 ± 3	457 ± 2
WSe_2		152 ± 3	818 ± 2

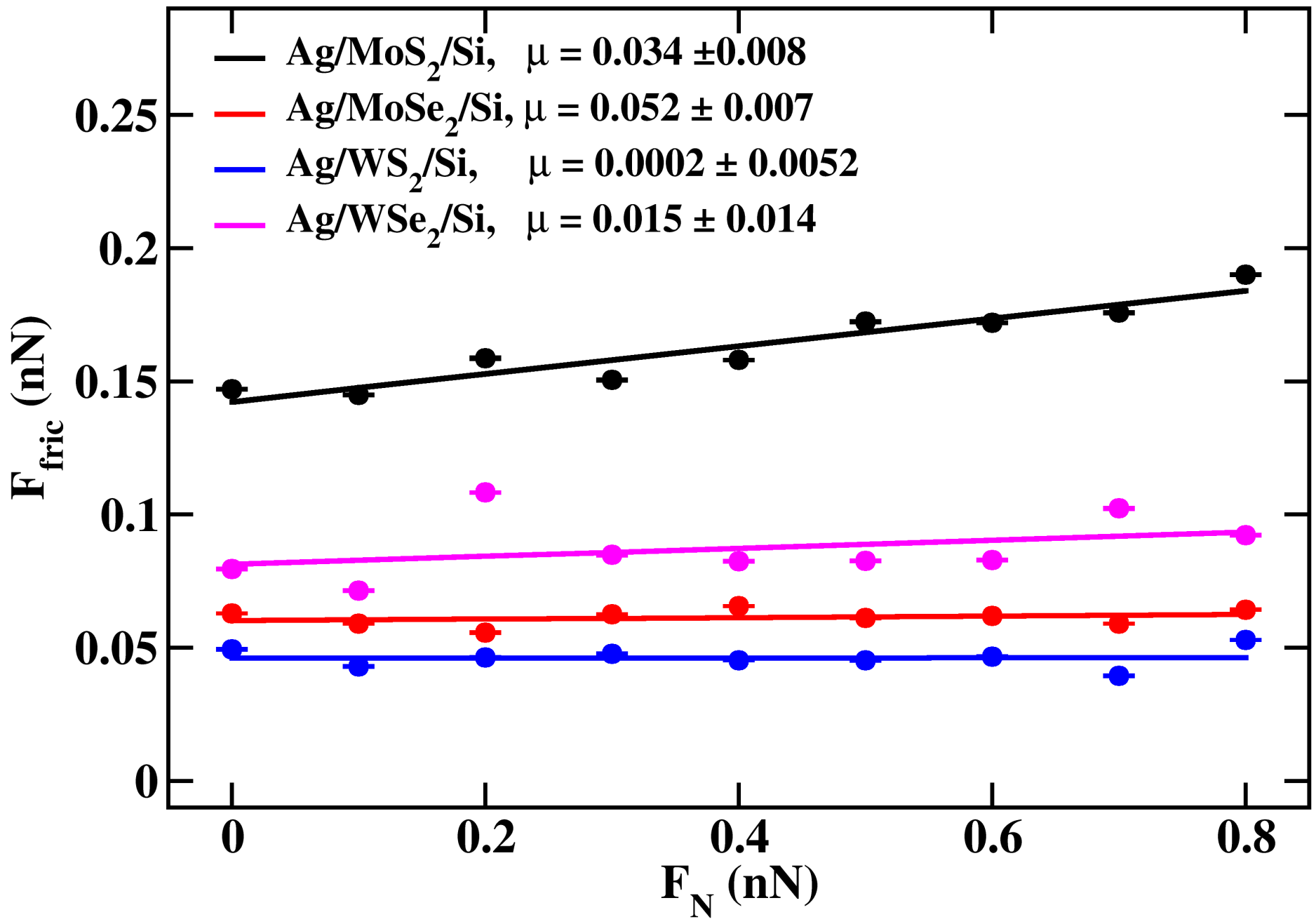

Figure S9: μ extracted from a linear fit function on the average friction force as a function of load with a tip velocity of 5 m/s. Ag/WS_2/Si and Ag/WSe_2/Si systems shows comparatively higher errors than other systems.

Table S4: Fitting parameters extracted from simulations with tip velocity equal to 5 m/s.

**Monolayer**	**Substrate**	$\mu_\infty (10^{-4})$	A (10^{-4} nN)
MoS_2	Au	224 ± 4	1208 ± 2
$MoSe_2$	Au	2.9 ± 0.4	2.0 ± 0.2
WS_2	Au	229 ± 4	569 ± 2
WSe_2	Au	262 ± 5	1510 ± 3
MoS_2	Ag	608 ± 5	1373 ± 3
$MoSe_2$	Ag	44 ± 3	597 ± 2
WS_2	Ag	50 ± 4	436 ± 2
WSe_2	Ag	220 ± 4	758 ± 2

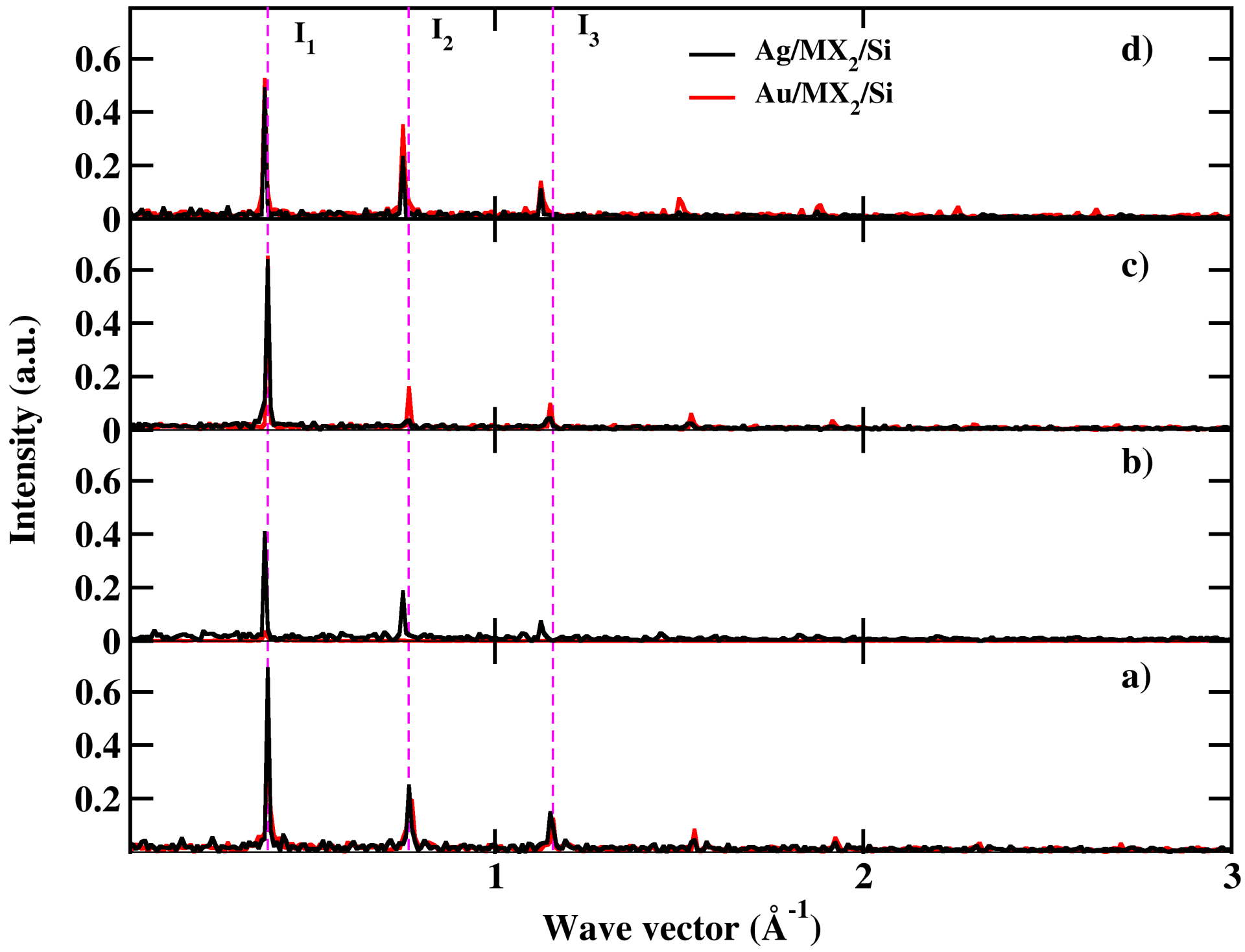

Figure S10: Spatial Fourier transform of friction force with monolayer a) MoS_2, b) $MoSe_2$, c) WS_2 and d) WSe_2 at different substrates.

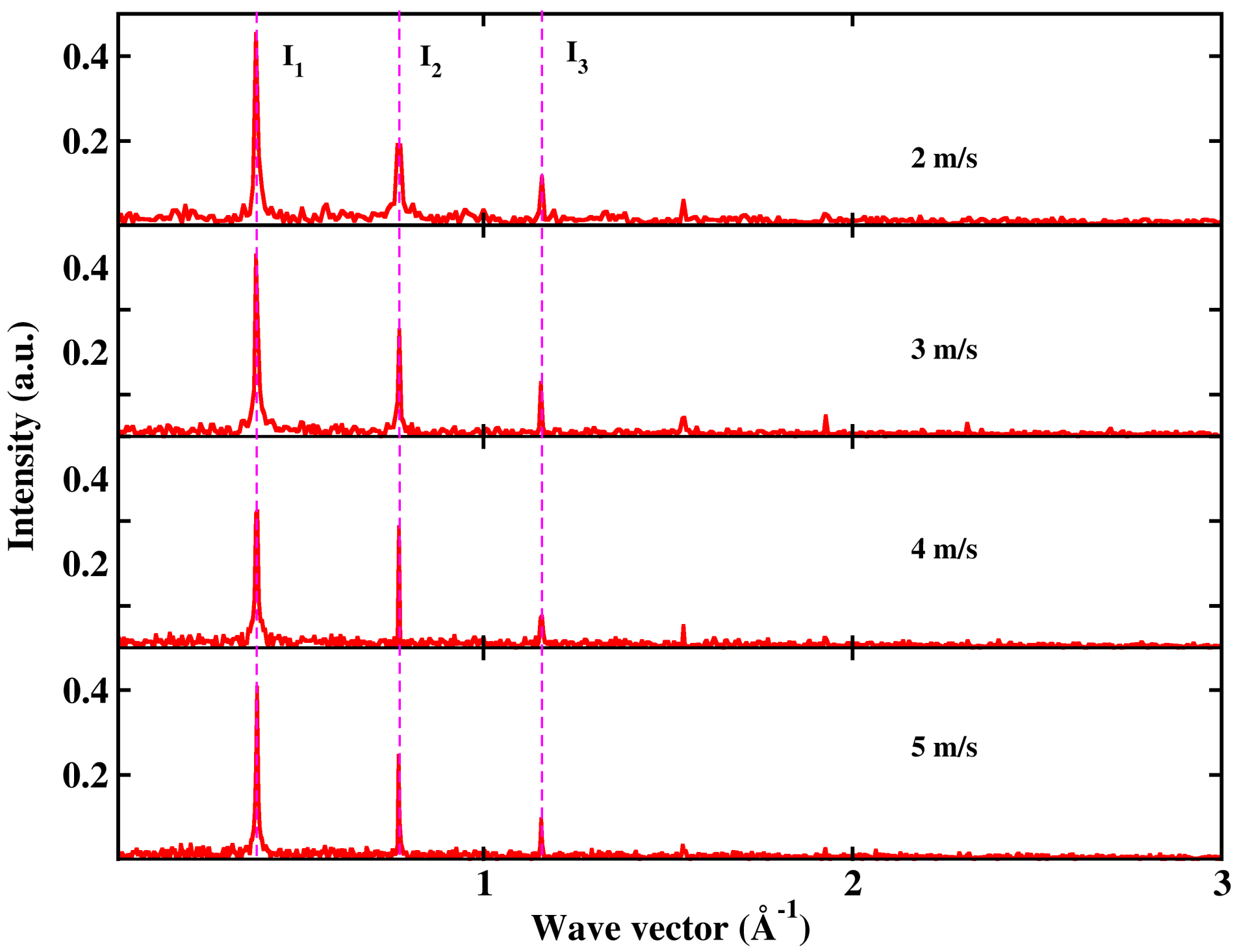

Figure S11: Spatial Fourier transform of friction force of Au/MoS_2/Si under the load of 0.4 nN with different velocity

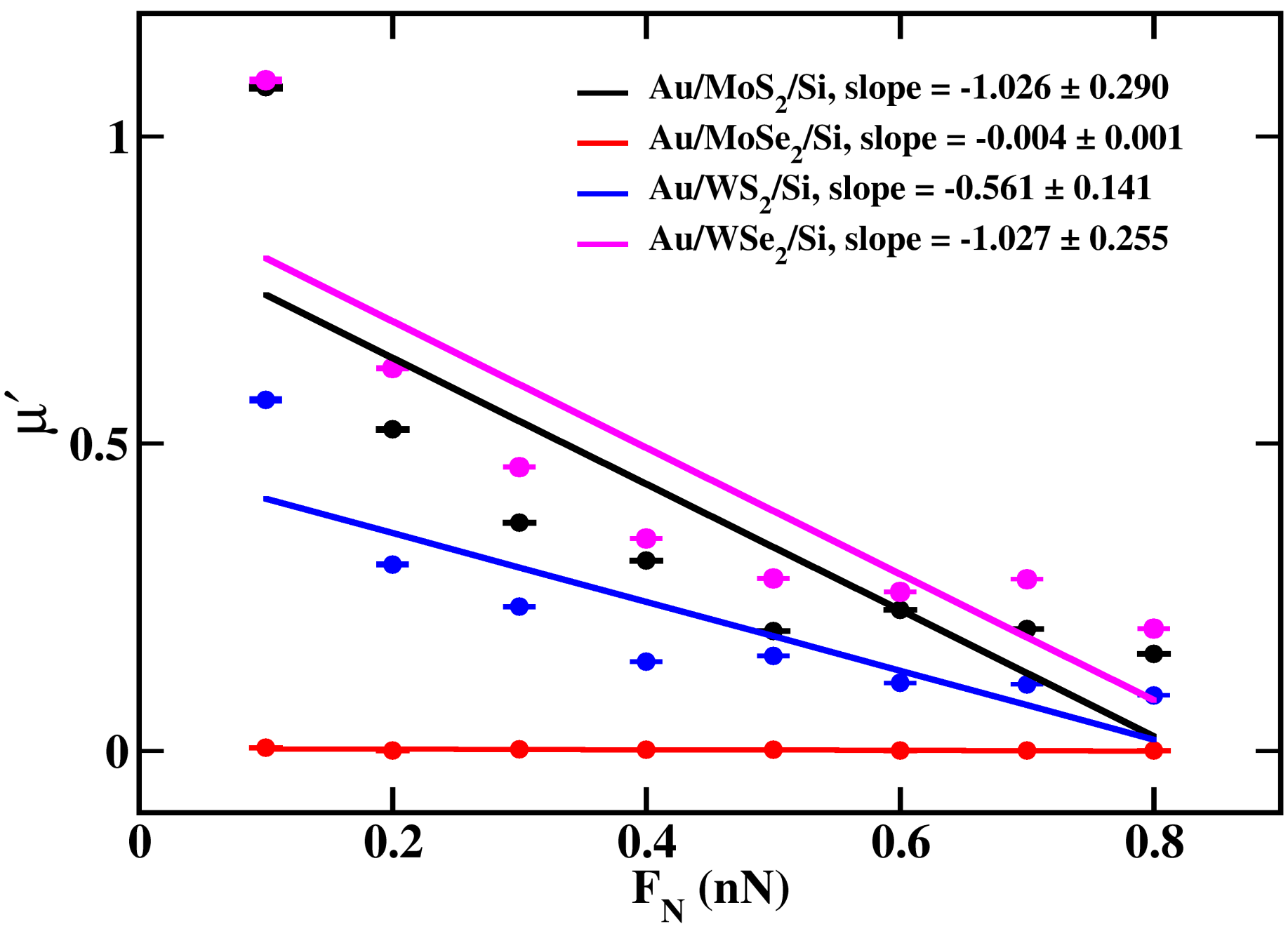

Figure S12: Calculated μ' over each load plotted as a function of load for the Au/MX_2/Si system with tip velocity of 2 m/s.
